# Pendant Group Modifications Provide Graft Copolymer Silicones with Exceptionally Broad Thermomechanical Properties

**DOI:** 10.1021/acscentsci.2c01246

**Published:** 2022-12-23

**Authors:** Keith E. L. Husted, Abraham Herzog-Arbeitman, Denise Kleinschmidt, Wenxu Zhang, Zehao Sun, Alyssa J. Fielitz, An N. Le, Mingjiang Zhong, Jeremiah A. Johnson

**Affiliations:** † Department of Chemistry, Massachusetts Institute of Technology, Cambridge, Massachusetts 02139, United States; ‡ Department of Materials Science and Engineering, Massachusetts Institute of Technology, Cambridge, Massachusetts 02139, United States; § Core R&D, Analytical Science, The Dow Chemical Company, Midland, Michigan 48640, United States; ∥ Department of Chemical and Environmental Engineering, Yale University, New Haven, Connecticut 06520, United States

## Abstract

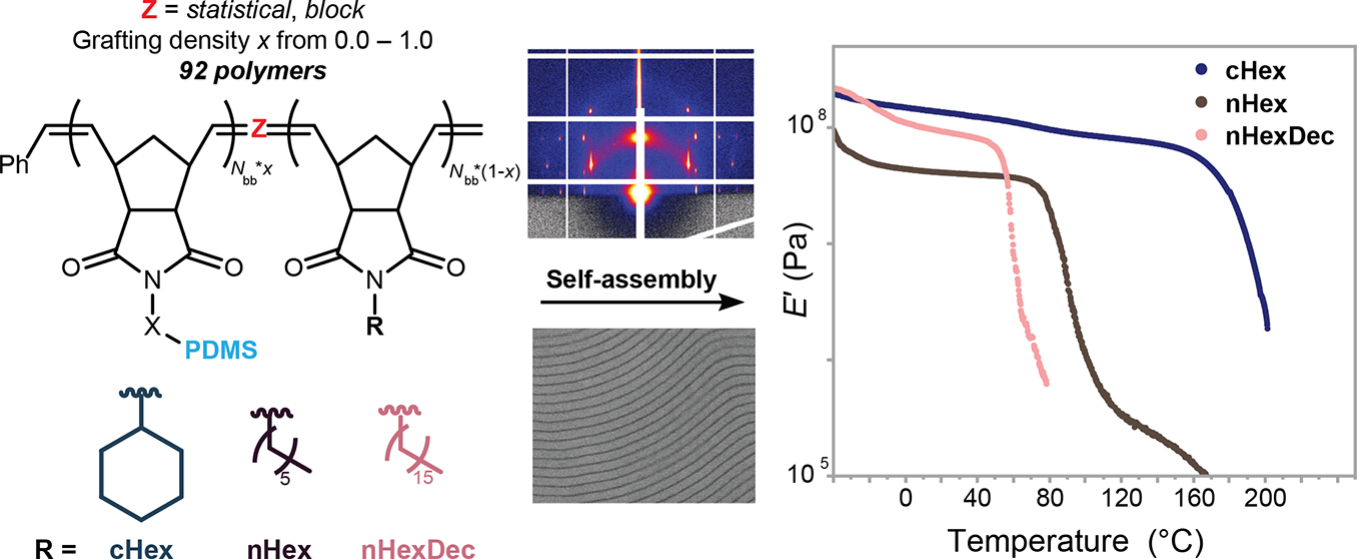

Graft copolymers offer a versatile platform for the design of self-assembling materials; however, simple strategies for precisely and independently controlling the thermomechanical and morphological properties of graft copolymers remain elusive. Here, using a library of 92 polynorbornene-*graft*-polydimethylsiloxane (PDMS) copolymers, we discover a versatile backbone-pendant sequence-control strategy that addresses this challenge. Small structural variations of pendant groups, e.g., cyclohexyl versus *n*-hexyl, of small-molecule comonomers have dramatic impacts on order-to-disorder transitions, glass transitions, mechanical properties, and morphologies of statistical and block silicone-based graft copolymers, providing an exceptionally broad palette of designable materials properties. For example, statistical graft copolymers with high PDMS volume fractions yielded unbridged body-centered cubic morphologies that behaved as soft plastic crystals. By contrast, lamellae-forming graft copolymers provided robust, yet reprocessable silicone thermoplastics (TPs) with transition temperatures spanning over 160 °C and elastic moduli as high as 150 MPa despite being both unentangled and un-cross-linked. Altogether, this study reveals a new pendant-group-mediated self-assembly strategy that simplifies graft copolymer synthesis and enables access to a diverse family of silicone-based materials, setting the stage for the broader development of self-assembling materials with tailored performance specifications.

## Abstract 2

Small structural variations to backbone-pendant groups in a library of graft copolymers are shown to enable orthogonal control of a broad array of morphological and thermomechanical properties.

Graft copolymers, which feature polymeric side chains attached to a polymer backbone, offer greater structural complexity than traditional linear (co)polymers, with parameters such as backbone length, side chain sequence,^1−5^ side chain length,^6^ grafting density,^7,8^ and block lengths^9^ defining their bulk properties^10−14^ and self-assembled morphologies^15−21^ and enabling their application as optical materials,^22,23^ elastomers,^24−28^ drug delivery systems,^29,30^ biomimetics,^27,31−34^ and more.^35−38^ Nevertheless, despite significant recent interest in their synthesis and properties, most studies of graft copolymers to date have explored variation of only one of these physical parameters in order to make property comparisons. Moreover, the majority of these studies involved either homopolymers or copolymers derived from two different polymer side chains arranged in either block or statistical sequences; the potential for combining graft polymer segments with small-molecule comonomers, leveraging the latter to tune material properties, has been much less explored.

Sheiko and co-workers reported A-B-A triblock copolymers with linear polymer A blocks and a graft copolymer B block; these structures formed very soft thermoplastic elastomers through microphase separation of their A blocks into spherical morphologies bridged by the B segment.^33^ Bates and co-workers reported statistical graft copolymers featuring polydimethylsiloxane (PDMS) and smaller, oligo(ethylene oxide) (OEO) side chains; intramolecular phase separation of the PDMS and OEO segments yielded very soft, shear-thinning materials with unbridged spherical morphologies.^39^ In a different vein, Grubbs and co-workers explored the impact of small-molecule monomers as “diluents” in the formation of diblock bottlebrush copolymers, revealing how tapered and gradient side chains can impact diblock bottlebrush assembly. While inspiring, these disparate studies do not explore the impact of comonomer composition on the thermomechanical properties and morphologies of the materials investigated, relying primarily on tuning dimensions of the grafted chains to modulate material characteristics. Moreover, these studies do not provide access to materials possessing a wide range of thermomechanical properties.

Recently, powerful systematic syntheses of multicomponent or multiparameter polymer libraries have been used to elucidate fundamental physical processes and shed new light on materials design that would not be possible with limited sample sizes.^40−43^ Herein, we present a systematic synthesis approach to construct a library of both block and statistical graft copolymers spanning multiple macromolecular parameters, leveraging the well-established ring-opening metathesis polymerization (ROMP) of norbornene derivatives. Most importantly, alongside the simultaneous multidimensional exploration of grafting density, sequence, and backbone degree of polymerization, we utilize three different small-molecule comonomers that are shown to have major impacts on the achievable morphologies and thermomechanical performance of the materials. This straightforward systematic synthetic platform is demonstrated to enable rapid exploration and discovery of graft copolymer-based silicone materials with diverse, bespoke, and in some cases remarkable properties.

Silicones are an important category of commercial polymers that find applications as lubricants, elastomers, sealants, components of household appliances, dielectrics, and more. Despite their broad utility, however, silicones suffer from low moduli and difficult-to-tune thermal transitions, unless they are densely cross-linked or filled, two strategies that can be prohibitive in terms of material (re)processing, flexibility, and optical properties (e.g., transparency). These drawbacks motivated our interest in designing a modular and efficient synthetic approach to broadly tune silicone properties using a simple copolymerization strategy. Through ROMP of an *exo*-norbornene-terminated PDMS macromonomer combined with one of three comonomers featuring aliphatic side chains (*n*-hexyl, cyclohexyl, or *n*-hexadecyl), a library of 92 polymers was prepared (Figure 1a). Bulk samples from this library displayed a striking range of properties. For example, statistical copolymers with very high PDMS weight fractions (up to *f*_*PDMS-MM*_ = 0.98) behaved as shear-thinning, soft (*E*′ ≈ 100 kPa) plastic crystals with body-centered cubic (BCC) spherical morphologies. By contrast, statistical copolymers with lower PDMS volume fractions (e.g., *f_PDMS-MM_* = 0.53) formed transparent and reprocessable TPs with lamellar morphologies, broad processing windows (over 160 °C), and room-temperature elastic moduli (*E*′) ranging from 10 to 150 MPa, which are greater than reported values for unfilled commercial cross-linked silicone elastomers (e.g., Sylgard 184 with *E*′ of 1.7 MPa).^44^ Moreover, these graft copolymer silicones can be easily reprocessed to provide new materials with equivalent mechanical performance, offering a recyclable alternative to cross-linked silicone thermosets. Most strikingly, the pendant groups of the small-molecule comonomers are shown to play dominant roles in defining the thermomechanical and morphological properties of these materials, while domain spacing relationships are independently dictated by grafting density (defined as the average number of grafted side chains per backbone degree of polymerization^8^) and backbone sequence. Therefore, while some of the trends between physical parameters and polymer properties observed here corroborate established literature (such as the decrease in modulus with increasing grafting density and dependence of entanglement molar mass on grafting density),^45^ we find that comonomer pendant-group variation can provide a seamlessly compatible and orthogonal strategy for thermomechanical property tuning. Finally, we observe that statistical copolymers, which are generated in a single polymerization step, offer materials with improved thermomechanical properties compared to block copolymers of the same *f*_*PDMS-MM*_, which facilitates the manufacturing of these materials as demonstrated through decagram-scale syntheses.

**Figure 1:**
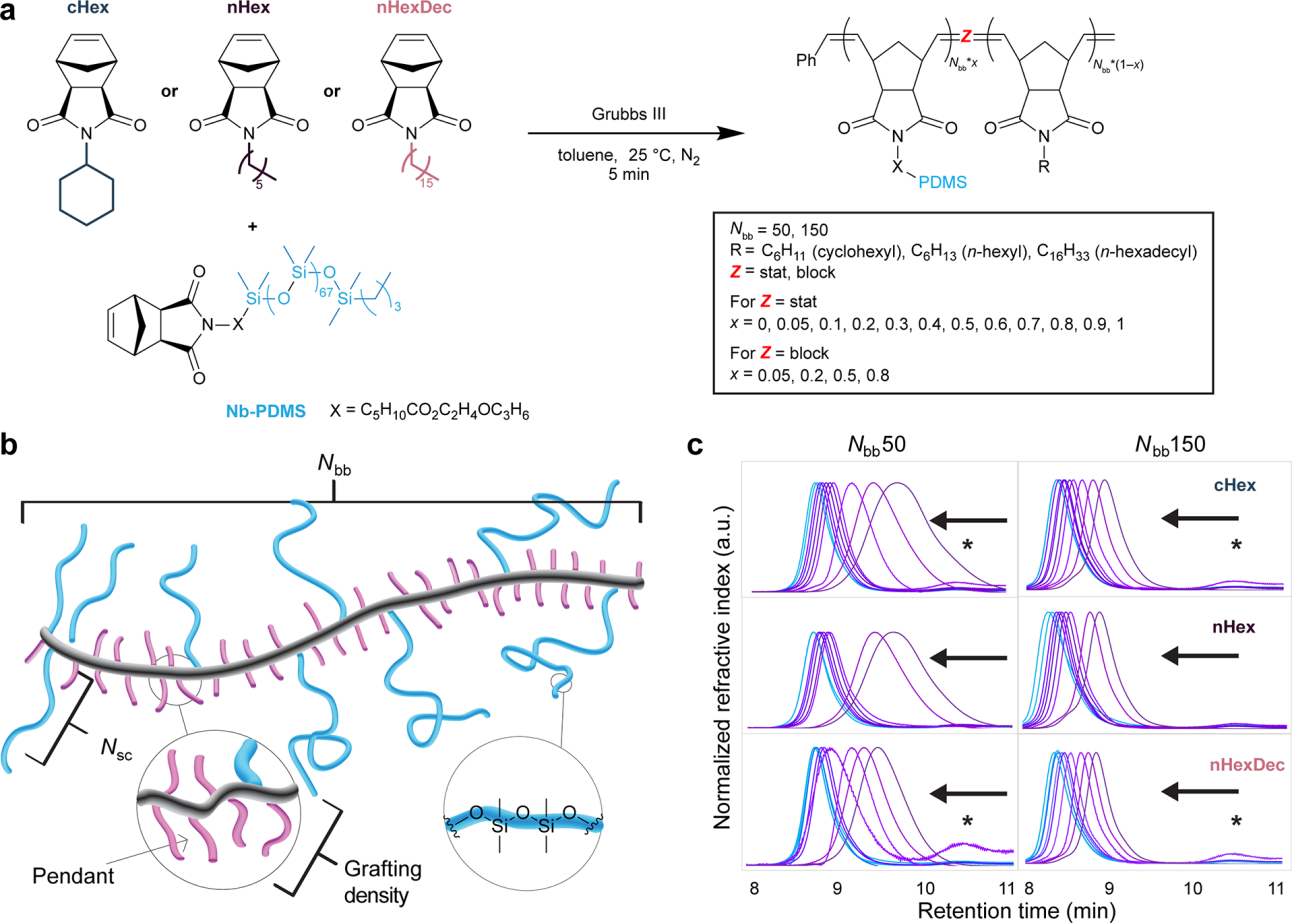
Synthesis of a library of graft copolymers. (a) Graft copolymers comprising polynorbornene backbones, PDMS arms (**Nb-PDMS**), and small-molecule comonomer pendants (**cHex**, **nHex**, or **nHexDec**) are prepared by Ru-initiated ROMP using the Grubbs 3rd-generation bis-pyridyl complex (“Grubbs III”). See Supporting Information for specific calculation of how these variables yield 92 polymer structures. (b) Schematic representation of the graft copolymer architecture, emphasizing the role of pendant groups. (c) Size exclusion chromatography refractive index traces of *statistical* graft copolymers. Arrows indicate direction of increasing grafting density. *nHex-containing polymers at grafting density = 0.2 are isorefractive with chloroform and could not be detected. **cHex** and **nHexDec** copolymers at grafting density = 0.2 are nearly isorefractive with chloroform, resulting in an exaggerated residual macromonomer signal despite >99.9% conversion by ^1^H NMR spectroscopy (retention time ∼10.5 min).

## Systematic Synthesis of a Library of Graft Copolymer Silicones

PDMS-based macromonomer **Nb-PDMS** and small-molecule comonomers with aliphatic pendants **cHex**, **nHex**, and **nHexDec** were each prepared in one step from the appropriate *exo*-norbornene electrophile and commercially available hydroxyl-terminated PDMS (5 kDa), hexylamine, cyclohexylamine, or hexadecylamine, respectively (Figure 1a). Leveraging the robust nature of Ru-initiated ROMP,^46^ a library of 92 (co)polymer compositions was prepared from these 4 (macro)monomers in parallel on the 100–500 mg scale, with each ROMP reaction taking ∼5 min to reach completion (Figures 1a,b and S1–S11 and Tables S1–S4). To demonstrate scalability of specific members of this library, several samples that displayed interesting mechanical properties were produced on the decagram scale with no purification steps required following polymerization. Comonomers **cHex**, **nHex**, and **nHexDec** differ by the identity of their aliphatic pendant groups, which vary in size, conformational freedom, and self-interactions. For statistical copolymers, graft densities of 0, 0.05, 0.1, 0.2, 0.3, 0.4, 0.5, 0.6, 0.7, 0.8, 0.9, and 1.0 were explored, the first and last of which represent homopolymers of the small-molecule and PDMS (macro)monomers, respectively. Diblock copolymer analogues were synthesized at grafting densities of 0.05, 0.2, 0.5, and 0.8. Two backbone degrees of polymerization (*N*_*bb*_), 50 and 150, for each composition were prepared to enable investigation of the impact of *N*_*bb*_ while maintaining a manageable library size. For clarity, all copolymers are referred to by a ***sequence*****-pendant-*****N***_***bb***_**-grafting density** formalism. For example, a statistical copolymer with a cyclohexyl pendant, *N*_*bb*_ of 50, and grafting density of 0.4 is referred to as ***stat*****-cHex-50-0.4**.

This library synthesis approach allows for parallel tuning of grafting density, sequence, pendant composition, and *N*_*bb*_ (Figure 1b), all of which play key roles in determining polymer conformations, self-interactions, and ultimately self-assembly and bulk properties. Specifically, grafting density influences backbone and side-chain flexibilities^47^ and the volume fraction (*f*) of components. Statistical or block sequence influences domain spacing (d*), chain conformations, and entanglement molecular weight.^12^*N*_*bb*_ determines number-average molar mass (*M*_n_) and additionally influences whether or not backbone entanglements can occur. Lastly, the comonomer pendant groups influence copolymer backbone flexibility and self-interactions. We note that the PDMS side chain degree of polymerization (*N*_sc_) was not varied in this study to limit the total number of samples and focus on the effects of the comonomer pendant groups. Moreover, the effects of graft copolymer side chain length have been investigated thoroughly elsewhere.^12,20,48−51^ Normalized size exclusion chromatography (SEC) traces for all statistical copolymers are shown in Figure 1c and block samples are shown in Figures S12–S17. Copolymerization kinetics experiments^1,52^ revealed that each of the three small-molecule comonomers copolymerize with **Nb-PDMS** with similar efficiency, yielding copolymers with slightly gradient monomer distributions (*r*_1_ < 1 < *r*_2_ where the small molecule is monomer 1 and **NbPDMS** is monomer 2, with *r*_1_ × *r*_2_ = 1.16, 1.52, and 1.90 for **cHex**, **nHex**, and **nHexDec**, respectively) (Figures S18–S26 and Table S5).

## Room-Temperature Self-Assembly of Silicone-Based Graft Copolymers

**Figure 2:**
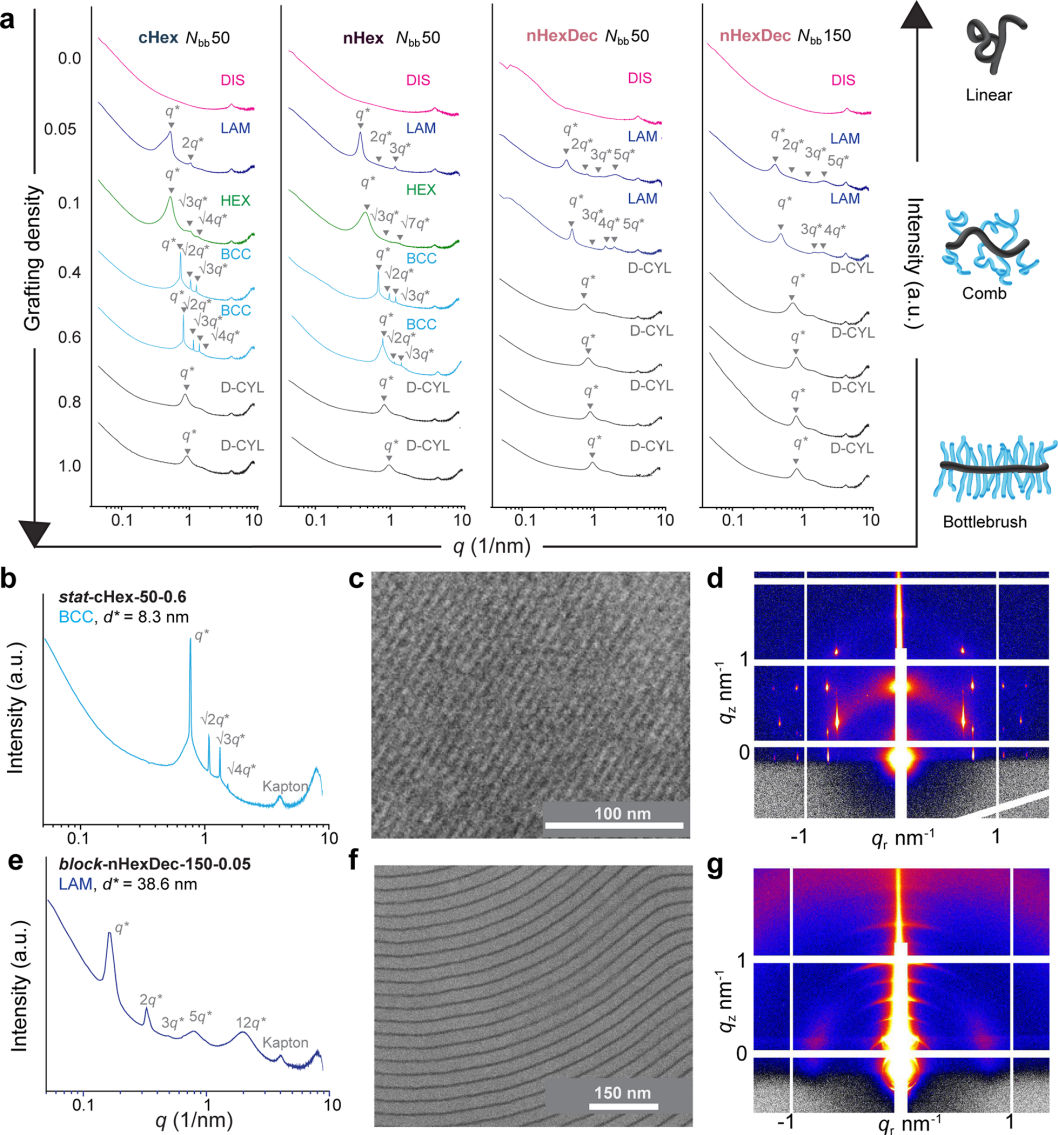
Self-assembly and surface effects of pendant-modified statistical graft copolymers. (a) 1D SAXS curves of statistical copolymers. DIS = disordered, LAM = lamellar, HEX = hexagonally packed cylinders, BCC = body-centered cubic, D-CYL = long-range-disordered worm-like cylinders. (b) 1D SAXS of ***stat*****-cHex-50-0.6**. (c) TEM image of ***stat*****-cHex-50-0.6** shows 110 lattice fringes of BCC morphology. (d) GISAXS of ***stat*****-cHex-50-0.6** shows highly ordered anisotropic surface assembly; 110-oriented BCC lattice. (e) 1D SAXS of ***block*****-nHexDec-150-0.05**. (f) Cryo-microtome TEM image of ***block*****-nHexDec-150-0.05**. (g) GISAXS of ***block*****-nHexDec-150-0.05** shows highly ordered lamellae in thin film parallel to surface.

**Figure 3:**
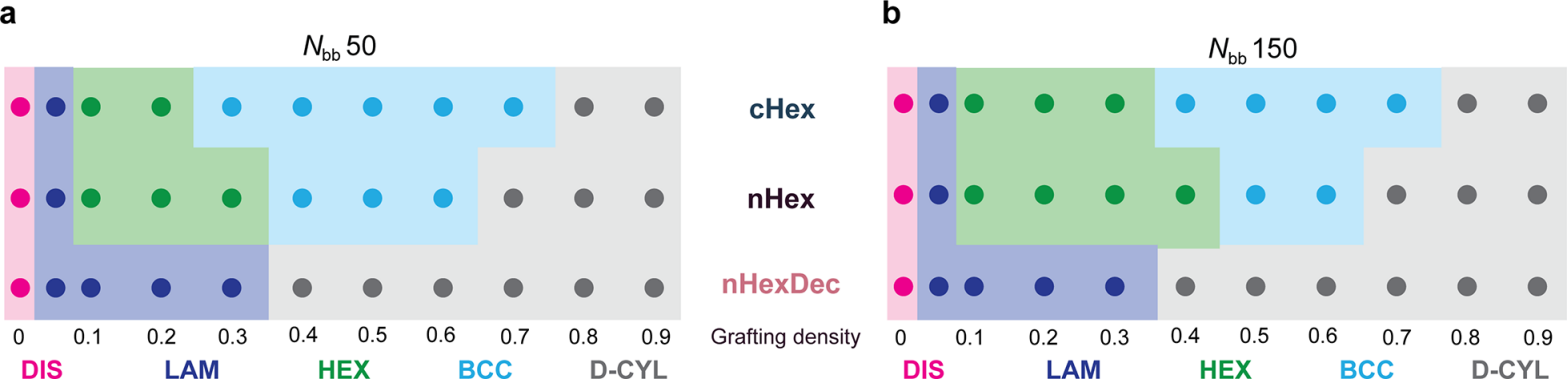
Phase map of statistical copolymers and PNB homopolymers as a function of grafting density and comonomer. (a) *N*_*bb*_ 50 and (b) *N*_*bb*_ 150.

Bulk samples of each polymer were prepared by drop-casting from toluene solution and drying under vacuum at room temperature. The resulting materials were subjected to small-angle X-ray scattering (SAXS, Figure 2a,b,e, Tables S1–S4, and Figures S27–S48) and, in some cases, transmission electron microscopy (TEM, Figures 2c,f and S49–S54). In many instances, highly ordered morphologies, driven by phase separation of PDMS and the polynorbornene/comonomer backbone, were observed. Progressing from a grafting density of 0.9 to 0.05 (*f*_*PDMS-MM*_ from ∼0.995 to ∼0.53 for **cHex** and **nHex** or 0.42 for **nHexDec** copolymers) and a concurrent transition from bottlebrush, to comb, to linear architecture (Figure 2a) provided an array of morphologies including disordered (DIS), lamellar (LAM), hexagonally close packed cylinder (HEX), body-centered cubic (BCC) packed spherical, and long-range-disordered worm-like cylinder (D-CYL, with d* values that correspond to graft copolymer backbone-to-backbone distances^53−55^) for statistical samples as suggested by SAXS (Figure 3a,b). We surmise that the narrower BCC region in the *N*_*bb*_ 150 samples is due to greater backbone length of these polymers, which limits their ability to coil around small BCC nodes. Nevertheless, while LAM, HEX, and D-CYL morphologies of bottlebrush and graft copolymers are well-documented, highly ordered BCC morphologies are less common for densely grafted polymers.^39^ Notably, BCC morphologies were not accessible to any of the diblock copolymers (Figures S35, S36, S41, and S42) nor were they observed in the **nHexDec** series (Figures S43–S48), suggesting that backbone collapse, which is enabled by reductions in grafting density and/or with smaller comonomer pendants, is necessary to achieve this morphology. Similarly, we observe a wider BCC region for **cHex** versus **nHex**, suggesting a superior ability of polymers with shorter (and better self-packing) pendant groups to more easily adopt highly curved conformations (Figure 3a,b).

Copolymers containing **cHex** and **nHex** displayed BCC or HEX morphologies at grafting densities of 0.7–0.1, while LAM morphologies were observed for all samples at grafting density of 0.05. Notably, the formation of LAM in the **nHexDec** series occurs at *f*_*PDMS-MM*_ as high as 0.86, which is attributed to the steric bulk of the long aliphatic comonomer chains and the concomitant rigidification of the polynorbornene backbone. Lastly, the morphologies of the statistical copolymers are insensitive to *N*_*bb*_, which agrees well with similar results observed for statistical and Janus bottlebrush multiblock copolymers (Figure 2a).^3,4,38,56^

Interestingly, samples with highly ordered BCC morphologies based on SAXS (Figure 2b) showed line patterns when analyzed by TEM (Figure 2c). We posited that this observation could be due to a change in the material morphology due to thin-film confinement, or it could be the result of imaging lattice fringes of a BCC morphology. In the latter case, these TEM images should represent a 110 projection of the BCC lattice, given that the line spacing in Figure 2c (d* = 8.0 nm) matches well with the 110 interplanar spacing calculated from SAXS (d*_110_ = 8.3 nm, Figure 2b). To address this question, a select BCC sample, ***stat*****-cHex-50-0.6**, was studied with grazing-incidence small-angle X-ray scattering, a low-incidence-angle technique typically used for studying the assembly of thin films at surfaces (GISAXS, Figure 2d, Figures S55–S60, indexed in Figure S61), which revealed significant surface anisotropy and confirmed the BCC morphology with a lattice parameter *a* = 12.3 nm as well as its preferential (110) orientation of the thin film (Figures S61 and S62). Samples with LAM morphology based on SAXS (e.g., Figure 2e) required microtoming prior to TEM imaging to visualize the cross sections of the lamellae (Figure 2f) due to the tendency of the sample to form wetting layers parallel to the air and substrate interfaces due to the low surface energy of PDMS. GISAXS of one such sample ***block*****-nHexDec-150-0.05** showed well-ordered lamellae with a period of 37.0 nm (in good agreement with the d* = 38.6 nm as calculated from SAXS in Figure 2e) and confirmed its predominantly in-plane orientation to the surface of deposition (Figures 2g, S63, and S64).

**Figure 4:**
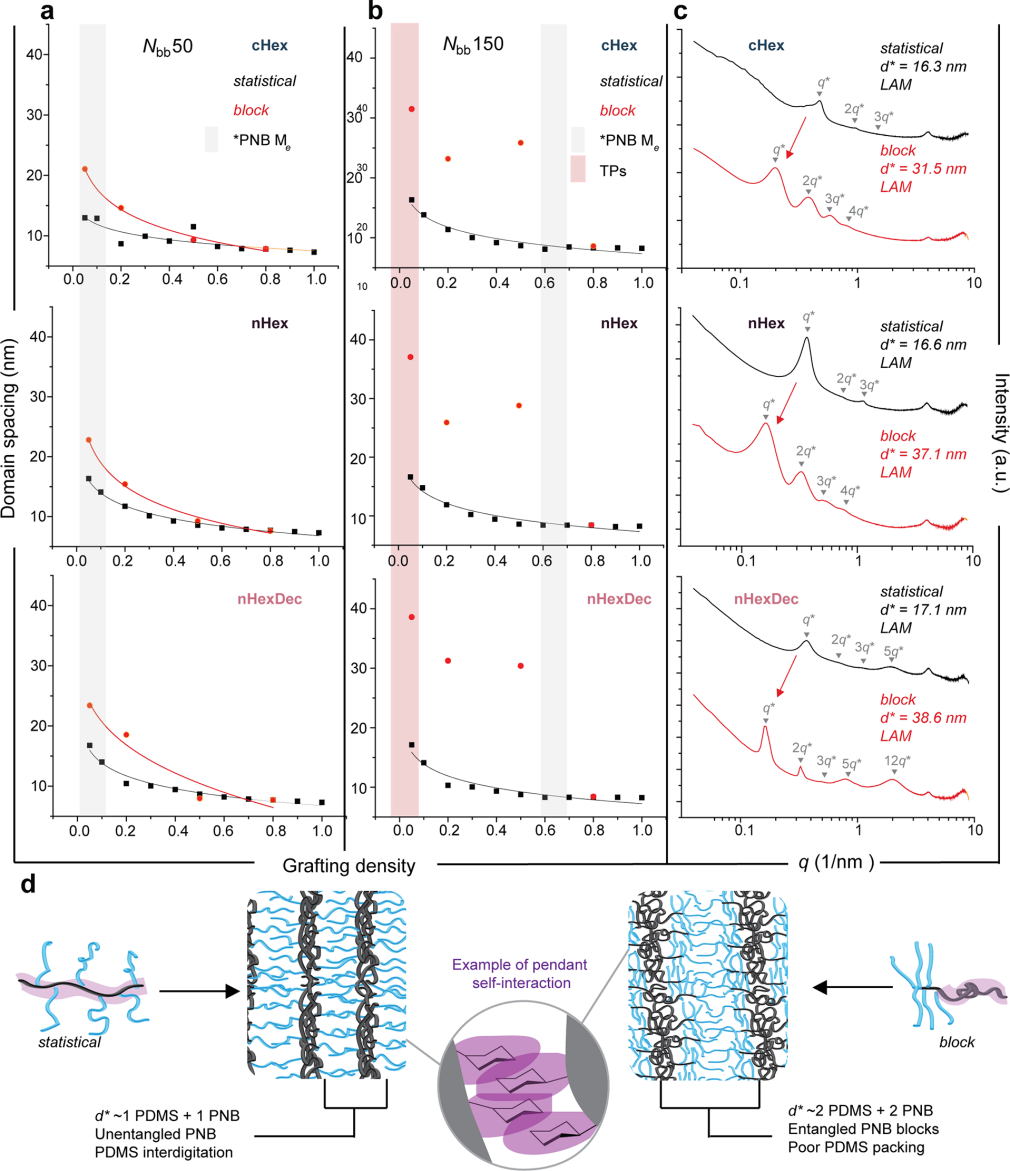
Influence of molecular architecture on domain spacing. (a,b) Domain spacing vs grafting density for *N*_bb_ = 50 and 150 copolymers, separated by small-molecule comonomer and overlaid by architecture. Black lines are given by a best-fit power law function. Gray rectangles denote approximate entanglement degree of polymerization of linear polynorbornene homopolymer. Red rectangle highlights six TP samples analyzed further. (c) 1D SAXS curves of six TP-forming samples (highlighted by the red rectangle in (b), *N*_bb_ = 150, grafting density = 0.05) showing approximate doubling of domain spacing of *block* vs *stat* samples. (d) Illustration of molecular process underlying doubling of domain spacing in blocky samples above entanglement threshold. *PNB *M_e_* is estimated based on measurements conducted by Boyle et al.^57^ on homopolymers of *n*-hexyl functionalized polynorbornene imides.

To correlate the composition of each sample with morphological periodicity, domain spacing (d*, nm, calculated as 2π/q* where q* is the principal scattering peak in nm^–1^ measured by SAXS) was plotted against grafting density for each side chain sequence (statistical versus block), comonomer identity, and *N*_*bb*_ value (Figure 4a,b). As expected,^3,5,56,58^ while *N*_*bb*_ has little impact on d* for the statistical copolymers, it has a large effect for the diblock copolymers (Figure 4a–c) due to their tendency to assemble in side-to-side versus head-to-head orientations (Figure 4d), respectively. The qualitatively different relationship between d* and grafting density for diblock copolymers of *N*_*bb*_ = 50 versus 150 can be attributed to the presence of entanglements in the PNB blocks of the latter when grafting density is below ∼0.6 (*Note*: the entanglement degree of polymerization of linear polynorbornene with *n*-hexyl pendants was reported^57^ to be ∼45). Despite the considerable variations in morphology as a function of comonomer composition, the trends in d* vs grafting density were similar for each comonomer, demonstrating that this simple copolymerization approach can provide facile access to materials with diverse morphologies and independently tunable d* values from a simple set of (macro)monomer precursors.

## Mechanical Properties of Graft Copolymer Silicones

**Figure 5:**
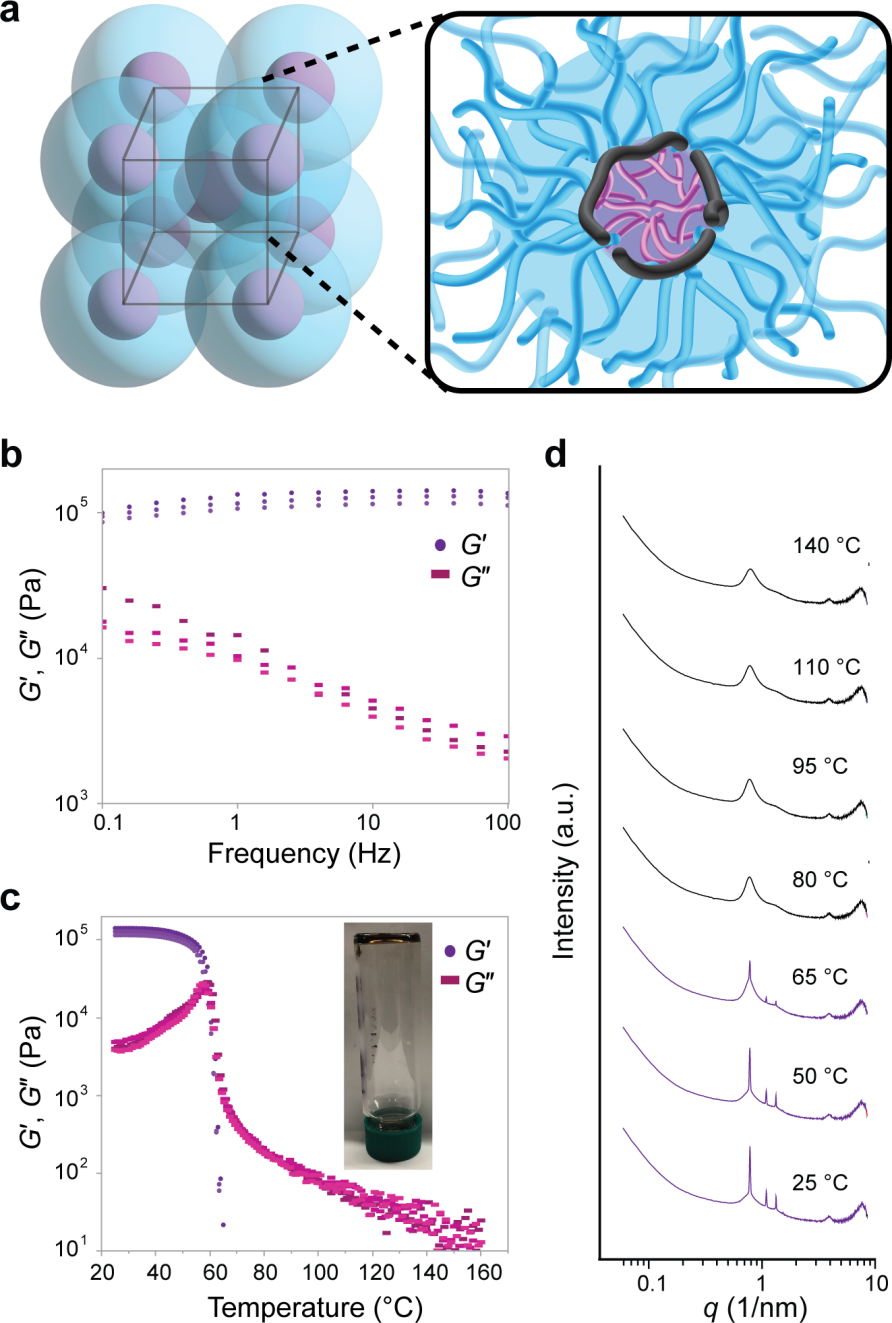
Rheological characterization of representative BCC sample, ***stat*-nHex-150-0.6**. (a) Schematic depicting polymer conformation in BCC morphology. PDMS chains are expected to interdigitate and form a continuous phase which enables positional order but not orientational order within the lattice. (b) Frequency sweep rheology. (c) Temperature sweep rheology highlights sharp transition from viscoelastic solid to liquid. Inset: inverted vial showing viscoelastic solid behavior of sample. (d) Variable-temperature SAXS reveals an order–disorder transition temperature between 65 and 80 °C, in agreement with the transition noted in (b).

Next, we sought to characterize the mechanical properties of select members of our graft copolymer library. First, we focused on samples that displayed the BCC morphology, which behaved as soft, viscoelastic solids despite their very high PDMS volume fractions and unentangled nature (*f*_*PDMS-MM*_ of ∼0.98). Small-angle oscillatory shear rheology frequency (Figure 5b) and temperature (Figure 5c) sweeps of BCC sample ***stat*-nHex-150-0.6** demonstrated a broad viscoelastic regime with a plateau storage modulus of 130 ± 10 kPa. Additionally, another BCC sample—***stat*-cHex-150-0.6**—displayed a 4 order-of-magnitude greater *G*′ than D-CYL sample ***stat*-cHex-150-0.7**, despite only differing in *f*_PDMS-MM_ by 0.01 (Figure S65). We propose that the structures of the BCC samples most likely comprise the comonomer pendant groups and polynorbornene backbones closely packed in BCC cores embedded in an unbridged, rubbery PDMS matrix, forming plastic crystals where the lattice energy of the assembly provides physical cross-linking and a fixed positional order of the BCC nodes.^59−62^ The PDMS chains that comprise the matrix surrounding the BCC nodes are above their *T*_*g*_ and un-cross-linked, and therefore, they are free to rotate about their nodes.

A rapid transition from viscoelastic solid to liquid is observed at 75 ± 1 °C, which is consistent with the order-to-disorder transition (*T*_ODT_) observed by variable-temperature SAXS (Figure 5d). This transition underlies a shift from BCC to D-CYL, and though we note that the D-CYL arrangement does have short-range order, it is the loss of long-range order, as reflected by a morphology change and peak broadening, that causes us to denote this as an order-to-disorder transition and not an order-to-order transition. The *T*_*ODT*_s of the BCC samples prepared in this study ranged over ∼20 °C as a function of the graft copolymer composition, offering a strategy for tuning the processing conditions of these materials (Figures S66–S70). Interestingly, samples with BCC morphologies were not observed to be thermally reversible on slow or rapid cooling.

**Figure 6:**
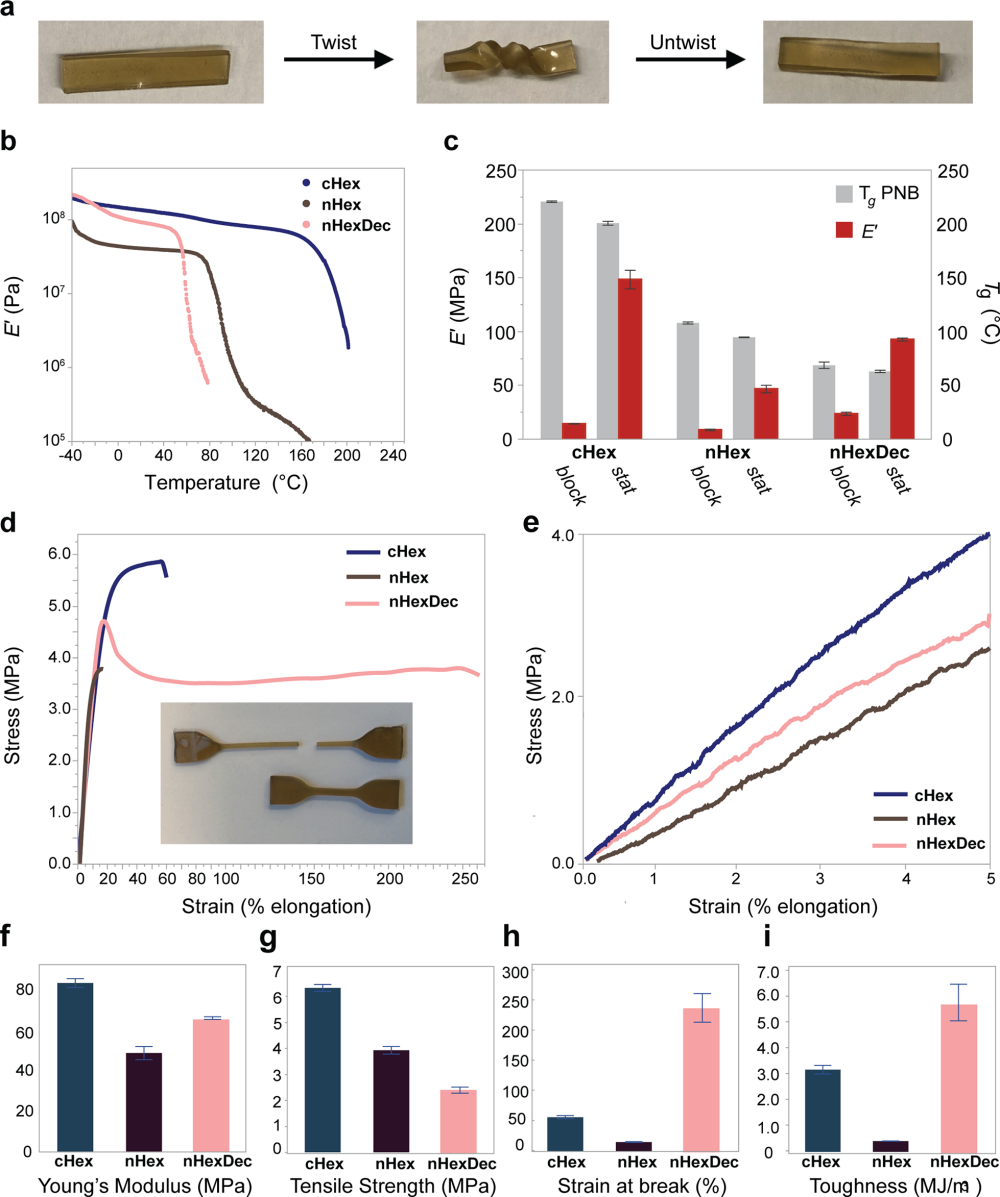
Thermomechanical performance of lamellar TPs. (a) Samples can be deformed and hold their shape after deformation (sample shown is **stat-nHexDec-150-0.05**). (b) Stacked *E*′ plots from DMA temperature sweeps of *stat*-150-0.05 **cHex**, **nHex**, and **nHexDec** samples demonstrate pendant-sensitive modulus and glass transition temperature. (c) Tabulated *E*′ (at 298 K) and *T*_g,PNB_ (from maximum in tan(δ)) as measured by DMA of the same samples. (d) Overlaid representative stress–strain curves of the same samples. Inset: ***stat*-nHexDec-150-0.05** tensile samples before (bottom) and after (top) tensile testing to failure. (e) Close-up of first 5% of strain during tensile testing used to obtain relative Young’s moduli. (f–i) Mechanical properties as obtained from stress–strain curves in triplicate, (f) Young’s modulus (MPa), (g) ultimate tensile strength (MPa), (h) strain at break (%), (i) toughness (MJ m^–3^). Error bars represent standard error. *Note*: All samples analyzed by DMA and tensile testing possess a LAM morphology.

We noticed that LAM samples with low grafting densities (∼0.05) formed unusually robust, albeit malleable, transparent solids (Figure 6a), warranting further investigation. Samples of 5–10 g of ***stat*****-150-0.05** and ***block-*****150-0.05** copolymers with each pendant group were synthesized following similar procedures used for the library synthesis and were analyzed by dynamic mechanical analysis (DMA) and tensile testing.

DMA revealed that these samples behaved as unentangled thermoplastics, as each of them adopted a viscoelastic-liquid nature once heated above their *T*_*g*_. They displayed a wide range of properties including room-temperature storage moduli (*E*′) up to ∼150 MPa (Figures 6b,c, S71, and S72), which are similar to cartilage, tendons, and muscle tissues, greater than commercial and exploratory cross-linked silicones,^44,63−65^ and yet softer than typical glassy thermoplastics, which are typically >1 GPa.^66−68^ A strong dependence of *E*′ on side chain sequence was observed, with the three statistical copolymers displaying much higher values than their block copolymer counterparts. For example, ***stat*-cHex-150-0.05** has a ∼15-fold greater *E*′ than ***block*-cHex-150-0.05** despite their isomeric compositions (Figure 6b). Given that these samples are not chemically cross-linked and are expected to have few entanglements (at most one entanglement per polynorbornene block in the case of blocky samples), their disparate mechanical properties can be explained by the much greater density of high-energy phase boundaries in the statistical copolymer, as reflected by their lower d*.

Notably, the thermal transitions of these materials were not sequence dependent, changing by only ∼10% between statistical and block samples, and were not related to *T*_*ODT*_s (Figures S73–S89 and Table S6). Instead, they were dominated by the glass transition temperatures of the small-molecule norbornene homopolymers (*T_g,PNB_*) with **cHex** > **nHex** > **nHexDec** (201 ± 4, 95 ± 1, 64 ± 1 °C, respectively. The comonomers also had a major effect on *E*′, decreasing from ∼150 to ∼100 to ∼50 MPa from **cHex** to **nHexDec** to **nHex**, respectively.

Universal tensile testing was conducted in triplicate on the three statistical graft copolymer samples with different comonomer pendant groups (Figure 6d–i) (*Note*: due to their lack of entanglement and limited PDMS interdigitation, Figure 4d, the block copolymer samples were too brittle to acquire meaningful tensile data). Each sample demonstrated thermoplastic behavior, as they did not show any signs of reversible deformation at modest or high strain and were observed to either yield plastically or fail at low strain. The Young’s moduli (Figure 6f) trended with the storage moduli obtained by DMA. The comonomer pendant composition had major impacts on the ultimate tensile strength (Figure 6g), strain at break (Figure 6h), and toughness (Figure 6i, approximated by the area underneath the stress–strain curves). For example, strain at break ranged from 230 ± 54 to 57 ± 5 to only 16 ± 1% for **nHexDec**, **cHex**, and **nHex** samples, respectively. We attest the superior strain at break of the **nHexDec** samples to the ability of flexible **nHexDec** pendants to most effectively fill space and therefore reduce the likelihood of crack propagation during applied strain. Meanwhile, **nHex** pendants do not impart comparable strain at break to their respective materials as a result of the significantly smaller surface area and intermolecular interaction strength of these shorter pendants. Tensile strength ranged from 2.4 ± 0.2 (**nHexDec**) to 6.3 ± 0.2 (**cHex**) MPa, on par with commercial silicone elastomers such as Sylgard 184 (ultimate tensile strength ≈ 6.8 MPa).^44^ Similarly, toughness varied from ∼6 to ∼3 to ∼0.5 MJ·m^–3^ across these samples. Altogether, these differences are attributed to a combination of the pendant group self-interaction strength, space-filling, and ability to undergo structural rearrangements during extension.

Lastly, we expected that the TPs could be reprocessed by conventional means, and so, both thermal and solvent reprocessing were pursued. To attempt thermal reprocessing, ***stat*-nHexDec-150-0.05** was heated at 100 °C under 3 tons of applied force for 10 min, yielding an apparently cohesive material (see Supporting Information and Figure S90); however, the resultant material was susceptible to rapid crack propagation, likely due to inhomogeneities caused by the inability of neighboring domains of previously separated pieces to effectively self-assemble, as the sample was above its *T*_*g*_ but below its *T*_*ODT*_. Thermogravimetric analysis suggested that the samples should be thermally stable up to approximately 400 °C under nitrogen, so we anticipate that no destructive chemical change occurred during the compression molding process (Figure S91). Nonetheless, ***stat*-nHexDec-150-0.05** samples could be dissolved in toluene and recast under vacuum at room temperature into dogbones. These dogbones were resubjected to tensile testing to demonstrate maintenance of mechanical properties. This process was repeated three times with negligible impact on tensile properties, showing that the samples are solvent reprocessable to their original state (Figure S92).

## Summary and Outlook

Herein, we investigated the impact of comonomer composition, specifically the identity of comonomer pendant group, on graft (co)polymer-based silicones using a systematic library synthesis approach. Using only one PDMS macromonomer and three comonomers that differed by their aliphatic pendants, a library of 92 (co)polymers with varied sequences, backbone lengths, grafting densities, and comonomer compositions was synthesized. Characterization of this library confirmed many well-established trends in graft copolymer assembly (across well-studied parameters such as grafting density or sequence) but also revealed dramatic variations in material properties and accessible morphologies that correlated with comonomer pendant group composition and sequence, offering a new and simple strategy to target bespoke material properties. Moreover, members of this library displayed properties that span the gamut of traditional silicones, with certain features surpassing those of commercial systems; for example, a new class of transparent, reprocessable silicone thermoplastics with stiffness and toughness that surpass unfilled, cross-linked silicones as well as classical rubbers (e.g., ABS/SBS formulations) was discovered. Looking forward, we note that while the impacts of comonomers in this system are undeniable, the precise molecular mechanisms that translate from pendant composition to microphase morphology to bulk properties remain to be elucidated. In the future, this general approach should be applicable to a range of chemical systems in addition to silicones and perhaps could be coupled to high-throughput experimentation and data science techniques to achieve rapid discovery/optimization of new materials.

## Data Availability

All data supporting the findings of this study are available within the article and the Supporting Information and/or from the corresponding author upon reasonable request.

## Supporting Information Available

Detailed experimental procedures and methodologies, calculation details, and additional data. Materials and methods, small-molecule and polymer synthesis, polymer characterization, homo- and copolymerization kinetic analyses, complete small-angle X-ray scattering of all samples, transmission electron microscopy of select samples, grazing-incidence small-angle X-ray scattering of select samples, rheological characterization of select samples, variable-temperature small-angle X-ray scattering of select samples including determination of order–disorder transition temperatures, attempts at direct reprocessing of select samples, thermogravimetric analysis of select sample, complete dynamic mechanical analysis temperature sweeps of select samples, references (PDF)
