# Supplementary material for: Pendant Group Modifications Provide Graft Copolymer Silicones with Exceptionally Broad Thermomechanical Properties
Source: ACS Cent Sci. 2022 Dec 23;9(1):36–47. doi: 10.1021/acscentsci.2c01246 (PMC9881205; doi:10.1021/acscentsci.2c01246)
Supplement: Supplementary file 1 — oc2c01246_si_001.pdf [file oc2c01246_si_001.pdf]

Supporting Information for:

Pendant Group Modifications Provide Graft Copolymer Silicones with Exceptionally Broad Thermomechanical Properties

Keith E. L. Husted,[1] Abraham Herzog-Arbeitman,[1] Denise Kleinschmidt,[1] Wenxu Zhang,[1] Zehao Sun,[2] Alyssa J. Fielitz,[3] An N. Le,[4] Mingjiang Zhong,[3] Jeremiah A. Johnson*[1]

1. Department of Chemistry, Massachusetts Institute of Technology, Cambridge, MA 02139, United States
2. Department of Materials Science and Engineering, Massachusetts Institute of Technology, Cambridge, MA 02139, United States
3. Dow Core R&D, Analytical Sciences, The Dow Chemical Company, Midland, Michigan 48640, USA
4. Department of Chemical and Environmental Engineering, Yale University, New Haven, CT 06520, United States

*****Corresponding author e-mail: jaj2109@mit.edu

Table of Contents:

Materials and methods	S2
Synthesis	S2-S5
Characterization	S5-S29
Kinetics	S30-S38
Small-angle X-ray scattering	S39-S60
Transmission electron microscopy	S61-S66
Grazing incidence small-angle X-ray scattering	S67-S76
Rheological characterization	S77
Variable-temperature small-angle X-ray scattering	S78-S82, S85-S102
Reprocessing	S103, S105
Thermogravimetric Analysis	S104
Dynamic mechanical analysis	S83, S84
References	S107

Materials and Methodology

Materials

Solvents and amines were purchased from Sigma Aldrich and used as received. All reagents were used as received, without further purification. Monocarbinol terminated PDMS (M_n = 5,000 Da) was purchased from Gelest Inc. *cis*-5-Norbornene-*exo*-2,3-dicarboxylic anhydride was purchased from Oakwood Chemical. SiliaMetS DMT were purchased from Silicycle. Grubbs 3rd generation catalyst (G3),[1] **cHex**,[2] **nHex**,[3] **nHexDec** and **NB-PDMS**,[4] were prepared following reported procedures.

Synthesis

Synthesis of ***cHex***

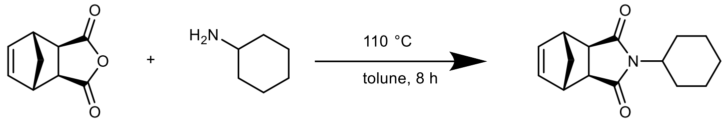

cis-5-Norbornene-*exo*-2,3-dicarboxylic anhydride (6.000 g, 36.55 mmol, 1.000 eq) and cyclohexylamine (4.264 mL, 3.6970 g, 37.28 mmol, 1.020 eq), were added to a 1 L roundbottom flask with toluene (500 mL). After heating under reflux for 8 h, the mixture was allowed to cool then dried under rotary evaporator, dissolved in ethyl acetate (500 mL), and washed with HCl (3 x 500 mL, pH 3), brine (1 x 500 mL), dried over sodium sulfate and then concentrated to approximately 25 mL under vacuum to yield a viscous solution. Crystals were allowed to form over 2 days, filtered through qualitative filter paper, washed with cold hexanes and dried under vacuum, yielding white crystalline solids. (9.7962 g, **89%**).

^{13}C NMR (101 MHz, Chloroform-*d*) δ 178.26, 137.87, 51.61, 45.39, 42.55, 28.75, 25.81, 25.03.
^{1}H NMR (400 MHz, Chloroform-*d*) δ 6.29 (t, *J* = 1.9 Hz, 2H), 3.95 (tt, *J* = 12.4, 3.9 Hz, 1H), 3.54 – 3.06 (m, 2H), 2.62 (d, *J* = 1.4 Hz, 2H), 2.15 (qd, *J* = 12.5, 3.5 Hz, 2H), 1.83 (dt, *J* = 11.3, 2.7 Hz, 2H), 1.66 (dt, *J* = 13.6, 3.0 Hz, 1H), 1.49 (dt, *J* = 9.9, 1.6 Hz, 1H), 1.43 – 1.06 (m, 5H).

HRMS (DART+): Calculated for $[M+H]^+$: 246.1490, found 246.1524.

Synthesis of ***nHex***

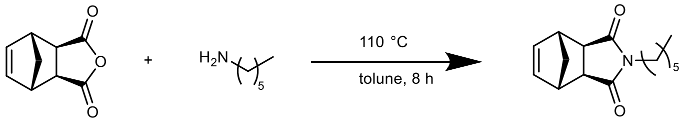

cis-5-Norbornene-*exo*-2,3-dicarboxylic anhydride (6.0694 g, 36.97 mmol, 1.000 eq) and hexylamine (4.988 mL, 3.841 g, 37.95 mmol, 1.027 eq) were added to a 1 L roundbottom flask with toluene (500 mL). After heating under reflux for 8 h, the mixture was allowed to cool then dried under rotary evaporator, dissolved in ethyl acetate (500 mL), and washed with HCl (3 x 500 mL, pH 3), brine (1 x 500 mL), dried over sodium sulfate and then dried under vacuum to yield a colorless oil (8.5436 g, **94%**).

^{13}C NMR (101 MHz, Chloroform-*d*) δ 177.75, 137.68, 47.62, 45.01, 42.56, 38.51, 31.15, 27.56, 26.45, 22.31, 13.84.
^{1}H NMR (400 MHz, Chloroform-*d*) δ 6.13 (s, 1H), 3.37 – 3.18 (m, 2H), 3.09 (s, 1H), 2.51 (d, *J* = 1.5 Hz, 2H), 1.59 – 1.24 (m, 3H), 1.23 – 0.99 (m, 7H), 0.89 – 0.48 (m, 3H).

HRMS (DART+): Calculated for $[M+H]^+$: 248.1651, found 248.1671.

Synthesis of ***nHexDec***

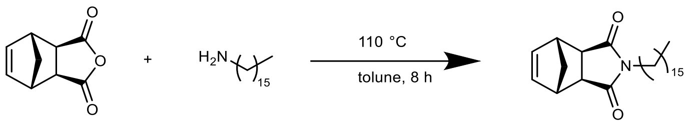

cis-5-Norbornene-*exo*-2,3-dicarboxylic anhydride (6.2380 g, 38.00 mmol, 1.000 eq), and hexadecylamine (9.3900 g, 38.89 mmol, 1.023 eq), were added to a 1 L roundbottom flask with toluene (500 mL). After heating under reflux for 8h, the mixture was allowed to cool, then dried under rotary evaporator, dissolved in ethyl acetate (500 mL), and washed with HCl (3 x 500 mL, pH 3), brine (1 x 500 mL), dried over sodium sulfate and then concentrated to approximately 25 mL under vacuum to yield a viscous solution. Crystals were allowed to form over 2 days, filtered through qualitative filter paper, washed with cold hexanes and dried under vacuum, yielding white crystalline solids. (14.2450 g, **90%**).

^{13}C NMR (101 MHz, $CDCl_3$) δ 178.30, 138.03, 77.52, 77.40, 77.20, 76.88, 47.99, 45.36, 42.90, 38.97, 32.12, 29.88, 29.85, 29.81, 29.74, 29.65, 29.56, 29.34, 27.98, 27.17, 22.89, 14.32.
^{1}H NMR (400 MHz, $CDCl_3$) δ 6.28 (s, 1H), 3.61 – 3.37 (m, 2H), 3.27 (q, *J* = 1.7 Hz, 2H), 2.67 (d, *J* = 1.4 Hz, 2H), 1.72 – 1.42 (m, 3H), 1.41 – 1.13 (m, 23H), 0.88 (t, *J* = 6.7 Hz, 2H).

HRMS (DART+): Calculated for $[M+H]^+$: 388.3216, found 388.3220.

Synthesis of ***Nb-COOH***

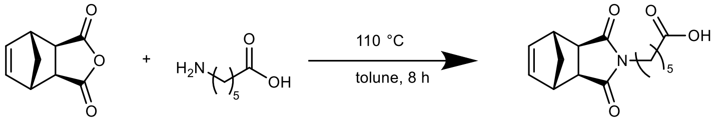

cis-5-Norbornene-*exo*-2,3-dicarboxylic anhydride (12.476 g, 76.00 mmol, 1.000 eq) and 6-amino hexanoic acid (10.137 g, 77.28mmol, 1.017 eq) were added to a 1 L roundbottom flask with toluene (500 mL). After heating under reflux for 8 h, the mixture was allowed to cool, then dried under rotary evaporator, dissolved in ethyl acetate (500 mL), and washed with HCl (3 x 500 mL, pH 3), brine (1 x 500 mL), dried over sodium sulfate, and concentrated to approximately 25 mL under vacuum to yield a viscous solution. Crystals were allowed to form over 2 days, filtered through qualitative filter paper, washed with cold hexanes and dried under vacuum, yielding white crystalline solids in 20.5 g yield (**93%**).

^{13}C NMR (126 MHz, $CDCl_3$) δ 178.72, 178.13, 137.87, 137.84, 137.82, 77.28, 77.23, 77.03, 76.77, 47.82, 47.79, 45.21, 45.17, 42.73, 38.42, 33.62, 27.42, 26.35, 24.14.
^{1}H NMR (500 MHz, $CDCl_3$) δ 6.30 (t, *J* = 1.9 Hz, 1H), 3.65 – 3.38 (m, 1H), 3.29 (t, *J* = 1.8 Hz, 1H), 2.69 (d, *J* = 1.4 Hz, 1H), 2.37 (t, *J* = 7.4 Hz, 1H), 1.68 (p, *J* = 7.5 Hz, 1H), 1.64 – 1.56 (m, 1H), 1.53 (dt, *J* = 9.9, 1.6 Hz, 0H), 1.43 – 1.33 (m, 1H), 1.23 (ddt, *J* = 9.8, 1.5, 0.8 Hz, 0H).

HRMS (DART+): Calculated for $[M+H]^+$: 278.1392, found 278.1411.

Synthesis of ***Nb-PDMS***

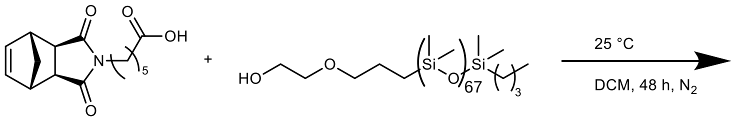

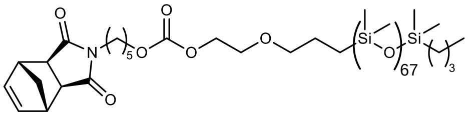

NB-COOH (15 g, 54 mmol, 2.0 eq), EDC hydrochloride (10.38 g, 54.15 mmol, 2.006 eq) and dimethyl aminopyridine (0.660 g, 5.40 mmol, 0.200 eq) were added to a dry 2 L roundbottom flask, and purged with nitrogen. Anhydrous HPLC grade DCM (1.5 L) was added and the mixture was allowed to stir for 15 minutes.

5,000 Da monocarbinol PDMS (135 g, 27.0 mmol, 1.00 eq) was injected to the mixture under nitrogen flow. for the mixture was allowed to stir for 48 h at room temperature, then dried under rotary evaporator, redissolved in hexanes (1.5 L), washed with HCl (2 x 500 mL, pH 3), saturated sodium bicarbonate (2 x 500 mL), brine (1 x 500 mL), dried over sodium sulfate and then pushed through one inch of neutral aluminum oxide, then dried under vacuum to give a colorless oil in 120 g yield (**85%**).

^{13}C NMR (101 MHz, $CDCl_3$) δ 178.02, 173.44, 137.83, 129.04, 128.23, 125.30, 77.32, 77.21, 77.00, 76.69, 74.16, 68.52, 63.54, 47.81, 45.17, 42.74, 38.46, 33.91, 27.46, 26.45, 26.36, 25.45, 24.38, 23.34, 21.45, 17.95, 14.06, 13.80, 1.40, 1.16, 1.03, 0.98, 0.97, 0.95, 0.94, 0.91, 0.66, 0.17, 0.10.
^{1}H NMR (400 MHz, $CDCl_3$) δ 6.31 (s, 1H), 4.41 – 4.04 (m, 2H), 3.76 – 3.59 (m, 2H), 3.46 (dt, *J* = 14.1, 7.2 Hz, 4H), 3.30 (s, 2H), 2.69 (d, *J* = 1.4 Hz, 2H), 2.35 (t, *J* = 7.5 Hz, 2H), 1.56 (s, 18H), 1.44 – 1.16 (m, 4H), 1.03 – 0.81 (m, 5H), 0.73 – 0.48 (m, 5H), 0.09 (s, 490H).

***Synthesis of graft copolymers by ROMP* (5)**
All polymerizations were performed at room temperature in 20 mL glass vials equipped with a magnetic stir bar, under nitrogen atmosphere. Stock solutions of monomers in toluene were prepared at 20 mg/mL. Stock solutions of G3 were prepared at 1 mg/mL and used immediately.

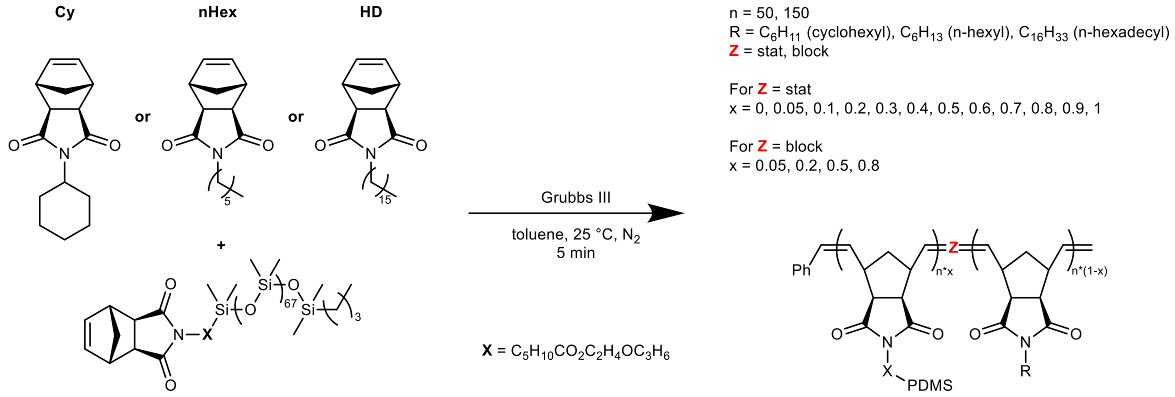

*Synthesis of **statistical** graft copolymers*
A representative procedure for the synthesis of a *statistical*, **cHex**, N_{bb} = 50, GD = 0.5 copolymer is provided: **cHex**, **NB-PDMS** were mixed in a molar ratio of 1:1 and allowed to stir for 5 minutes. G3 stock solution was injected under nitrogen flow at a molar ratio of 1:25:25 (G3 : **cHex** : **NB-PDMS**). The mixture was allowed to stir for 60 minutes before the vial was uncapped and an excess of ethyl vinyl ether, and SiliaMetS DMT was added to quench and sequester the catalyst. After stirring overnight, the mixtures were filtered, and nearly dried under rotary evaporator. The concentrated solutions were transferred to a vacuum oven, and left under vacuum at room temperature for 72 h.

*Synthesis of **block** graft copolymers*
A representative procedure for the synthesis of a *block*, **cHex**, N_{bb} = 50, GD = 0.5 copolymer is provided: **cHex**, was added to a vial under nitrogen flow. G3 stock solution was injected under nitrogen flow at a molar ratio of 1:25 (GG3III : **cHex**). The mixture was allowed to stir for 10 minutes before **NB-PDMS** solution was injected under nitrogen flow at a molar ratio of 1 : 25 : 25 (**NB-** G3 : **PDMS** : : **cHex**). The mixture was allowed to stir for 45 minutes before the vial was uncapped and an excess of ethyl vinyl ether, and SiliaMetS DMT was added to quench and sequester the catalyst. After stirring overnight, the mixtures were filtered, and nearly dried under rotary evaporator. The concentrated solutions were transferred to a vacuum oven, and left under vacuum at room temperature for 72 h to dry.

Representative ^{1}H NMR (***block*-nHexDec-50-0.5**) (^{1}H NMR (400 MHz, $CDCl_3$) δ 5.76 (s, 1H), 5.63 – 5.42 (m, 1H), 4.20 (s, 1H), 3.61 (s, 1H), 3.42 (t, *J* = 6.6 Hz, 3H), 3.02 (s, 1H), 2.67 (s, 1H), 2.32 (d, *J* = 7.3 Hz, 1H), 1.55 (s, 20H), 1.25 (s, 20H), 1.01 – 0.73 (m, 4H), 0.64 – 0.41 (m, 2H), 0.07 (s, 250H).

Standard procedure for the determination of homopolymerization rate constants. A 40 mL vial was charged with a stir bar, linear polystyrene (internal standard, 2 MDa, ~5 mg), **cHex** (0.1631 mmol, 40.00 mg, 1.00 eq) and toluene (32.61 mL) at 298 K, to bring the concentration of **cHex** to 5 mM. Separately, 7 x 4 mL vials were charged with ethyl vinyl ether (EVE, 500 µL). An aliquot of the mixture (50 µL) was added to the first of 8 vials as a t=0 reference. While stirring vigorously, the polymerization was initiated by adding a toluene solution of G3 to achieve initial conditions of monomer (5 mM each) and G3 (0.05 mM). Aliquots were removed by micropipette at time and quenched in EVE-containing vials. The quenched reaction mixtures were analyzed by size exclusion chromatography, allowing the quantification of monomer concentrations at time *t* relative to time 0. Homopolymerization rates were determined by the slope of the natural logarithm of monomer concentration at time t over time = 0 plotted versus time and corrected for G3 concentration, according to the integrated form of the equation below:

$$-d[M]_t/dt = k_{obs}[M]_t = k_{homo}[G3]_0[M]_t$$

Standard procedure for the determination of copolymerization rate constants. A 20 mL vial was charged with a stir bar, linear polystyrene (internal standard, 2 MDa, ~5 mg), **NbPDMS** (0.02724 mmol, 143.3 mg, 1.000 eq) and **cHex** (0.0272 mmol, 6.67 mg, 1.00 eq) and toluene (5.45 mL) at 298 K, to bring the concentration of each monomer to 5 mM. Separately, 7 x 4 mL vials were charged with ethyl vinyl ether (EVE, 500 µL). An aliquot of the mixture (50 µL) was added to the first of 8 vials as a t=0 reference. While stirring vigorously, the polymerization was initiated by adding a toluene solution of G3 to achieve initial conditions of monomer (5 mM each) and G3 (0.05 mM). Aliquots were removed by micropipette at time and quenched in EVE-containing vials. The quenched reaction mixtures were analyzed by size exclusion chromatography, allowing the quantification of monomer concentrations at time *t* relative to time 0. Values of k_{21} and k_{12} were determined by fitting kinetic data with best numerical solutions using MATLAB according to previously described procedures by Grubbs, and using 0, 100 and 1000 M^{-1} s^{-1} as the low boundary, initial estimate, and high boundary for k_{12} and k_{21} solving.[1,2]

Characterization

Nuclear magnetic resonance spectroscopy (NMR)

^{1}H and ^{13}C NMR spectra were recorded using either a 300 or 500 MHz Varian Inova or a 400 or 600 MHz Bruker AVANCE NMR spectrometer. The instrument used for each individual experiment is indicated below where NMR chemical shifts are listed. The NMR spectra were referenced to the relevant residual solvent peak. Chemical shifts are reported as parts per million (ppm) and splitting patters are designated as follows: s (singlet), d (doublet), t (triplet), q (quadruplet), m (multiplet), and b (broad). ^{1}H DOSY were done using a 400 or 500 Bruker AVANCE NMR spectrometer.

Size exclusion chromatography / Gel permeation chromatography (SEC / GPC)*

Analytical gel permeation chromatography (GPC) was performed on a Tosoh EcoSEC HLC-8320 with dual TSKgel SuperH3000 columns and an ethanol-stabilized chloroform mobile phase, with sample concentrations of ~1 mg/mL. Samples were filtered through 0.2 um PTFE syringe filters before injection into the instrument.
*Calculated Mn values were determined by referencing the signal peak to an external calibration curve of linear polystyrene standards. Due to the nature of grafted copolymers and corresponding difference in backbone conformations, Mn values calculated this way should consistently underestimate the actual Mn, and deviate further from actual/theoretical values with increasing macromonomer grafting density.

Small-angle X-ray scattering (SAXS)

SAXS and WAXS experiments were conducted at the Advanced Photon Source (APS) at Argonne National Laboratory (Sector 12-ID-B beamline) using 13.3 keV X-rays. Silver behenate was used as a calibration standard. An exposure time of 0.5 s was used for all samples. The scattering data were recorded as 2D and converted to 1D plots *via* radial averaging. The sample to detector distance was 2001.5 mm for SAXS and 414.884 mm for WAXS. Variable temperature experiments were conducted *in situ* with an electrical heating grid.

Some samples, unable to be acquired at APS, and thus denoted with an asterisk, were analyzed by a Rigaku 002 microfocus X-ray source with an Osmic staggered parabolic multilayer optics, at 0.08 mbar pressure and 1500 mm sample to detector distance, with Dectris Pilatus 300K detector.

Calculation of Volume Fraction

Polymer volume fractions ($f_{PDMS\text{-}MM}$) were calculated by dividing the mass of PDMS macromonomer by the sum total mass of PDMS macromonomer and small molecule comonomer. This approach assumes equivalent room-temperature densities of each component, which we believe to be a reasonable assumption (PDMS is typically reported at ~0.965 g cm^{-3} and polynorbornene at 0.96 g cm^{-3}).

Transmission electron microscopy (TEM)

Sample films of 20-100 nm thickness were cast onto 200 mesh Cu TEM grids from 0.2 mg/mL solutions in toluene and dried under vacuum overnight. Samples were imaged on an FEI Tecnai (G2 Spirit TWIN) TEM using bright field imaging.

Cryo-microtome transmission electron microscopy

Samples were trimmed with a razor blade into a thin post with the top in the shape of a trapezoid. The trapezoid surface was parallel to the thickness of the sample. These trimmed samples were placed in the Leica UC7:FC7 ultramicrotome and cut with a Diatome 35° cryo-AFM diamond knife. 1-189-150-A was cut at -140°C, speed of 1.00 mm/s, and nominal thickness of 70 nm. 1-189-150-B was cut at -110°C, speed of 2.00 mm/s, and nominal thickness of 150 nm. TEM sections were collected onto 460 mesh (hexagonal) copper grids. The samples were post-stained with vapors from 2% osmium tetraoxide solution for 5 minutes. Images were acquired with a ThermoFisher Titan Themis G2 probe-corrected STEM equipped with a Bruker SuperX energy dispersive spectrometer (EDS) and ThermoFisher Ceta CMOS camera.

Grazing incidence small-angle X-ray scattering (GISAXS)

GI-SAXS measurements were conducted at the 11-BM CMS beamline at the National Synchrotron Light Source II at Brookhaven National Lab. The sample to detector (Dectris Pilatus 2M) distance used was 3.03 m, and the wavelength of the beam was 0.9184 Å (13.5 keV). An exposure time of 30 seconds was used.

Dynamic Mechanical Analysis (DMA)

DMA was performed on a TA Discovery DMA 850 system. Samples with dimensions ca. 3 x 1.5 x 10 mm (w x t x l) were tested in tension mode. Measurements were recorded at a frequency of 1 Hz, at fixed strain amplitude of 10 µm from -100–250 °C at a rate of 3 °C min^{-1} with a data sampling interval of 3 s/pt using no force tracking and 0.01 N preload force. DMA data were obtained using Trios software and exported to Microsoft Excel and JMP Pro 14 for analysis. Experiments were performed at the MIT Institute for Soldier Nanotechnologies. Reported G' values are measurements made at 25 °C, and transition temperatures were determined from maximum in tan delta.

Uniaxial Tensile Stress Strain Measurements

Stress strain measurements were made in pentaplicate with a custom screw-driven 8848 MicroTester (Instron Corp.) universal tensile tester with mechanical grips and a 2 kN load cell. Samples were extended at 0.1 mm/s with data collection frequency of 50 Hz, at 20 °C and stretched until failure. Samples were solution-cast from toluene in rectangular Teflon slab molds with internal dimensions of 40 x 35 x 10 mm. After 3 days under vacuum at room temperature, samples were removed from their slab molds, and cut with an arbor press / ASTM-D412-C 0.25 scale die cutter supplied by Fremont Cutting Dies on PTFE liner, yielding dogbones with ~ 10 x 1.65 x 1.0 mm gauge sizes.

Compression Molding

Stat-nHexDec-150-0.05 samples were compression molded under 3 tons of force at 100 °C for 10 minutes on a Carver press to yield molded materials.

Rheological Measurements

Frequency sweep and temperature sweep experiments were performed using a TA Instruments Discovery HR-2 rheometer. An 8 mm parallel plate geometry was used to engage the samples. Sample discs were 8 mm in diameter with a thickness of 1.0-1.5 mm. Samples were made either using a Teflon mold, circular punch, or deposition/compression using a 1.5 mm thick sheet of sample. Frequency sweep experiments were performed from 0.1 to 100 rad/s at 1% strain.

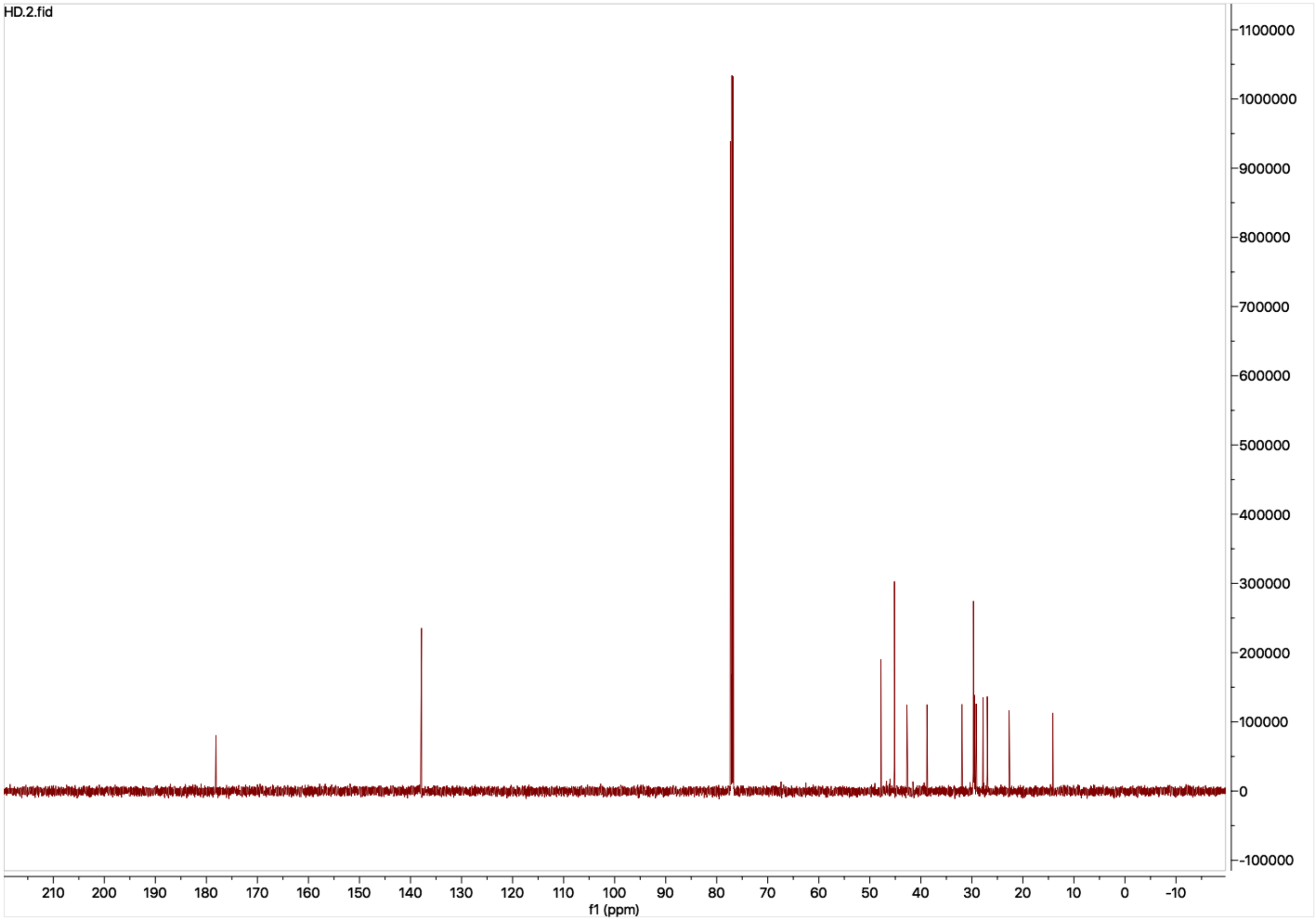

Figure S1: ^{13}C NMR, nHexDec

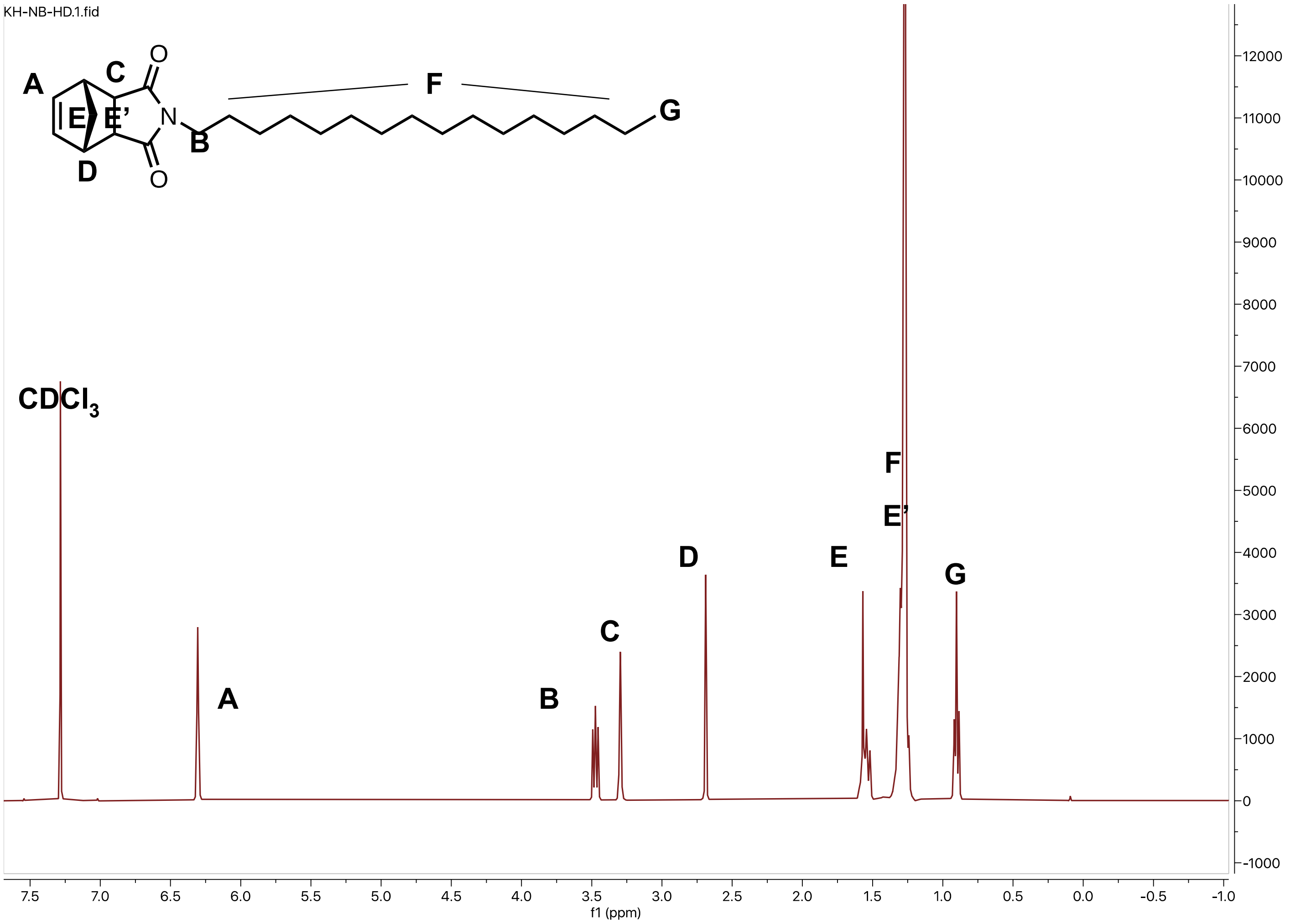

Figure S2: ^{1}H NMR, nHexDec

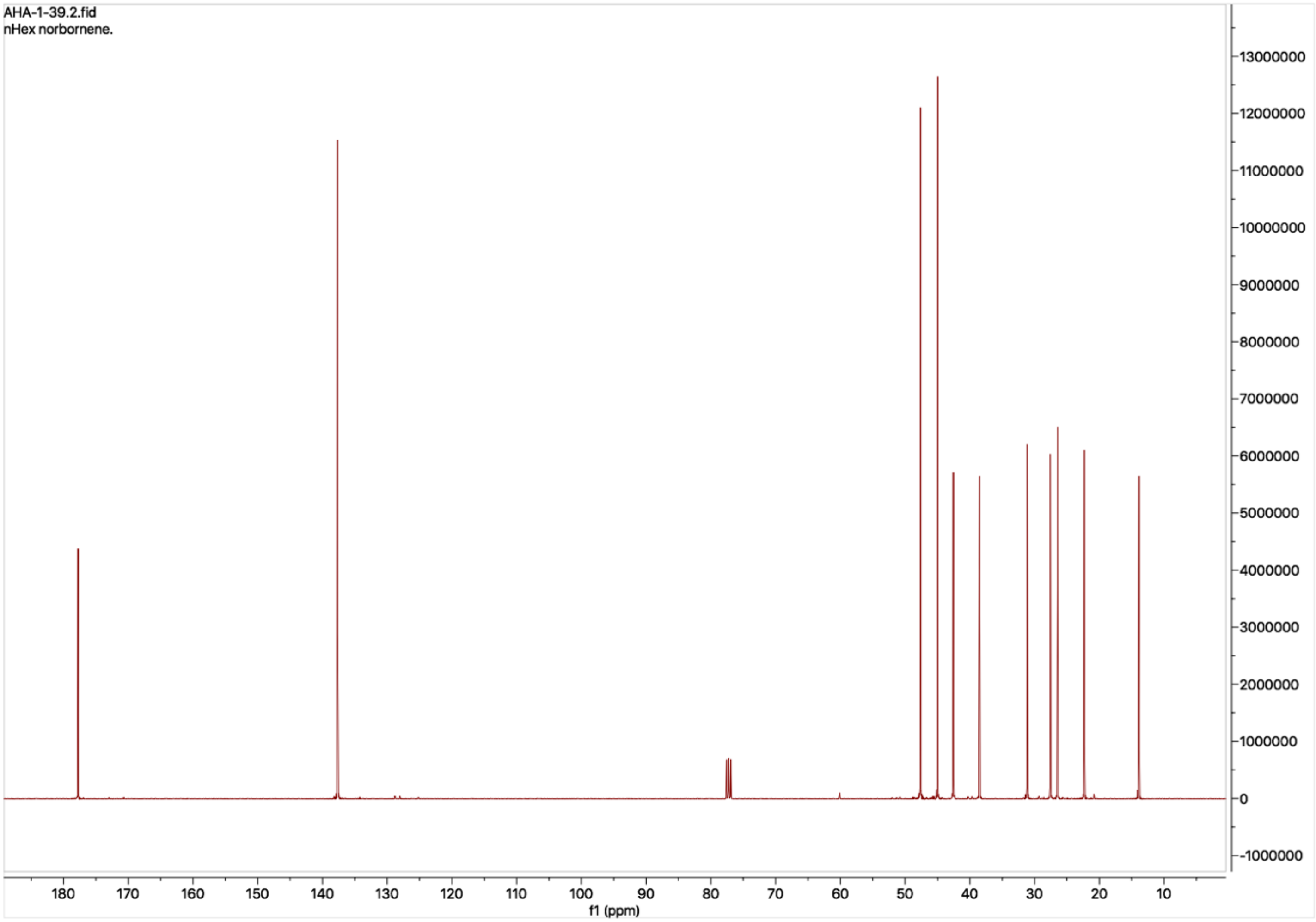

Figure S3: ^{13}C NMR, nHex

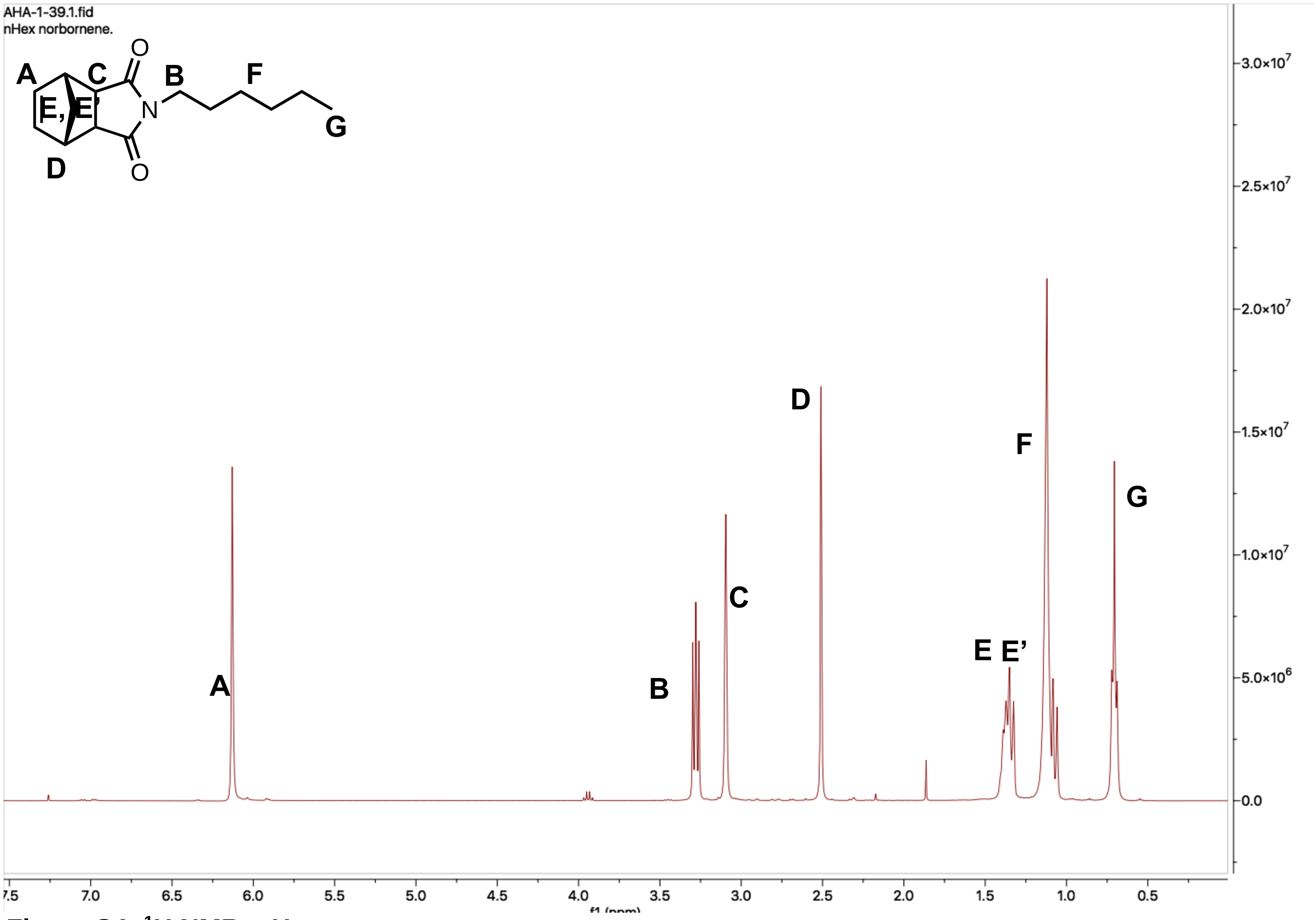

Figure S4: ^{1}H NMR, nHex

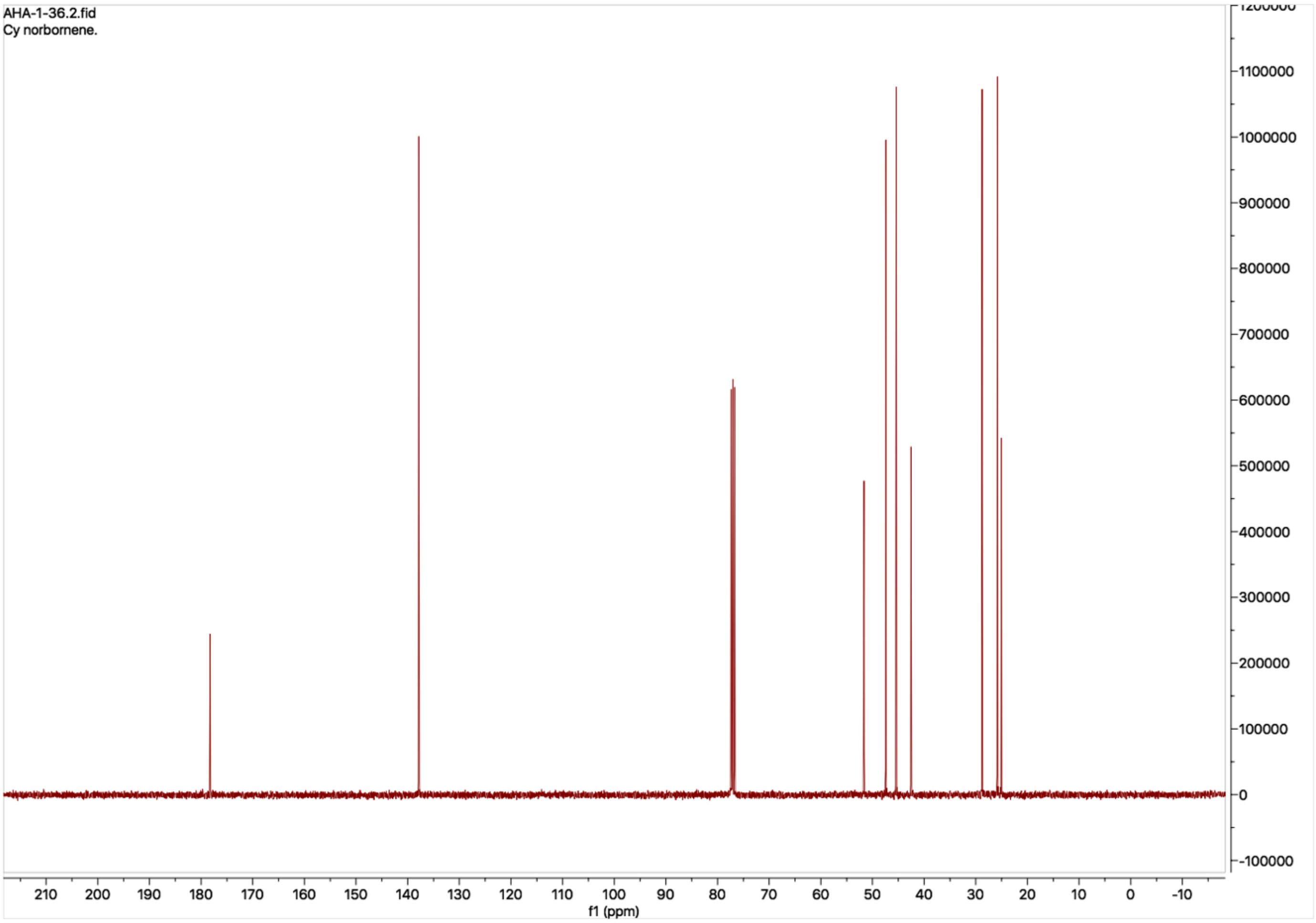

Figure S5: ^{13}C NMR, cHex

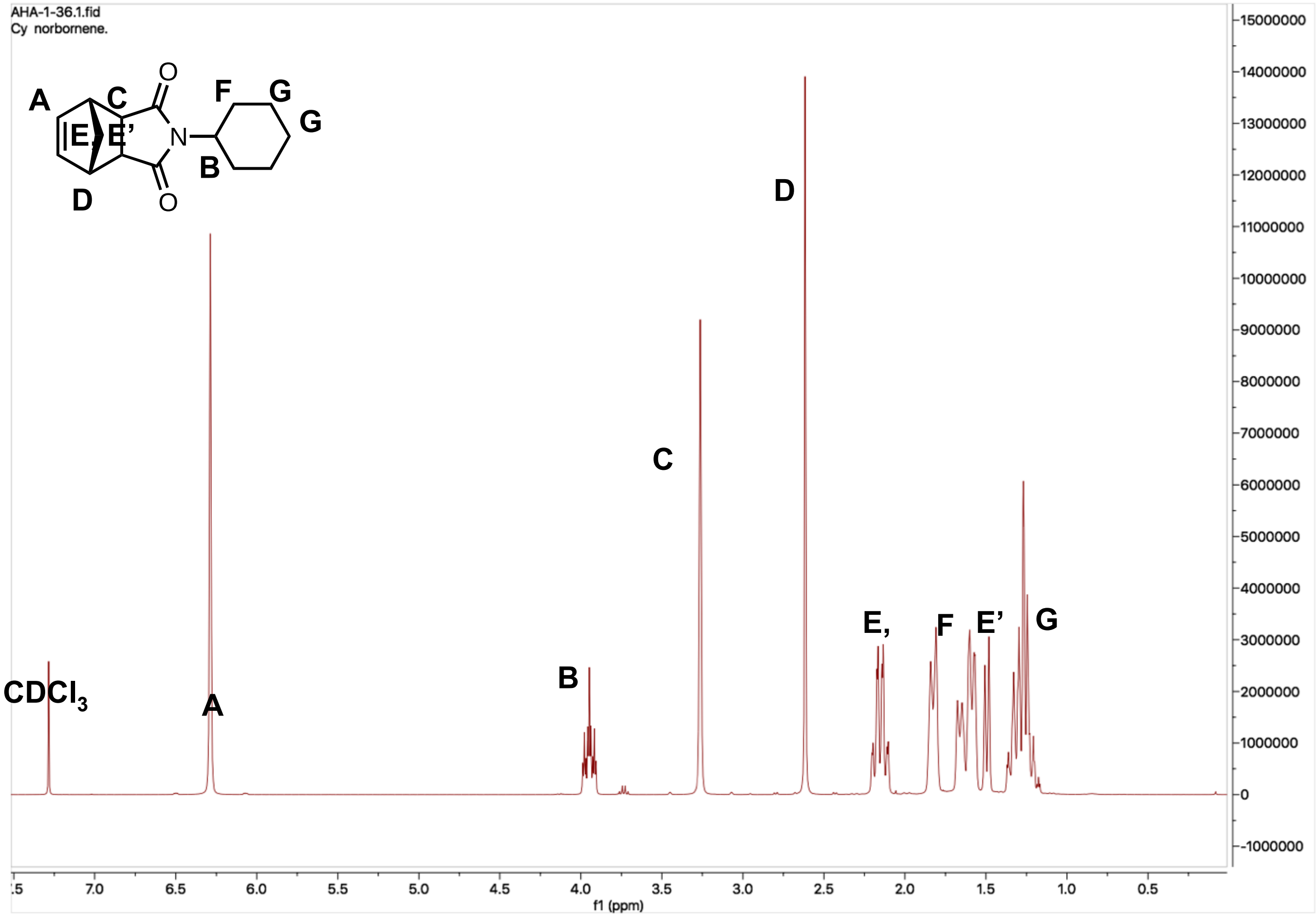

Figure S6: ^{1}H NMR, cHex

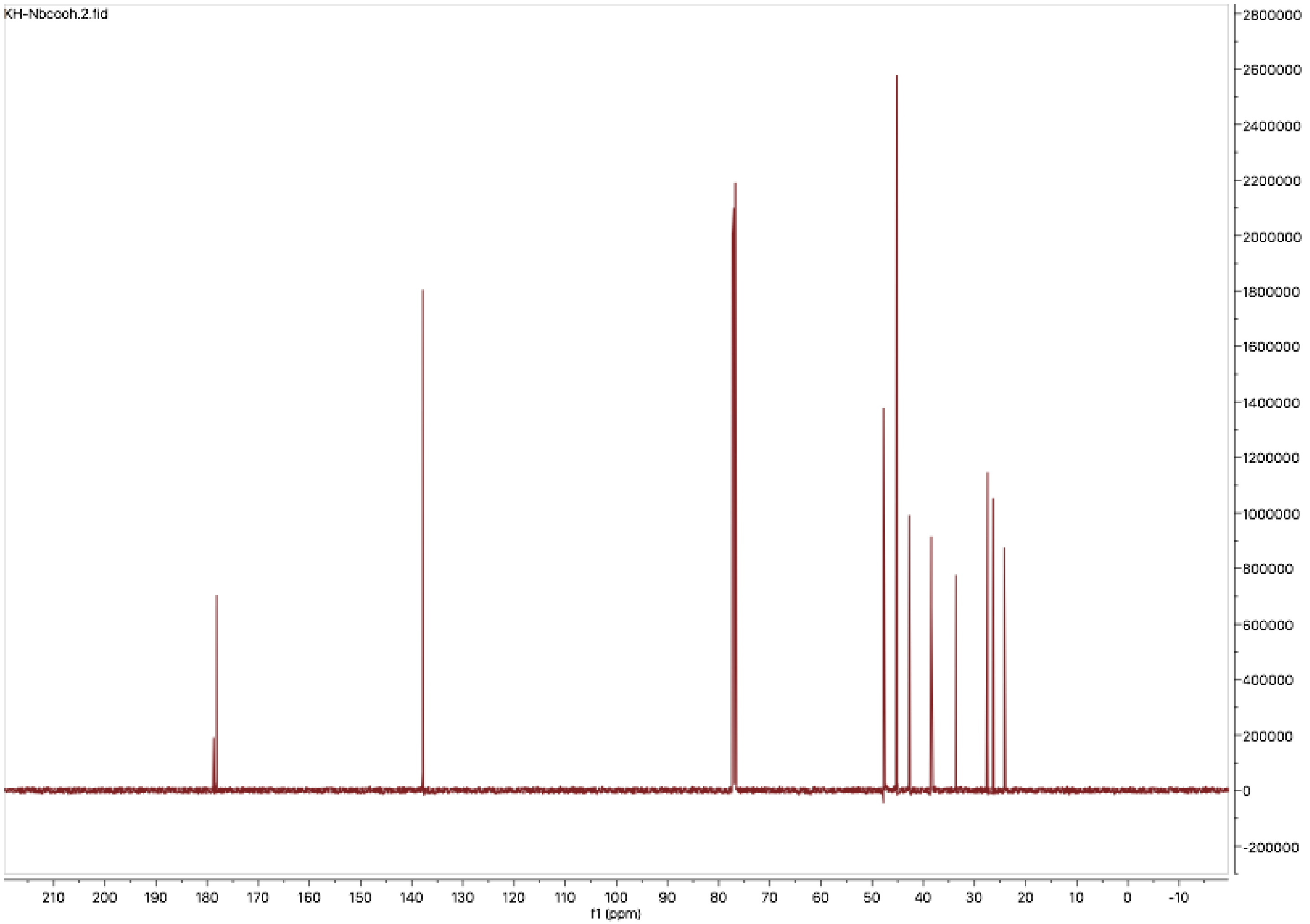

Figure S7: ^{13}C NMR, Nb-COOH

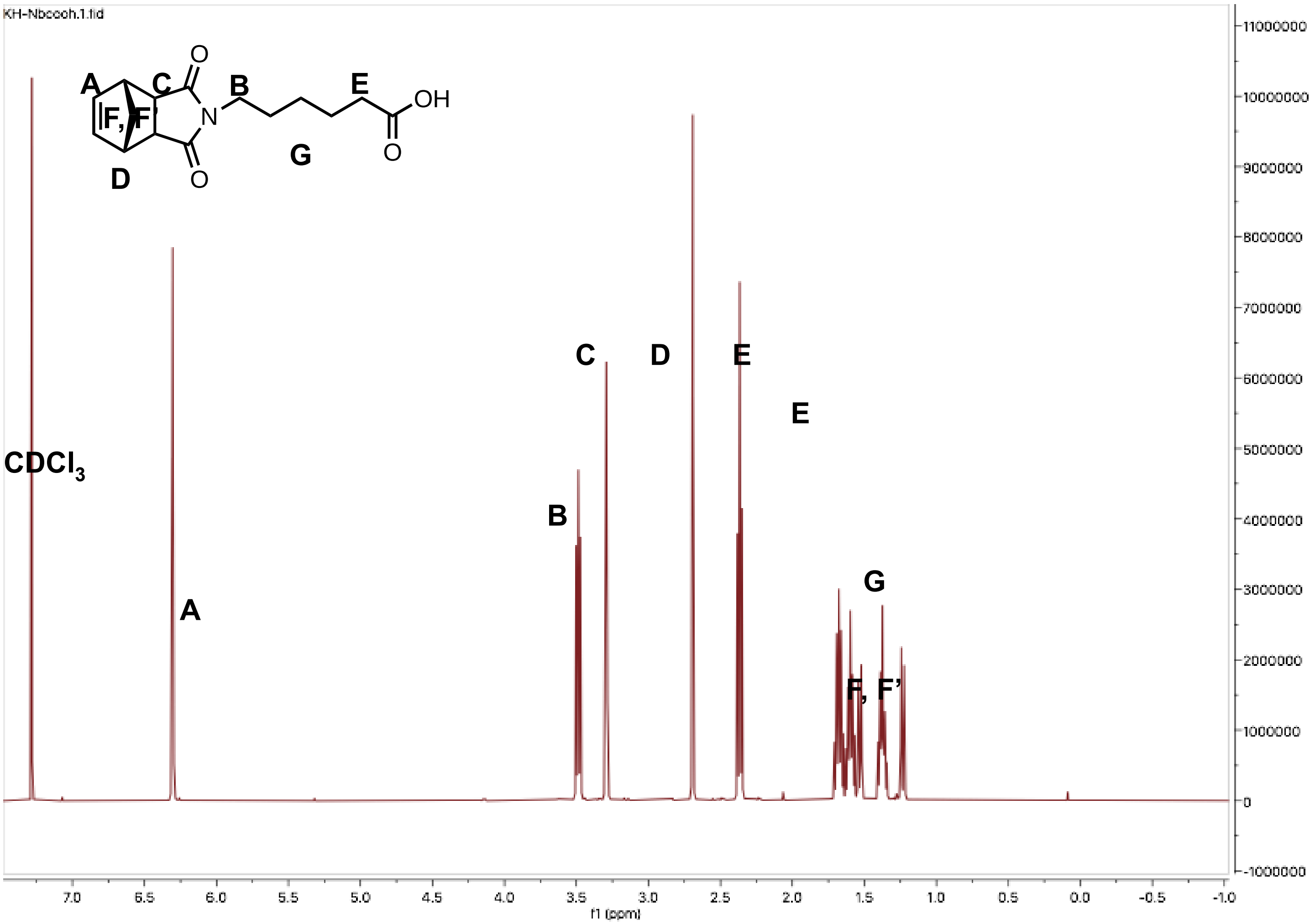

Figure S8: ^{1}H NMR, Nb-COOH

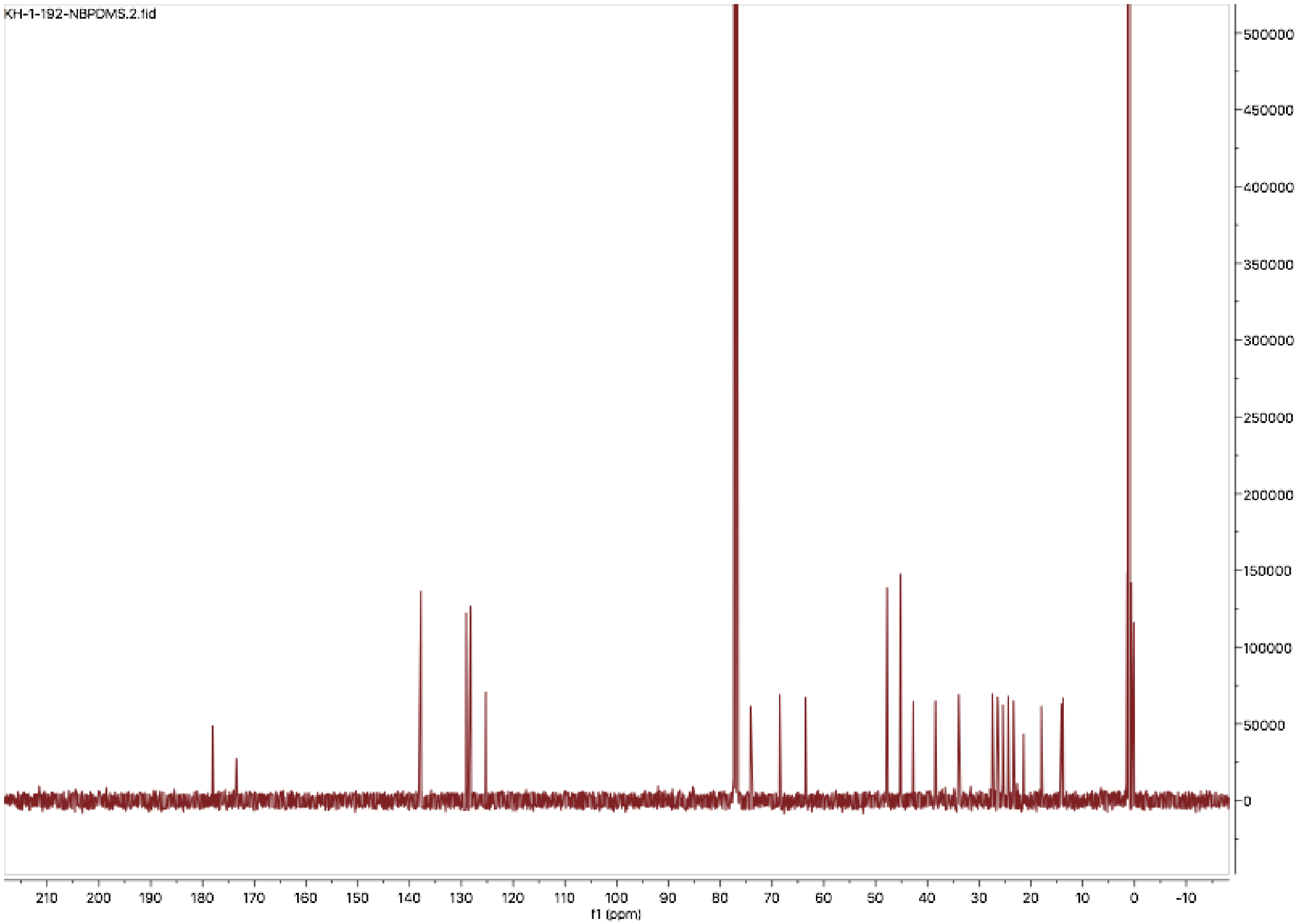

Figure S9: ^{13}C NMR, Nb-PDMS

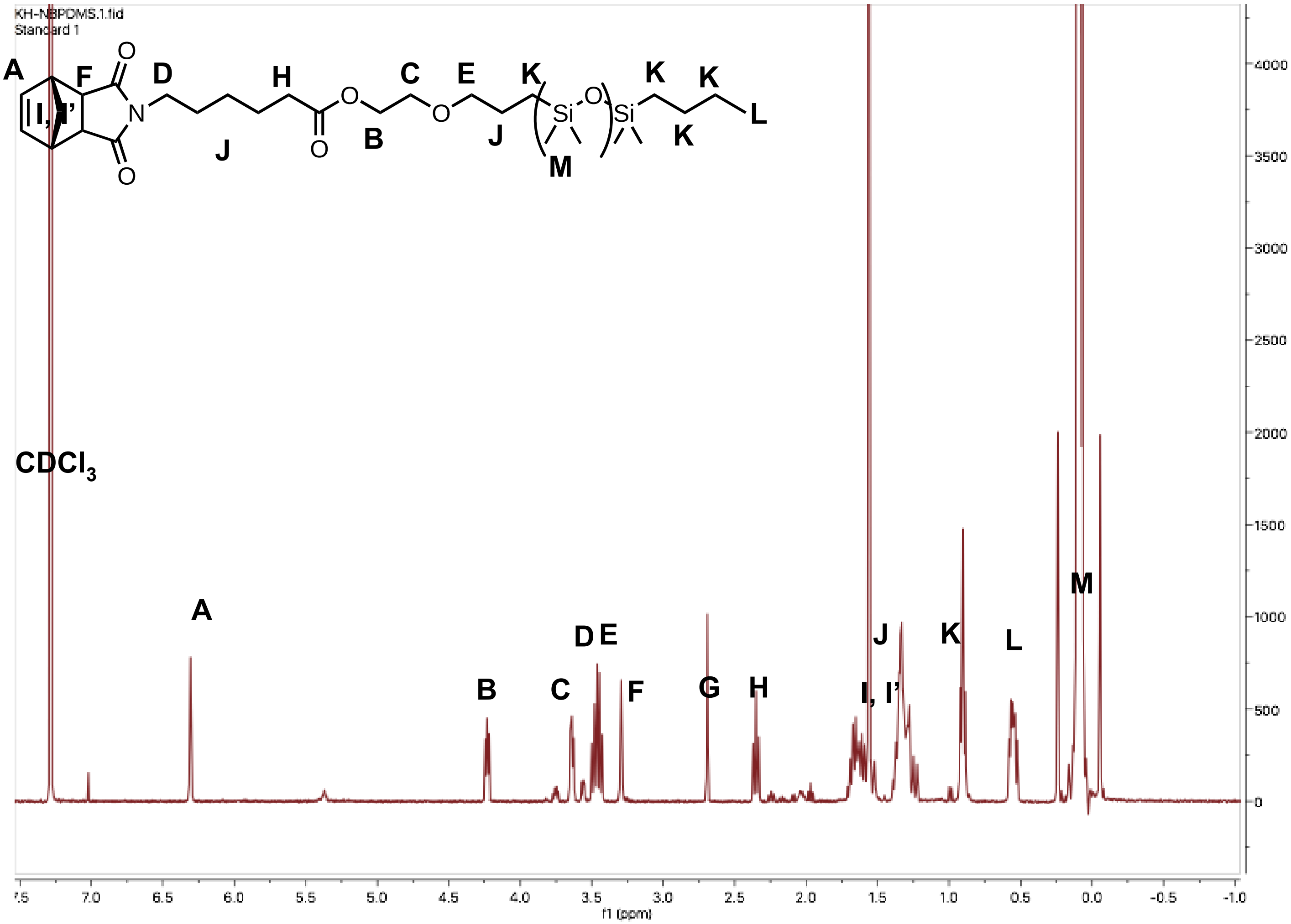

Figure S10: ^{1}H NMR, Nb-PDMS

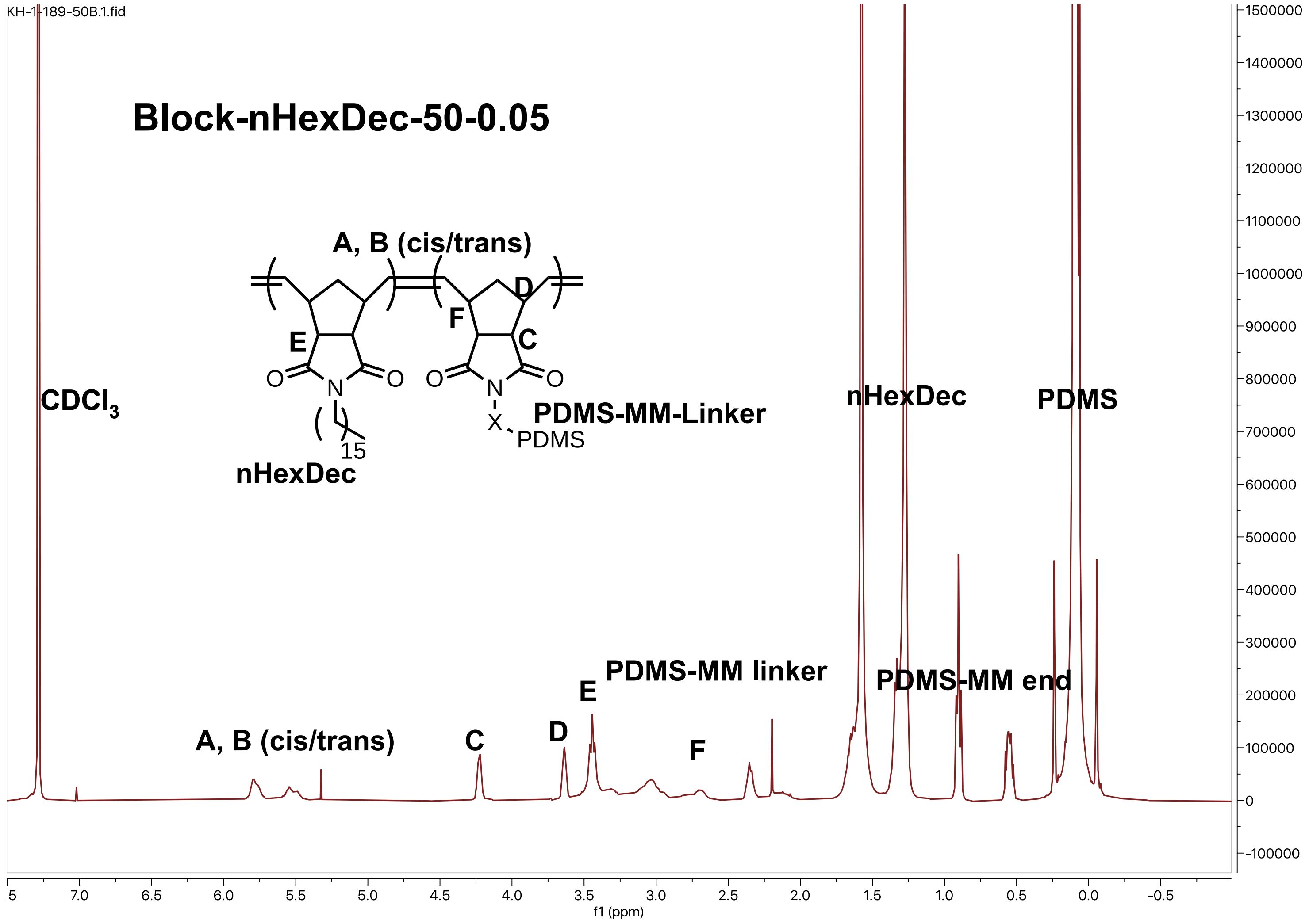

Figure S11: Representative ^{1}H NMR of copolymer (*block*-nHexDec-50-0.5)

Table S1: Characterization of homopolymers

Polymer	Monomer	Mn, expected (kDa)	Mn, calculated (kDa)	N_{bb}	q* (1/nm)	*d** (nm)	Morphology
**1**	PDMS	264	119	50	0.861	7.2975438	D-CYL
**2**	PDMS	792	237	150	0.763	8.234843	D-CYL
**3**	cHex	12	12	50	NA	NA	DIS
**4**	cHex	37	27	150	NA	NA	DIS
**5**	nHex	12	14	50	NA	NA	DIS
**6**	nHex	37	27	150	NA	NA	DIS
**7**	nHexDec	19	22	50	NA	NA	DIS
**8**	nHexDec	58	59	150	NA	NA	DIS

Table S2: Characterization of cHex copolymers

Polymer	Seq.	GD	N_{bb}	Mn, expected (kDa)	Mn, calculated (kDa)	*f* PDMS	q* (1/nm)	*d** (nm)	Morphology
**9**	G	0.9	50	239	87	0.99	0.826	7.61	D-CYL
**10**	G	0.8	50	214	87	0.99	0.806	7.80	D-CYL
**11**	G	0.7	50	188	81	0.98	0.798	7.87	BCC
**12**	G	0.6	50	163	77	0.97	0.764	8.22	BCC
**13**	G	0.5	50	138	71	0.96	0.546	11.51	BCC
**14**	G	0.4	50	113	67	0.93	0.689	9.12	BCC
**15**	G	0.3	50	88	64	0.90	0.633	9.93	BCC
**16**	G	0.2	50	63	42	0.84	0.72345	8.69	BCC
**17**	G	0.1	50	37	30	0.71	0.485	12.96	HEX
**18**	G	0.05	50	25	21	0.53	0.4938	12.72	LAM
**19**	B	0.8	50	214	88	0.99	0.8037	7.82	DIS
**20**	B	0.5	50	138	69	0.96	0.6833	9.20	DIS
**21**	B	0.2	50	63	34	0.84	0.4249	14.79	MIXED
**22**	B	0.05	50	25	20	0.53	0.2986	21.04	LAM
**23**	G	0.9	150	716	190	0.99	0.755	8.32	D-CYL
**24**	G	0.8	150	641	187	0.99	0.7357	DIS	D-CYL
**25**	G	0.7	150	565	177	0.98	0.741	8.48	BCC
**26**	G	0.6	150	490	165	0.97	0.7751	8.11	BCC
**27**	G	0.5	150	414	158	0.96	0.7235	8.68	BCC
**28**	G	0.4	150	339	144	0.93	0.6833	9.20	BCC
**29**	G	0.3	150	263	132	0.90	0.6258	10.04	HEX
**30**	G	0.2	150	188	107	0.84	0.5512	11.40	DIS
**31**	G	0.1	150	112	87	0.70	0.4536	13.85	DIS
**32**	G	0.05	150	75	67	0.53	0.3847	16.33	LAM
**33**	B	0.8	150	641	197	0.99	0.7292	8.62	D-CYL
**34**	B	0.5	150	414	145	0.96	0.2412	26.05	MIXED
**35**	B	0.2	150	188	76	0.84	0.2699	23.28	MIXED
**36**	B	0.05	150	75	63	0.53	0.1952	32.19	LAM

Table S3: Characterization of nHex copolymers

Polymer	Seq.	GD	N_{bb}	M_n, expected (kDa)	Mn, calculated (kDa)	*f* PDMS	q* (1/nm)	*d** (nm)	Morphology
**37**	G	0.9	50	238	87	0.99	0.8383	7.50	D-CYL
**38**	G	0.8	50	214	89	0.99	0.8153	7.71	D-CYL
**39**	G	0.7	50	188	80	0.98	0.7981	7.87	D-CYL
**40**	G	0.6	50	163	77	0.97	0.7751	8.11	BCC
**41**	G	0.5	50	138	74	0.96	0.7235	8.68	BCC
**42**	G	0.4	50	113	68	0.93	0.6775	9.27	BCC
**43**	G	0.3	50	88	66	0.90	0.6201	10.13	HEX
**44**	G	0.2	50	63	45	0.84	0.534	11.77	HEX
**45**	G	0.1	50	38	29	0.70	0.443	14.18	HEX
**46**	G	0.05	50	25	25	0.53	0.3962	15.86	LAM
**47**	B	0.8	50	214	89	0.99	0.8628	7.28	D-CYL
**48**	B	0.5	50	138	70	0.96	0.6775	9.27	DIS
**49**	B	0.2	50	63	41	0.84	0.4077	15.41	HEX
**50**	B	0.05	50	25	21	0.53	0.2584	24.32	LAM
**51**	G	0.9	150	716	189	0.99	0.7694	8.17	D-CYL
**52**	G	0.8	150	641	186	0.99	0.7464	8.42	D-CYL
**53**	G	0.7	150	565	179	0.98	0.7464	8.42	BCC
**54**	G	0.6	150	490	168	0.97	0.768	8.18	BCC
**55**	G	0.5	150	414	160	0.96	0.7292	8.62	BCC
**56**	G	0.4	150	339	148	0.93	0.666	9.43	HEX
**57**	G	0.3	150	263	129	0.90	0.6086	10.32	HEX
**58**	G	0.2	150	188	103	0.84	0.5282	11.90	HEX
**59**	G	0.1	150	113	90	0.70	0.4191	14.99	HEX
**60**	G	0.05	150	75	74	0.53	0.379	16.58	LAM
**61**	B	0.8	150	641	194	0.99	0.7464	8.42	D-CYL
**62**	B	0.5	150	414	148	0.96	0.2182	28.80	MIXED
**63**	B	0.2	150	188	78	0.84	0.2412	26.05	MIXED
**64**	B	0.05	150	75	59	0.53	0.1665	37.74	LAM

Table S4: Characterization of nHexDec copolymers

Polymer	Seq.	GD	N_{bb}	M_n, expected (kDa)	Mn, calculated (kDa)	*f* PDMS	q* (1/nm)	*d** (nm)	Morphology
**65**	G	0.9	50	239	87	0.99	0.8383	7.50	D-CYL
**66**	G	0.8	50	215	80	0.98	0.8153	7.71	D-CYL
**67**	G	0.7	50	191	86	0.97	0.7981	7.87	D-CYL
**68**	G	0.6	50	166	76	0.95	0.7636	8.23	D-CYL
**69**	G	0.5	50	142	73	0.93	0.7235	8.68	D-CYL
**70**	G	0.4	50	117	63	0.90	0.666	9.43	D-CYL
**71**	G	0.3	50	93	42	0.85	0.6258	10.04	LAM
**72**	G	0.2	50	68	39	0.77	0.5971	10.52	LAM
**73**	G	0.1	50	44	36	0.60	0.4479	14.03	LAM
**74**	G	0.05	50	32	30	0.42	0.3675	17.10	LAM
**75**	B	0.8	50	215	71	0.98	0.8153	7.71	D-CYL
**76**	B	0.5	50	142	70	0.93	0.79	7.95	MIXED
**77**	B	0.2	50	68	39	0.77	0.3388	18.55	MIXED
**78**	B	0.05	50	32	29	0.42	0.2641	23.79	LAM
**79**	G	0.9	150	718	194	0.99	0.7579	8.29	D-CYL
**80**	G	0.8	150	645	175	0.98	0.7522	8.35	D-CYL
**81**	G	0.7	150	572	180	0.97	0.7579	8.29	D-CYL
**82**	G	0.6	150	498	167	0.95	0.7522	8.35	D-CYL
**83**	G	0.5	150	425	157	0.93	0.712	8.82	D-CYL
**84**	G	0.4	150	352	146	0.90	0.6718	9.35	D-CYL
**85**	G	0.3	150	278	138	0.85	0.6316	9.95	LAM
**86**	G	0.2	150	205	104	0.77	0.6086	10.32	LAM
**87**	G	0.1	150	131	92	0.60	0.4421	14.21	LAM
**88**	G	0.05	150	95	79	0.42	0.3695	17.00	LAM
**89**	B	0.8	150	645	192	0.98	0.7464	8.42	D-CYL
**90**	B	0.5	150	425	149	0.93	0.2067	30.40	MIXED
**91**	B	0.2	150	205	90	0.77	0.201	31.26	MIXED
**92**	B	0.05	150	95	79	0.42	0.1608	39.07	LAM

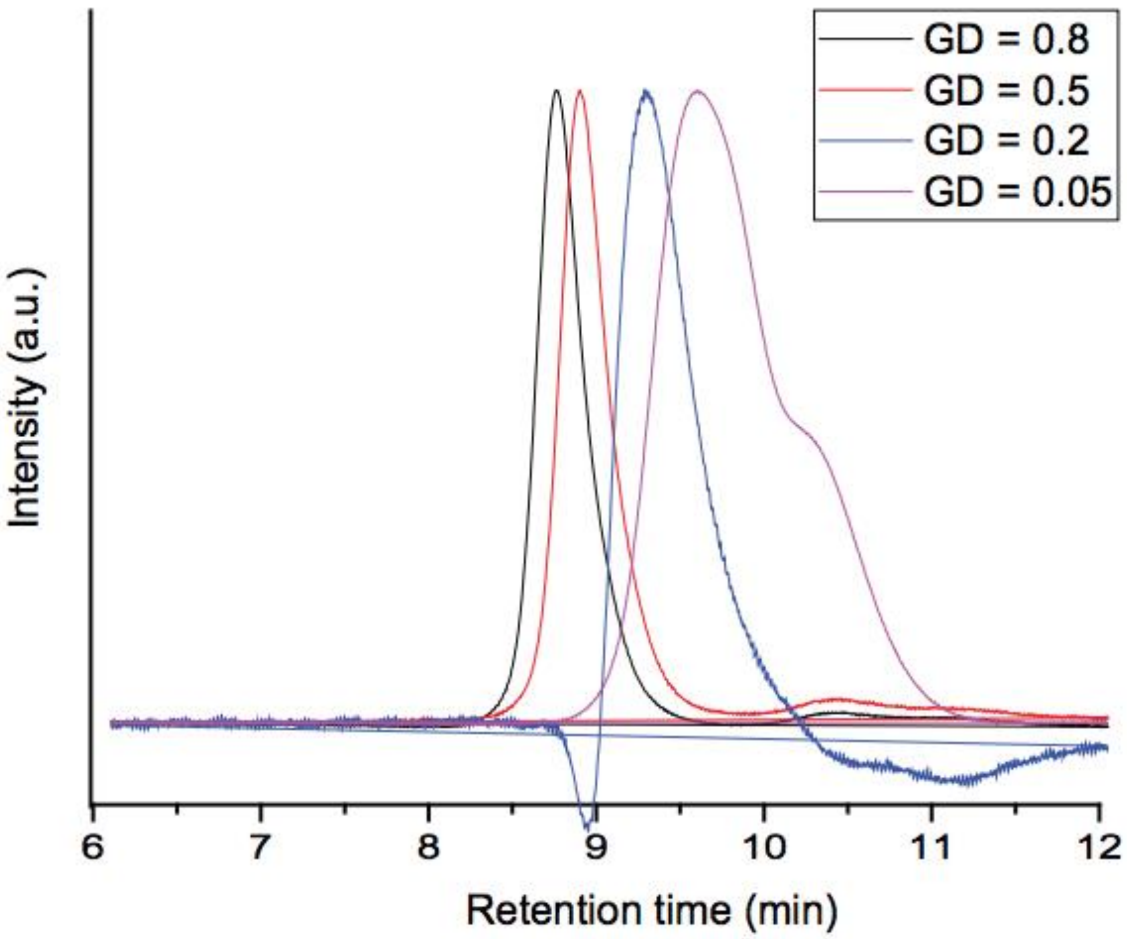

Figure S12: SEC traces of N_{bb} = 50, cHex *block* copolymers

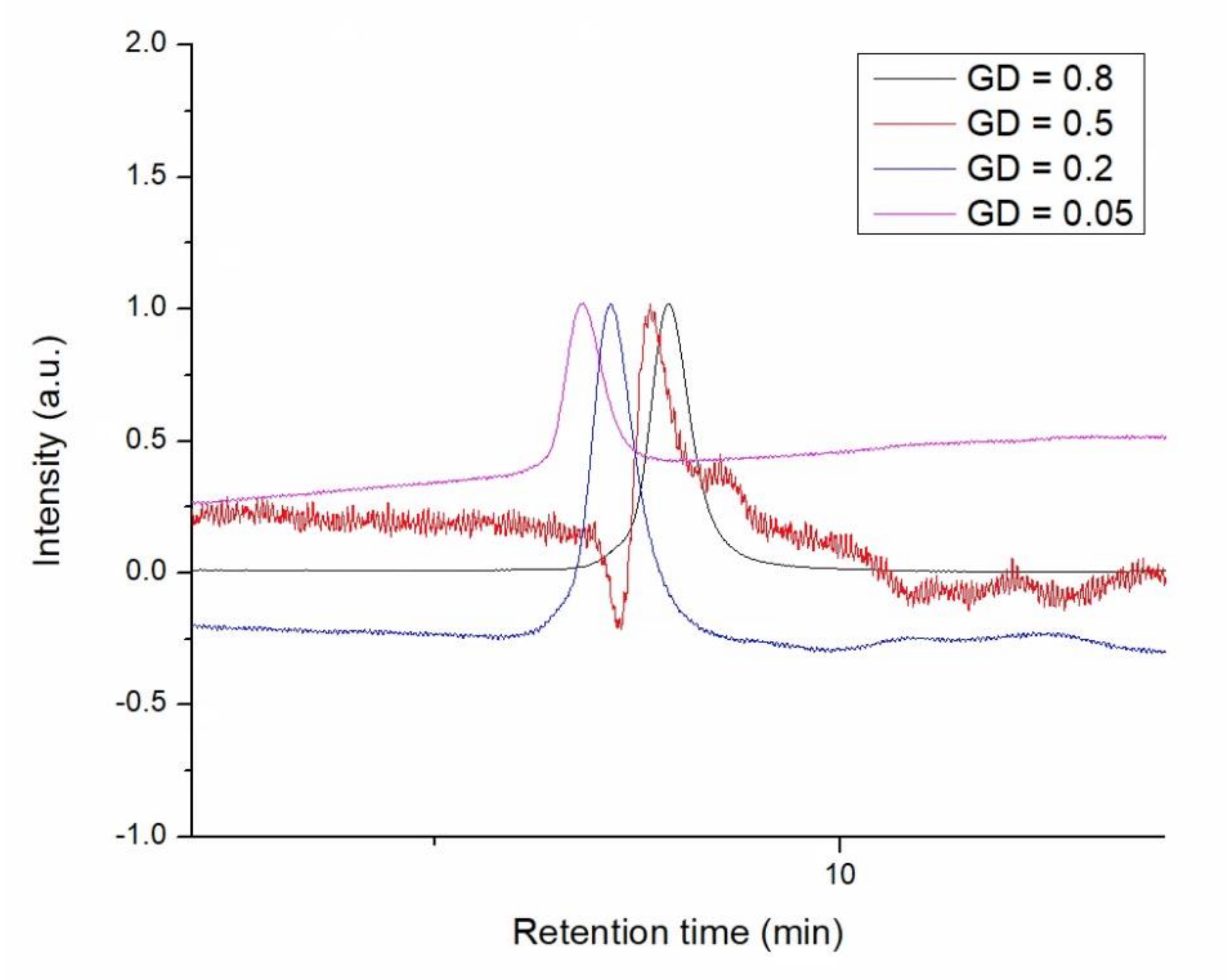

Figure S13: SEC traces of N_{bb} = 150, cHex *block* copolymers

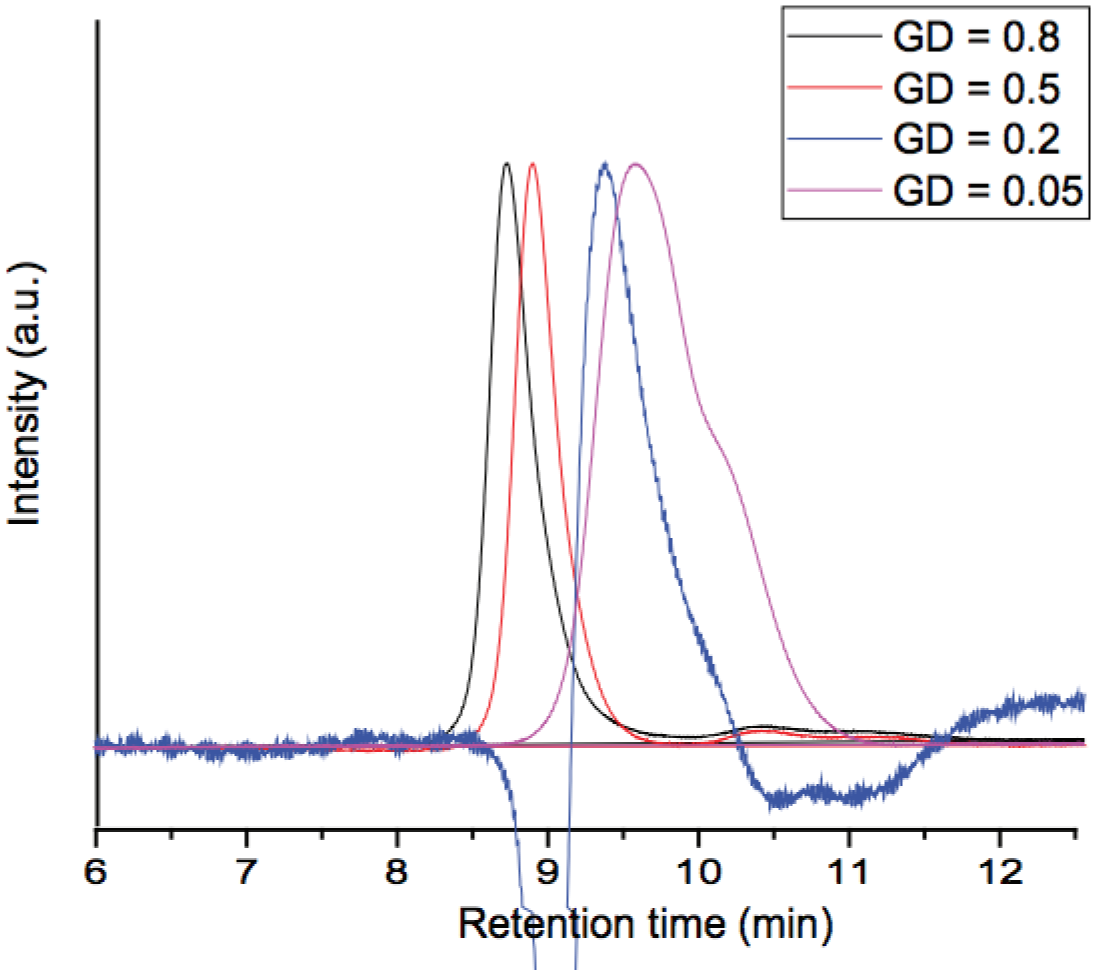

Figure S14: SEC traces of N_{bb} = 50, nHex *block* copolymers

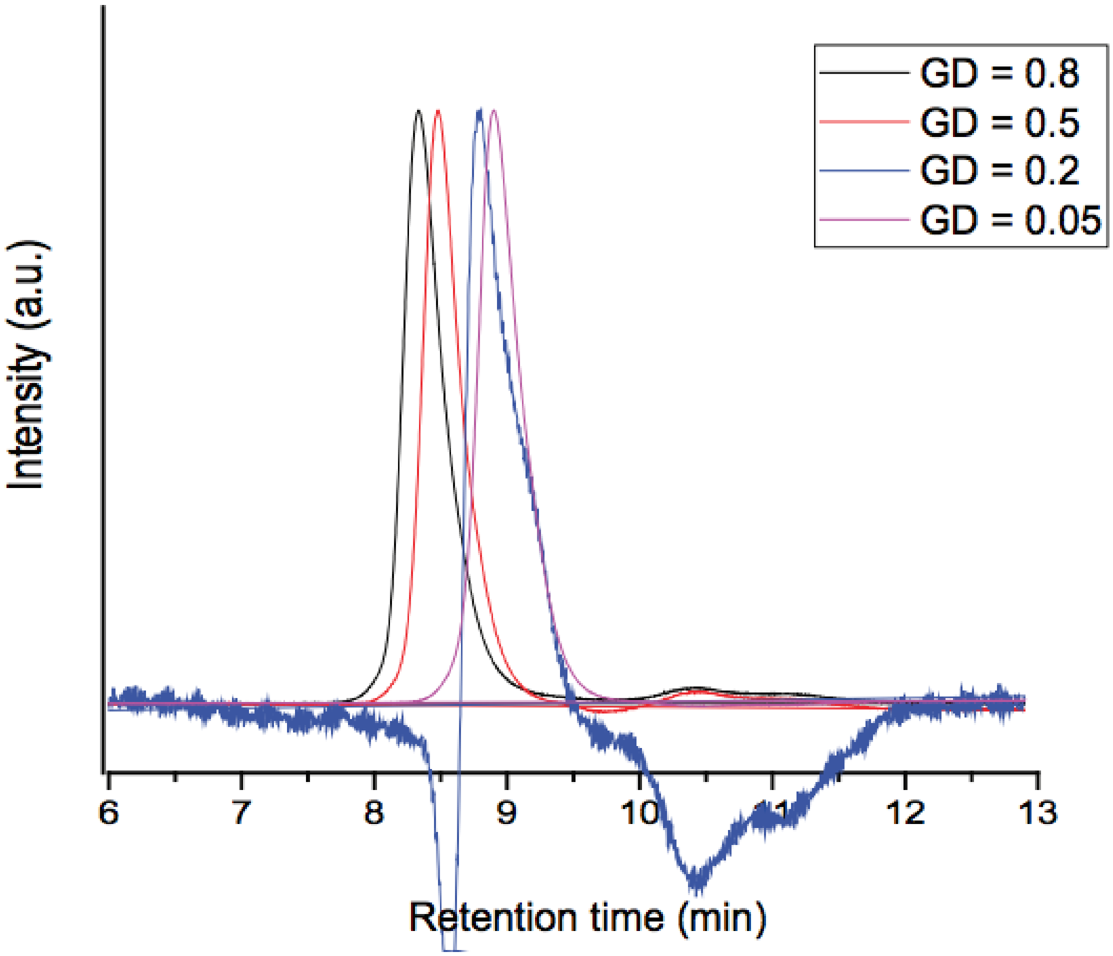

Figure S15: SEC traces of N_{bb} = 150, nHex *block* copolymers

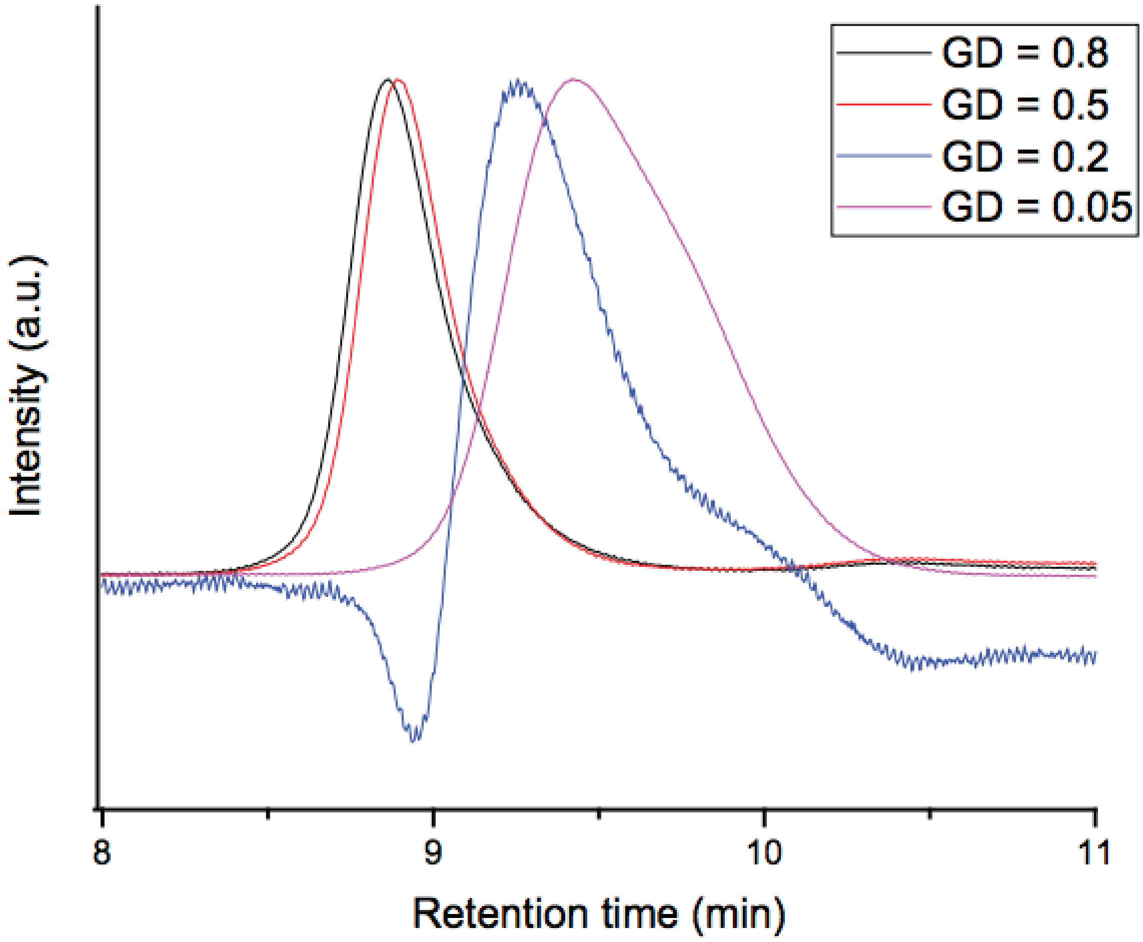

Figure S16: SEC traces of N_{bb} = 50, nHexDec *block* copolymers

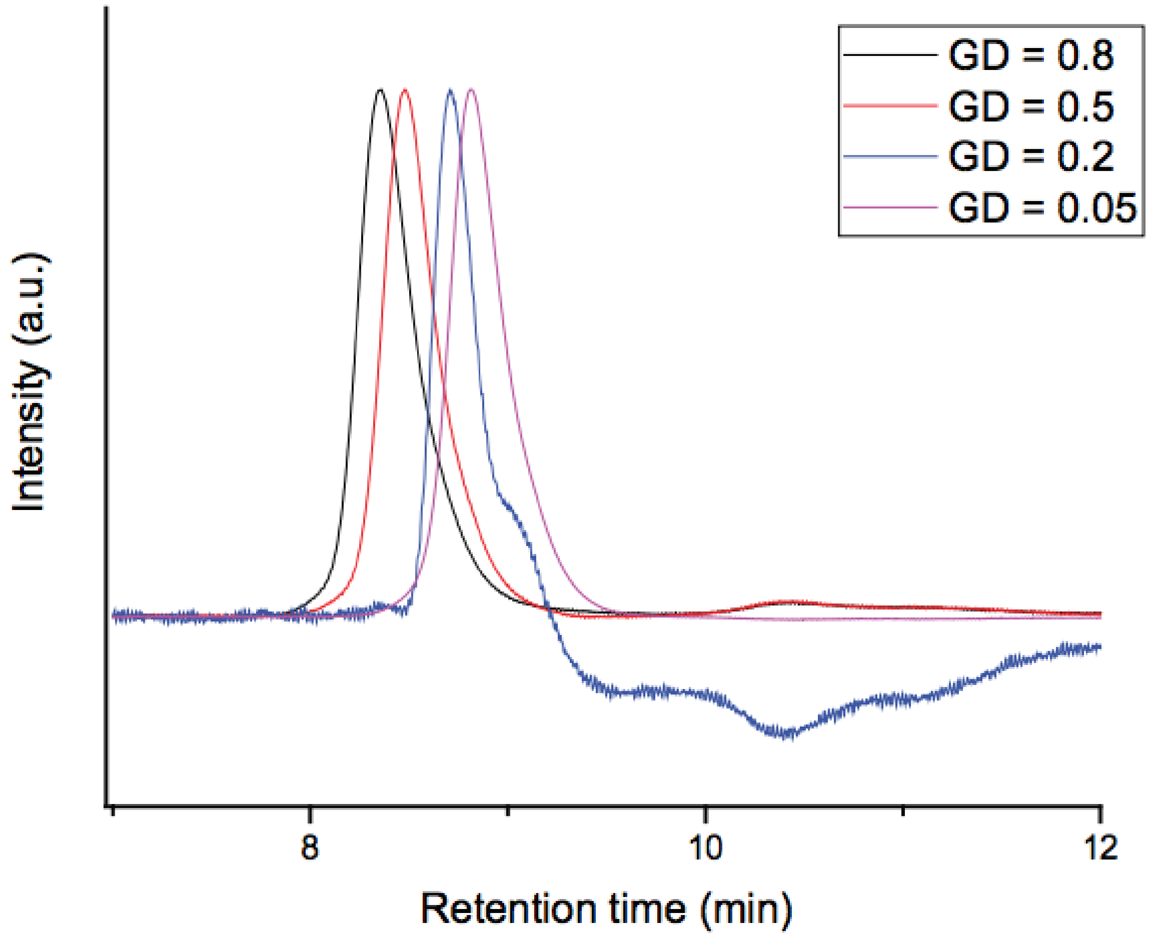

Figure S17: SEC traces of N_{bb} = 150, nHexDec *block* copolymers

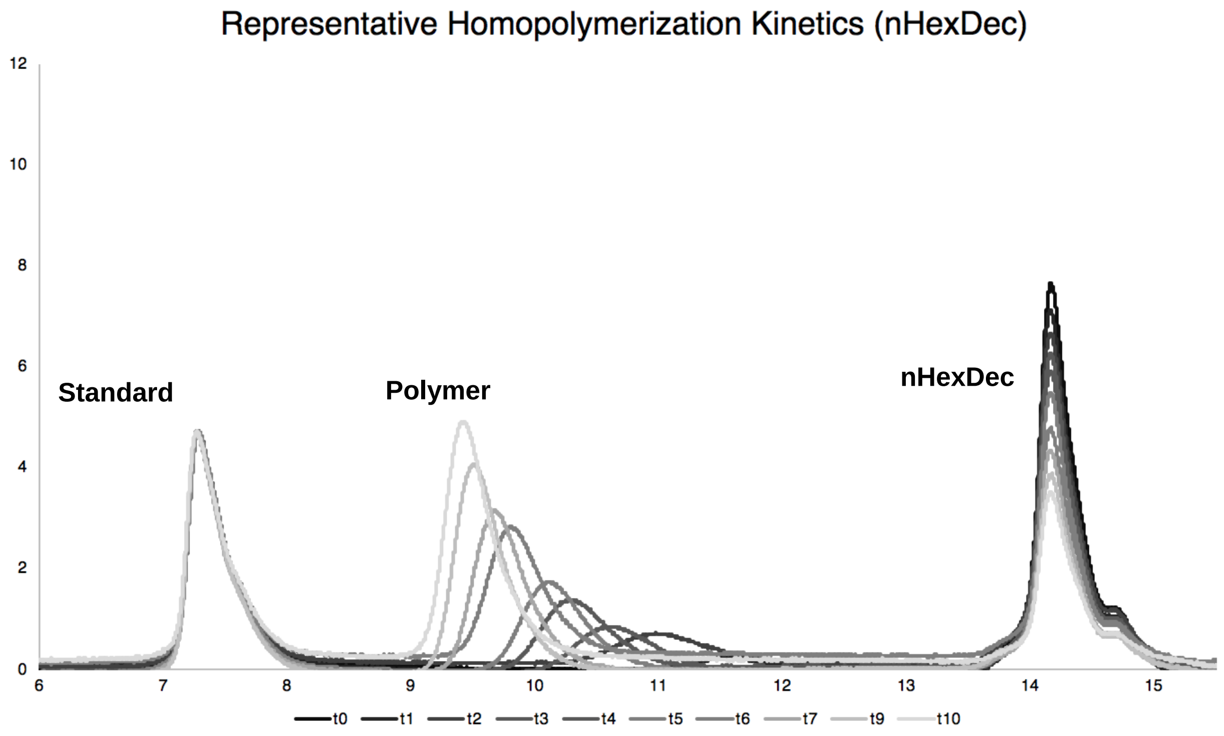

Figure S18: Representative SEC traces for homopolymerization kinetics analysis

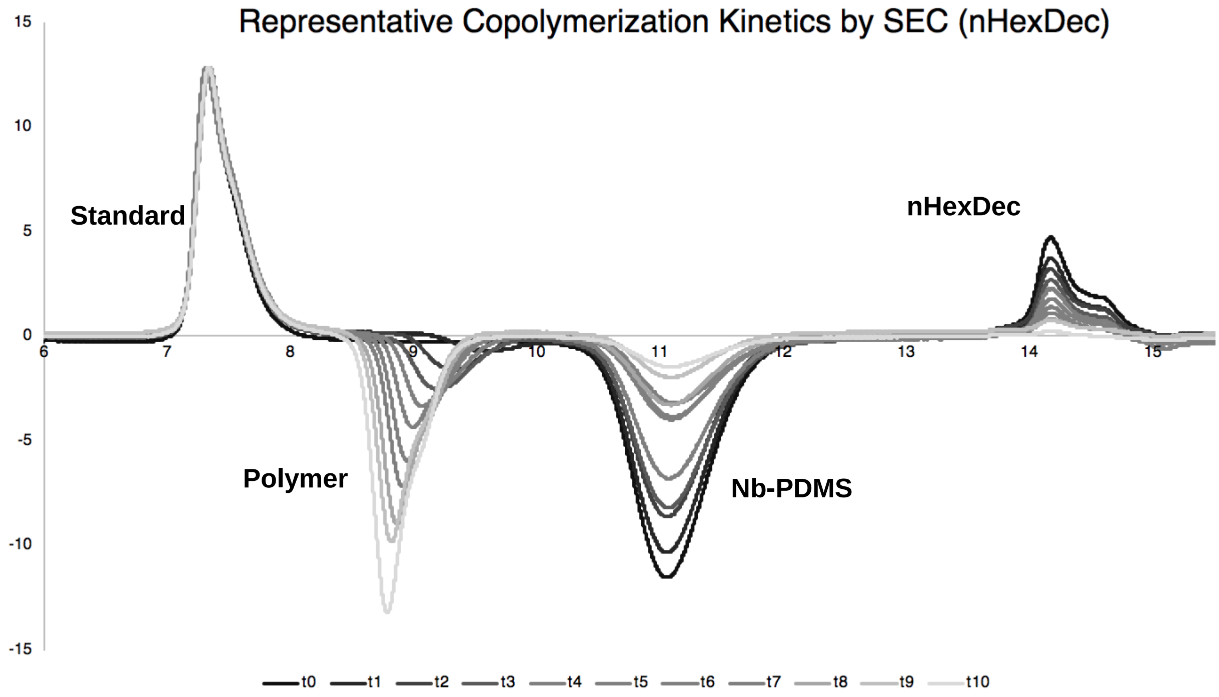

Figure S19: Representative SEC traces for copolymerization kinetics analysis

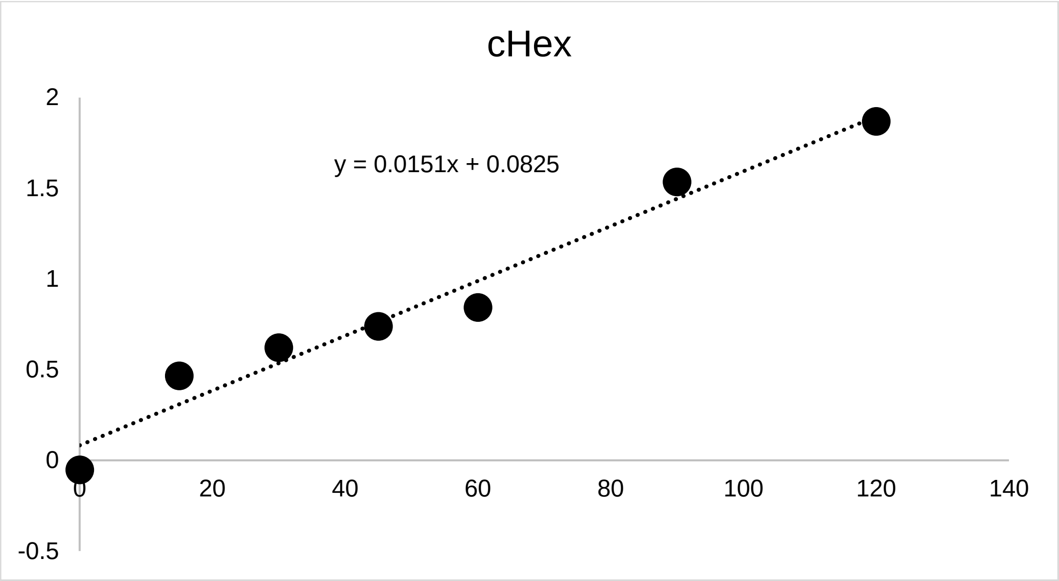

Figure S20: Representative homopolymerization kinetics, cHex. Time (seconds) vs ln(M_0/M_t).

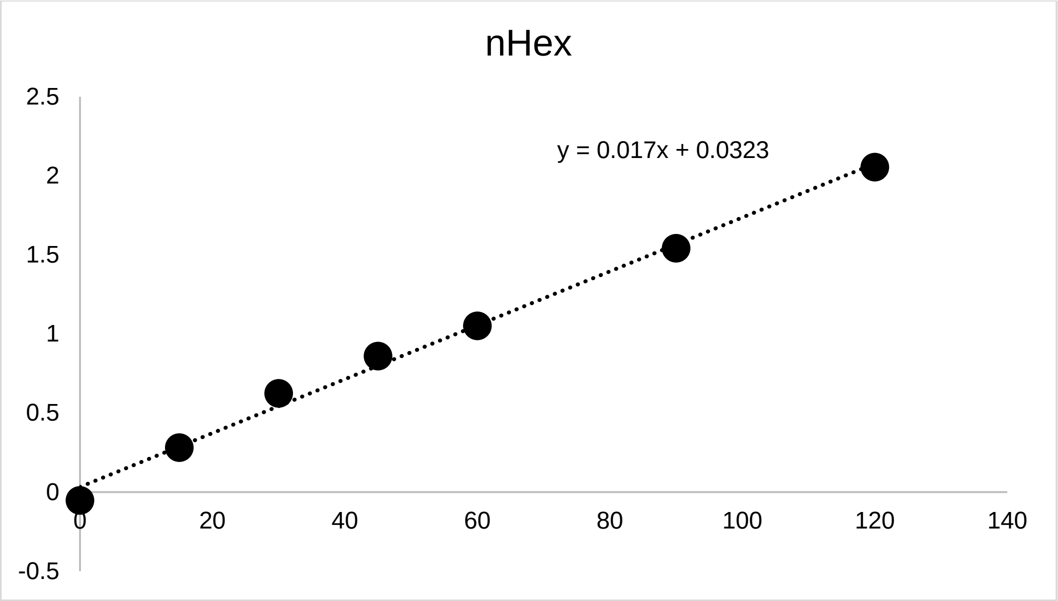

Figure S21: Representative homopolymerization kinetics, nHex. Time (seconds) vs ln(M_0/M_t).

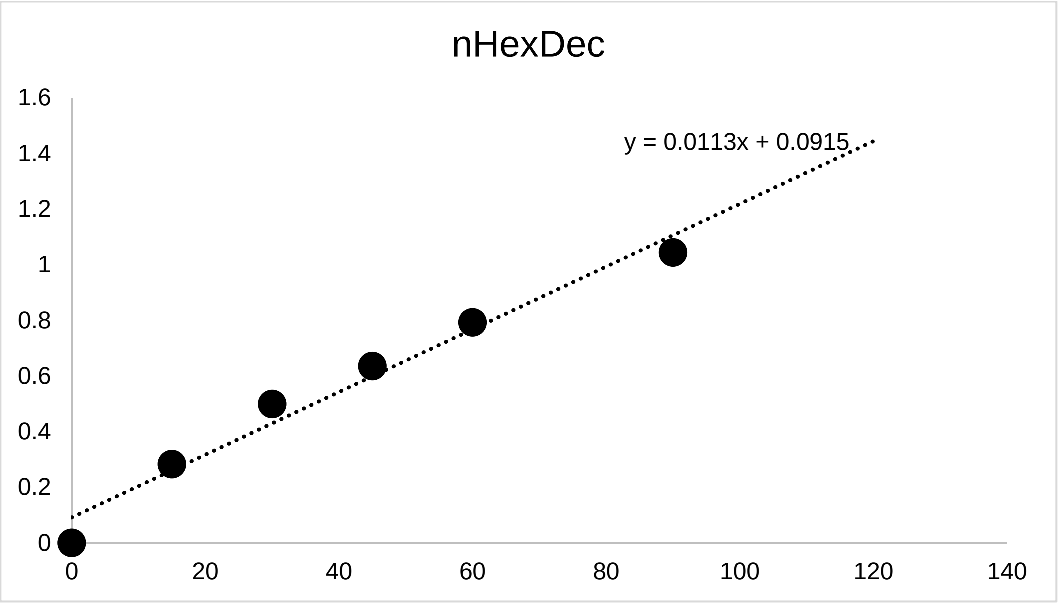

Figure S22: Representative homopolymerization kinetics, nHexDec. Time (seconds) vs ln(M_0/M_t).

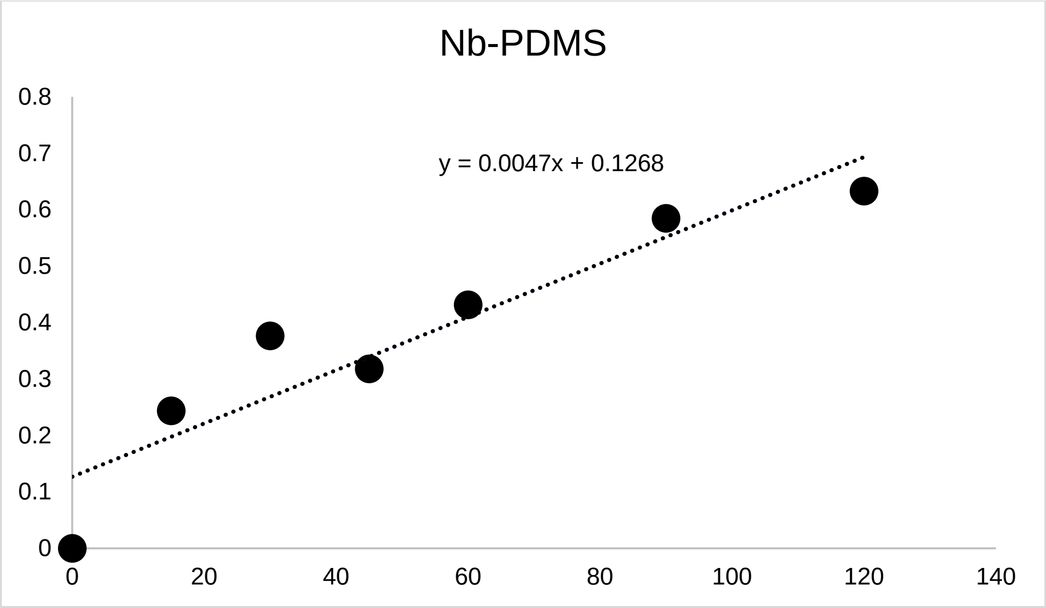

Figure S23: Representative homopolymerization kinetics, PDMS. Time (seconds) vs ln(M_0/M_t).

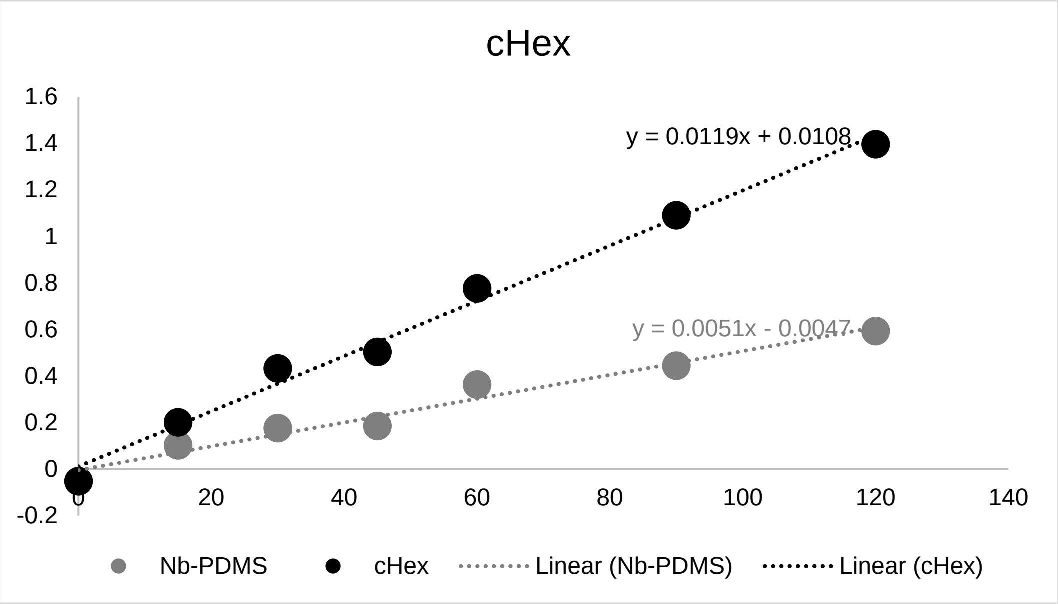

Figure S24: Representative copolymerization kinetics, cHex. Time (seconds) vs ln(M_0/M_t).

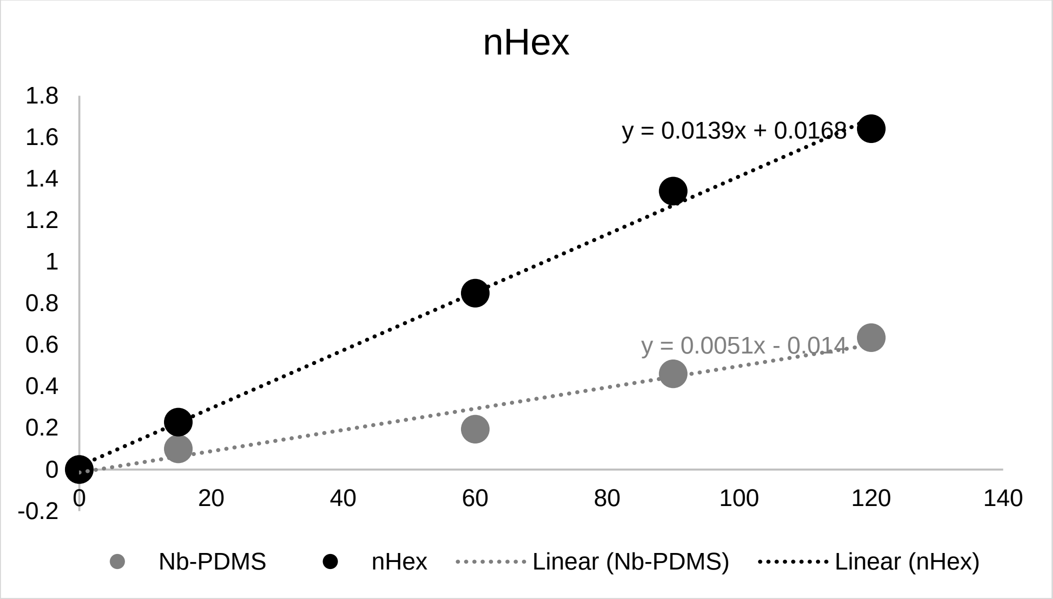

Figure S25: Representative copolymerization kinetics, nHex. Time (seconds) vs ln(M_0/M_t).

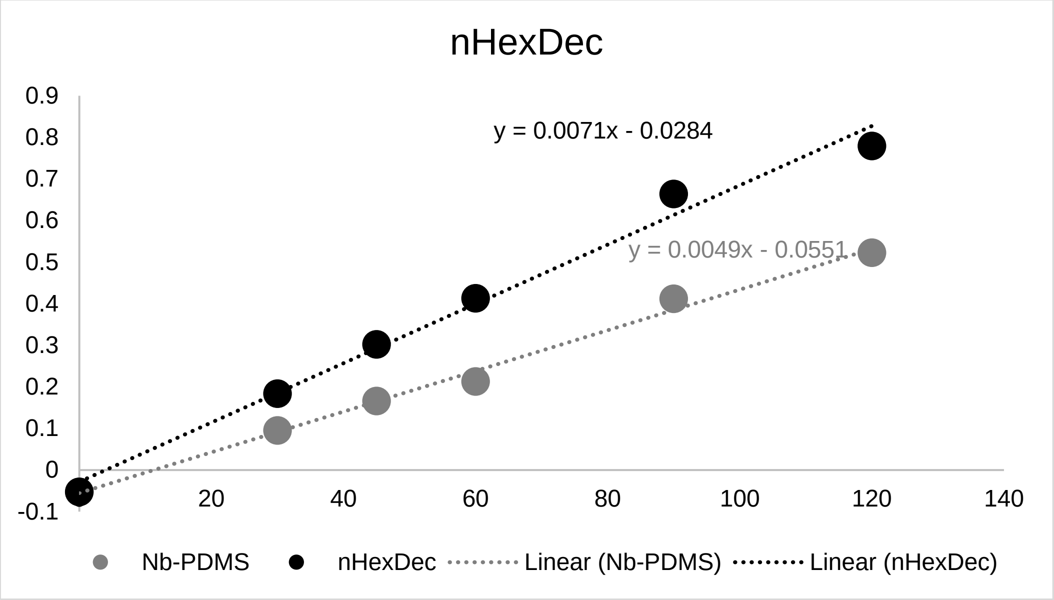

Figure S26: Representative copolymerization kinetics, nHexDec. Time (seconds) vs ln(M_0/M_t).

Entry	M1	M2	[M1] (mM)	[M2] (mM)	k_{22} (M^{-1} s^{-1})	k_{21} (M^{-1} s^{-1})	k_{11} (M^{-1} s^{-1})	k_{12} (M^{-1} s^{-1})	r_1	r_2	r_1* r_2
1	**Nb-PDMS**	**cHex**	2.64	2.64	286	169	89	129	0.69	1.69	1.16
2	**Nb-PDMS**	**nHex**	2.64	2.64	322	171	89	110	0.81	1.88	1.52
3	**Nb-PDMS**	**nHexDec**	2.64	2.64	214	100	89	100	0.89	2.14	1.90

Table S5: Reactivity Ratios and Copolymerization Kinetics Summary of Small Molecule Comonomers.

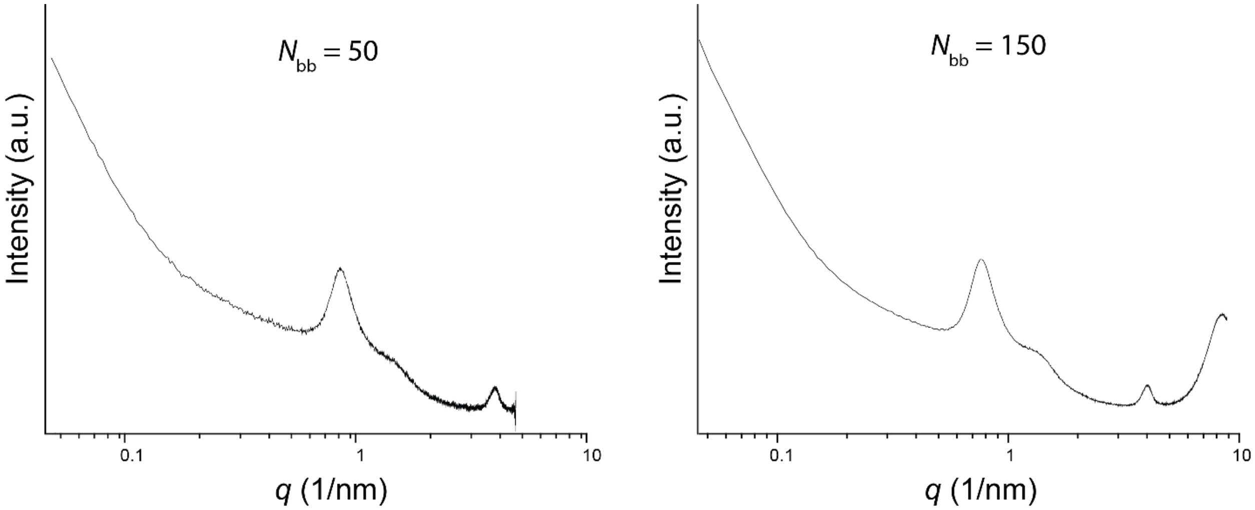

Figure S27: 1D SAXS Curves, PDMS Bottlebrush Homopolymers

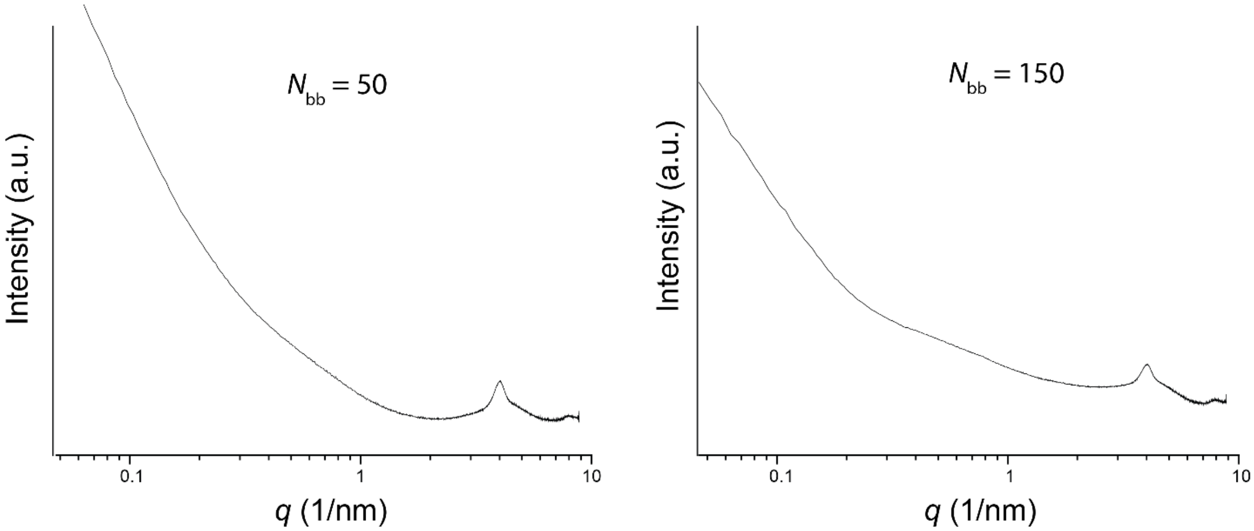

Figure S28: 1D SAXS Curves, cHex Homopolymers

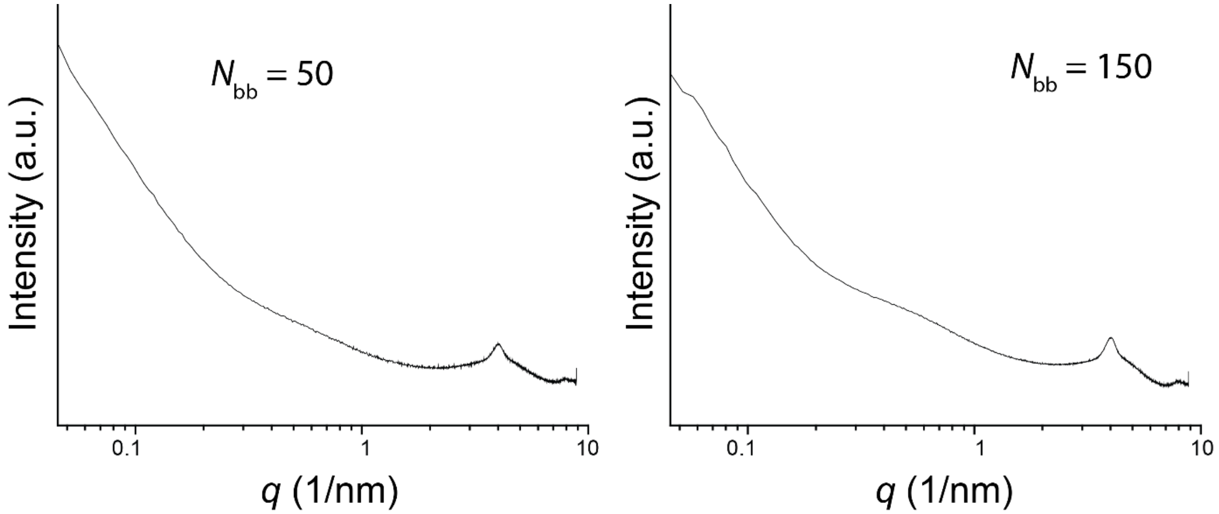

Figure S29: 1D SAXS Curves, nHex Homopolymers

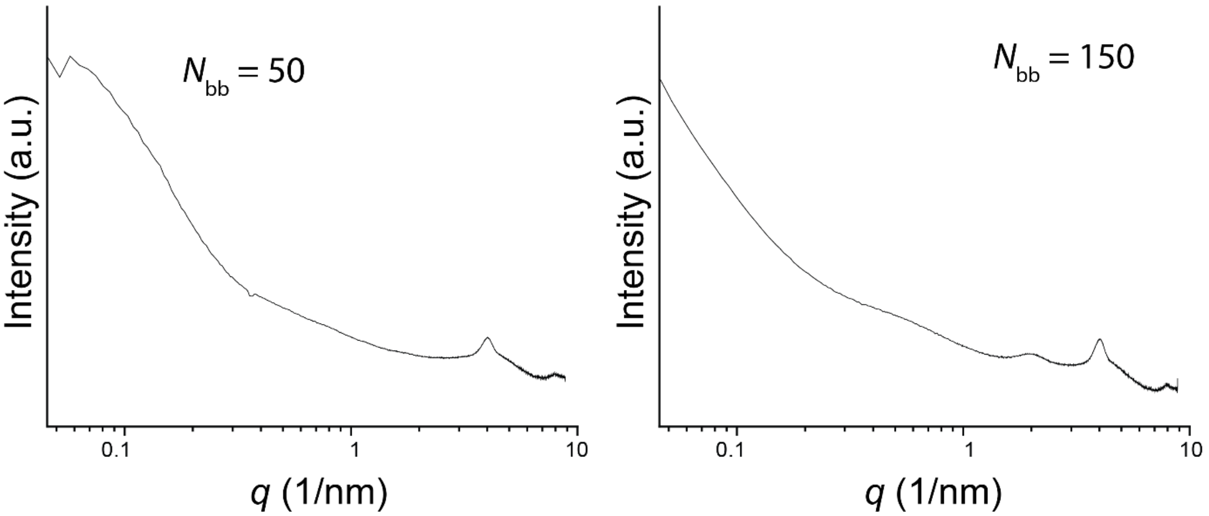

Figure S30: 1D SAXS Curves, nHexDec Homopolymers

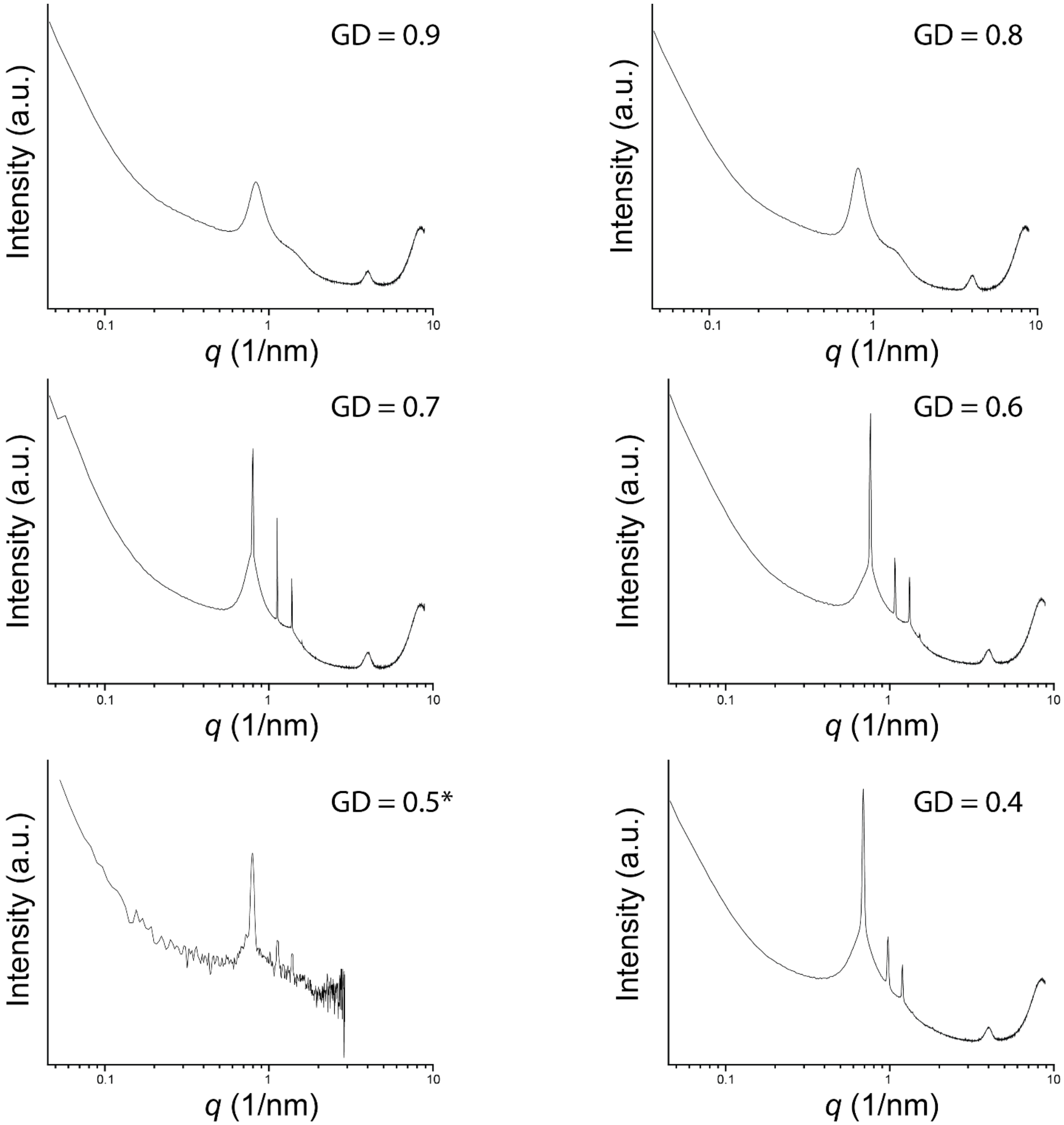

Figure S31: 1D SAXS Curves, N_{bb} = 50, *statistical,* cHex copolymers

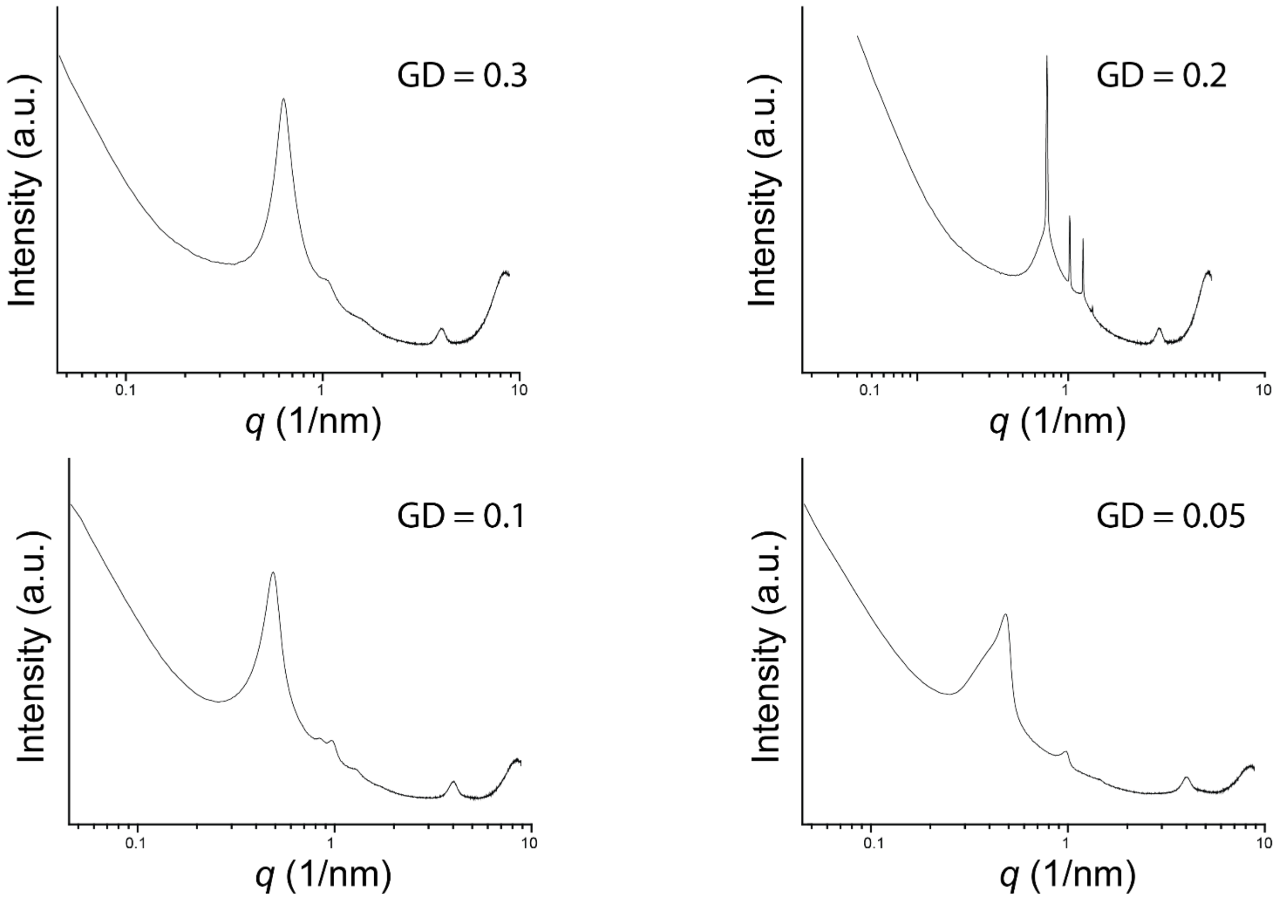

Figure S32: 1D SAXS Curves, N_{bb} = 50, *statistical,* cHex copolymers

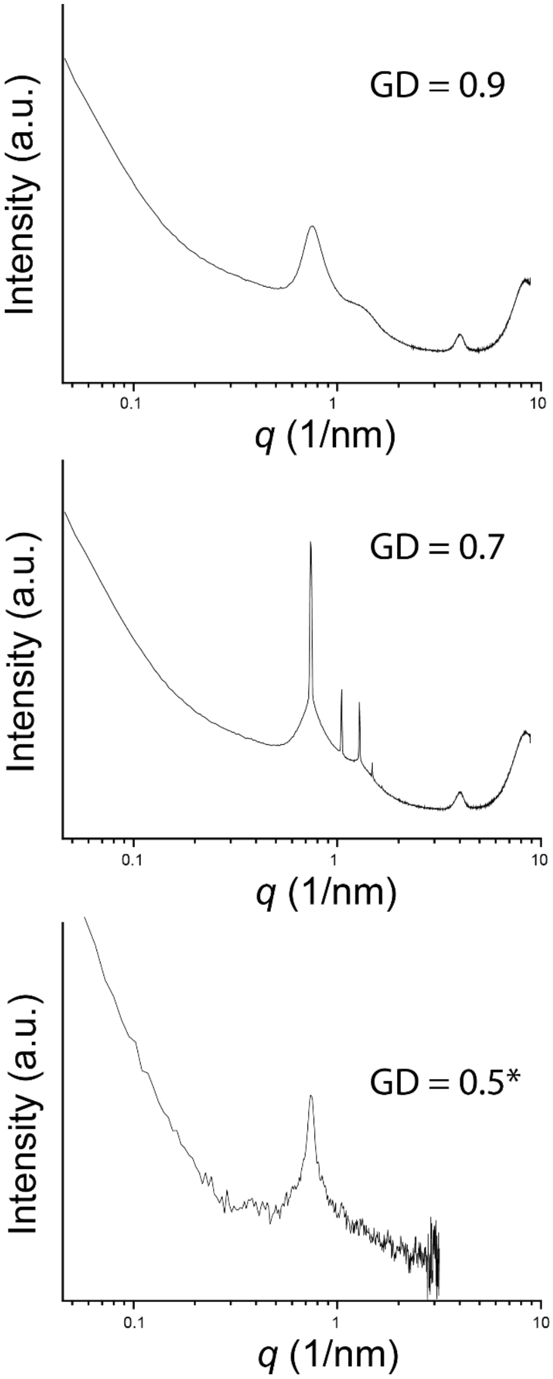

Figure S33: 1D SAXS Curves, N_{bb} = 150, *statistical,* cHex copolymers

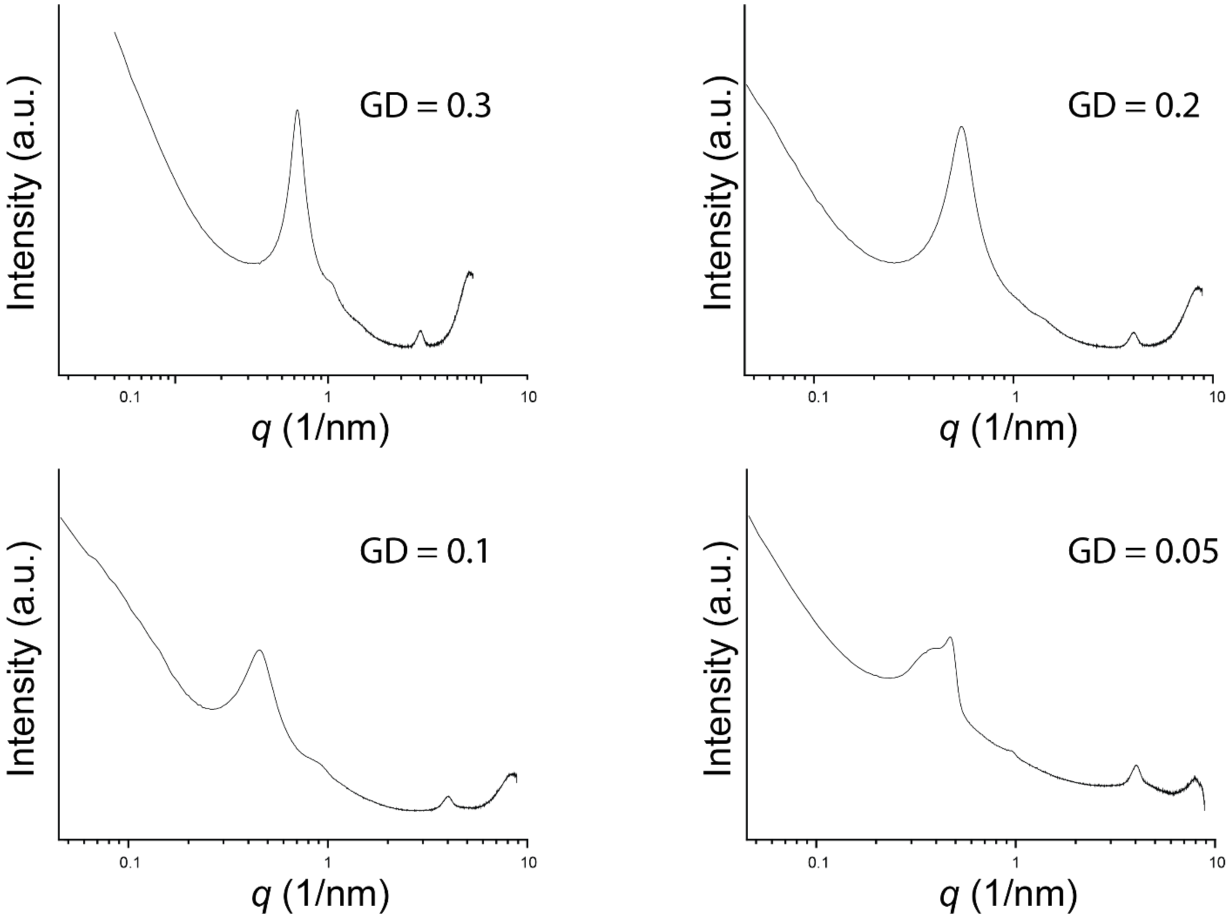

Figure S34: 1D SAXS Curves, N_{bb} = 150, *statistical,* cHex copolymers

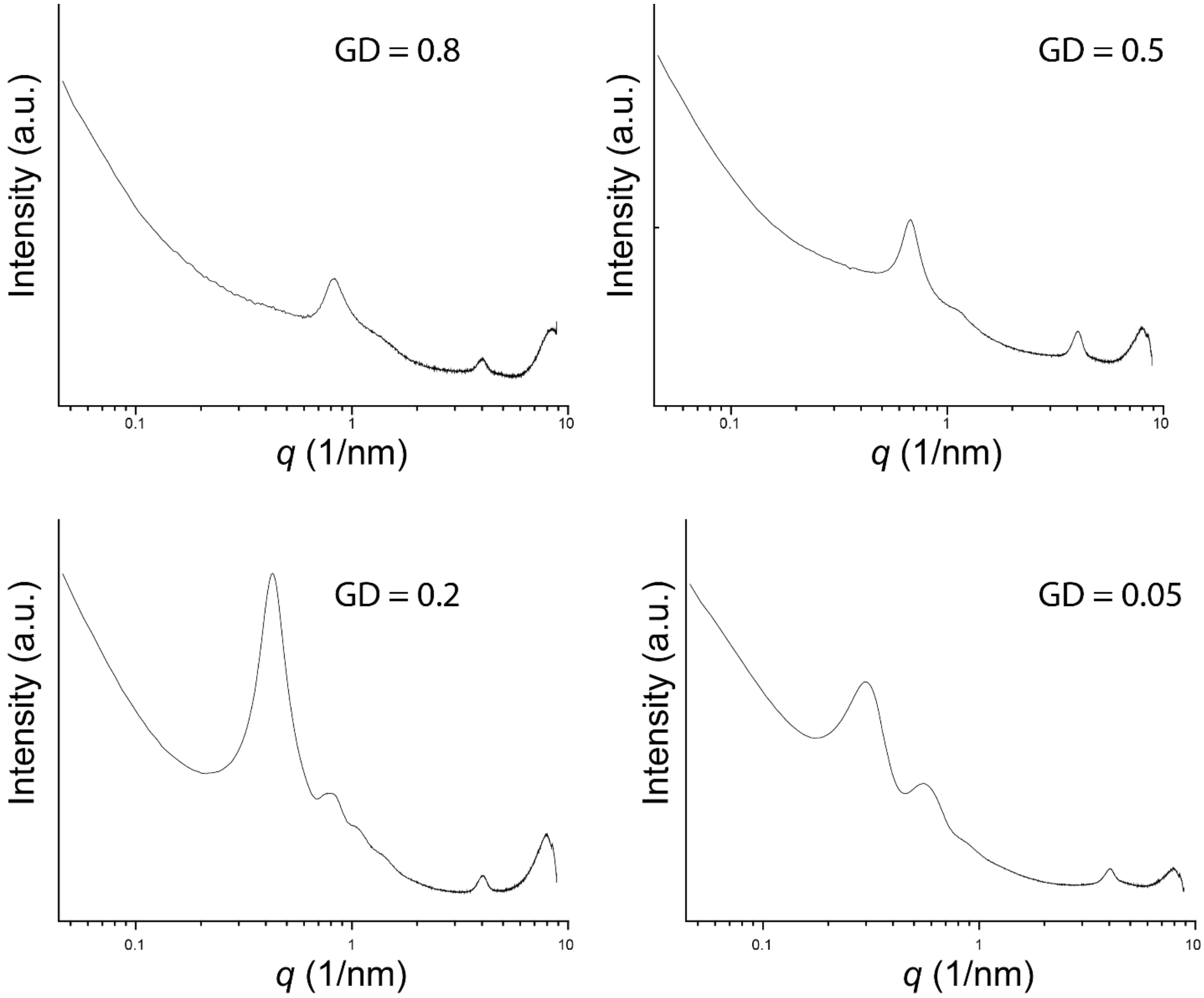

Figure S35: 1D SAXS Curves, N_{bb} = 50, *block,* cHex copolymers

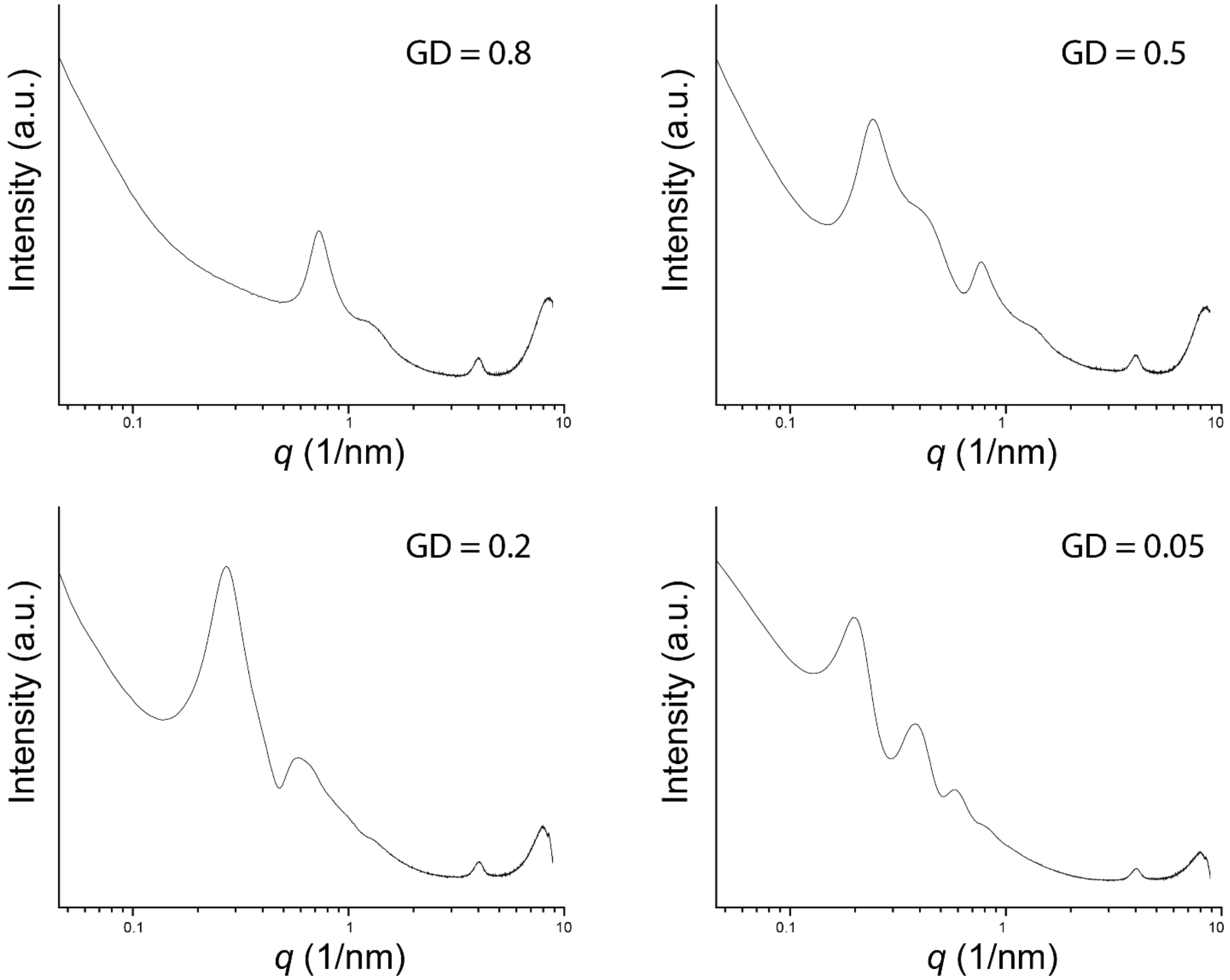

Figure S36: 1D SAXS Curves, N_{bb} = 150, *block,* cHex copolymers

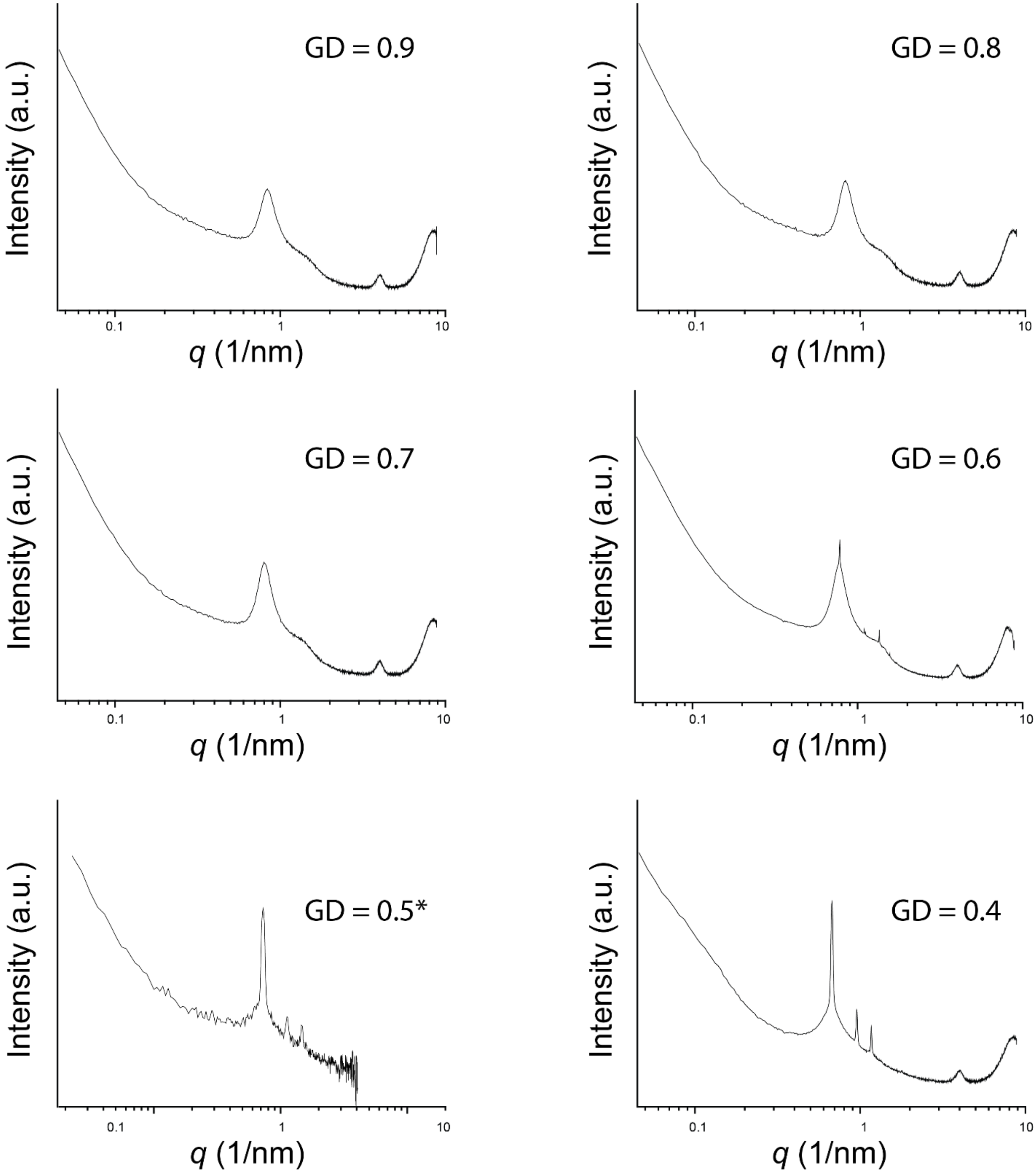

Figure S37: 1D SAXS Curves, N_{bb} = 50, *statistical,* nHex copolymers

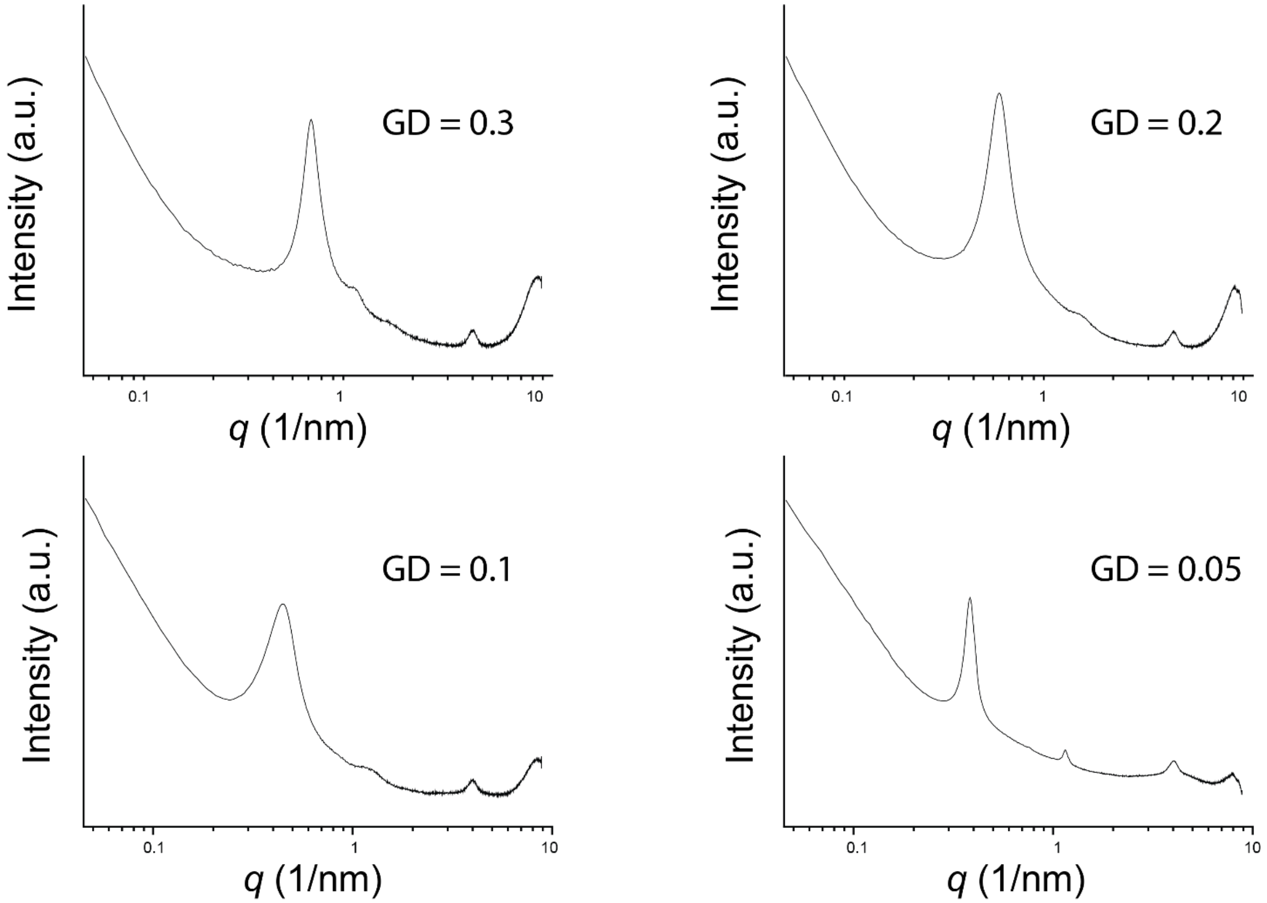

Figure S38: 1D SAXS Curves, N_{bb} = 50, *statistical,* nHex copolymers

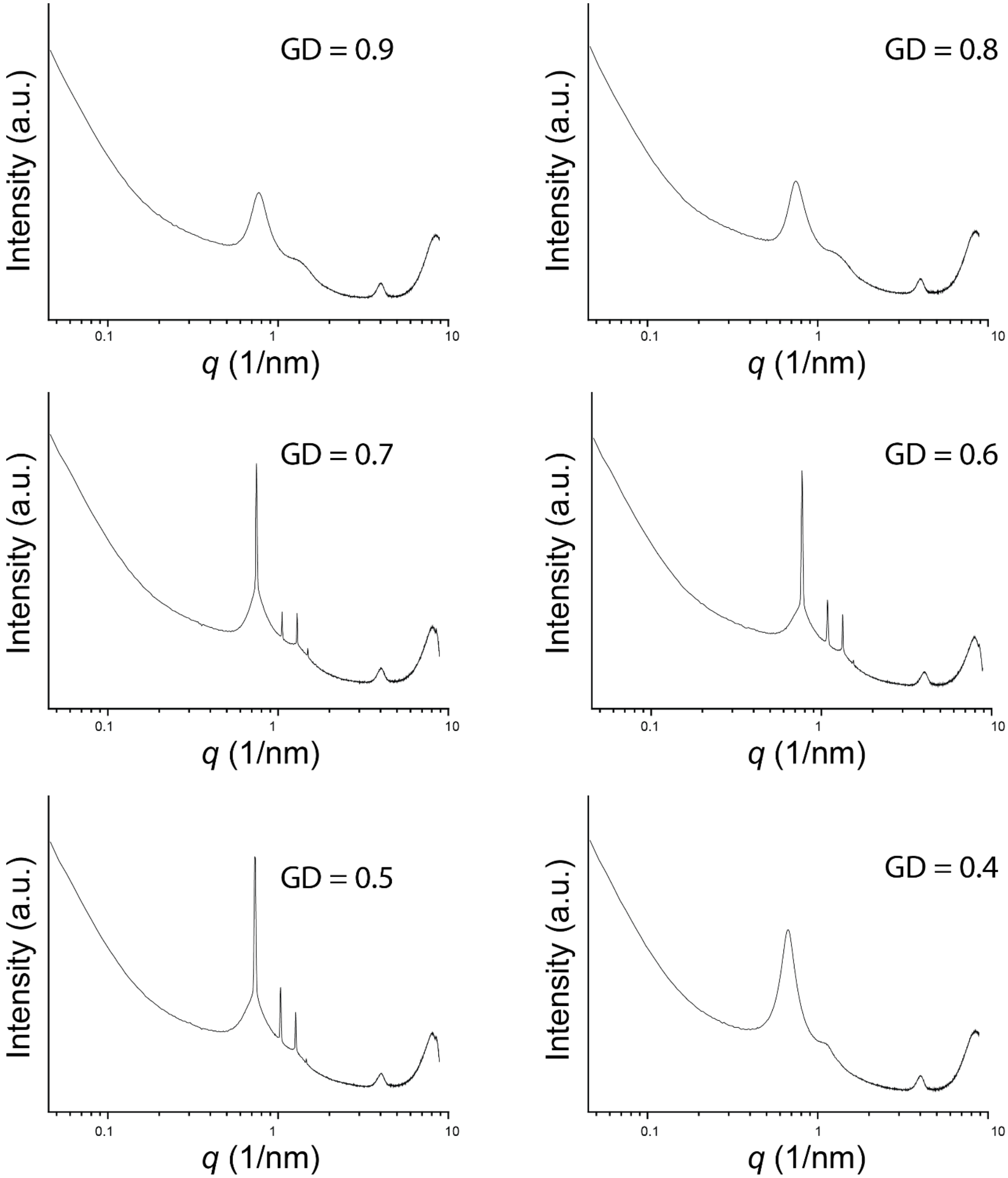

Figure S39: 1D SAXS Curves, N_{bb} = 150, *statistical,* nHex copolymers

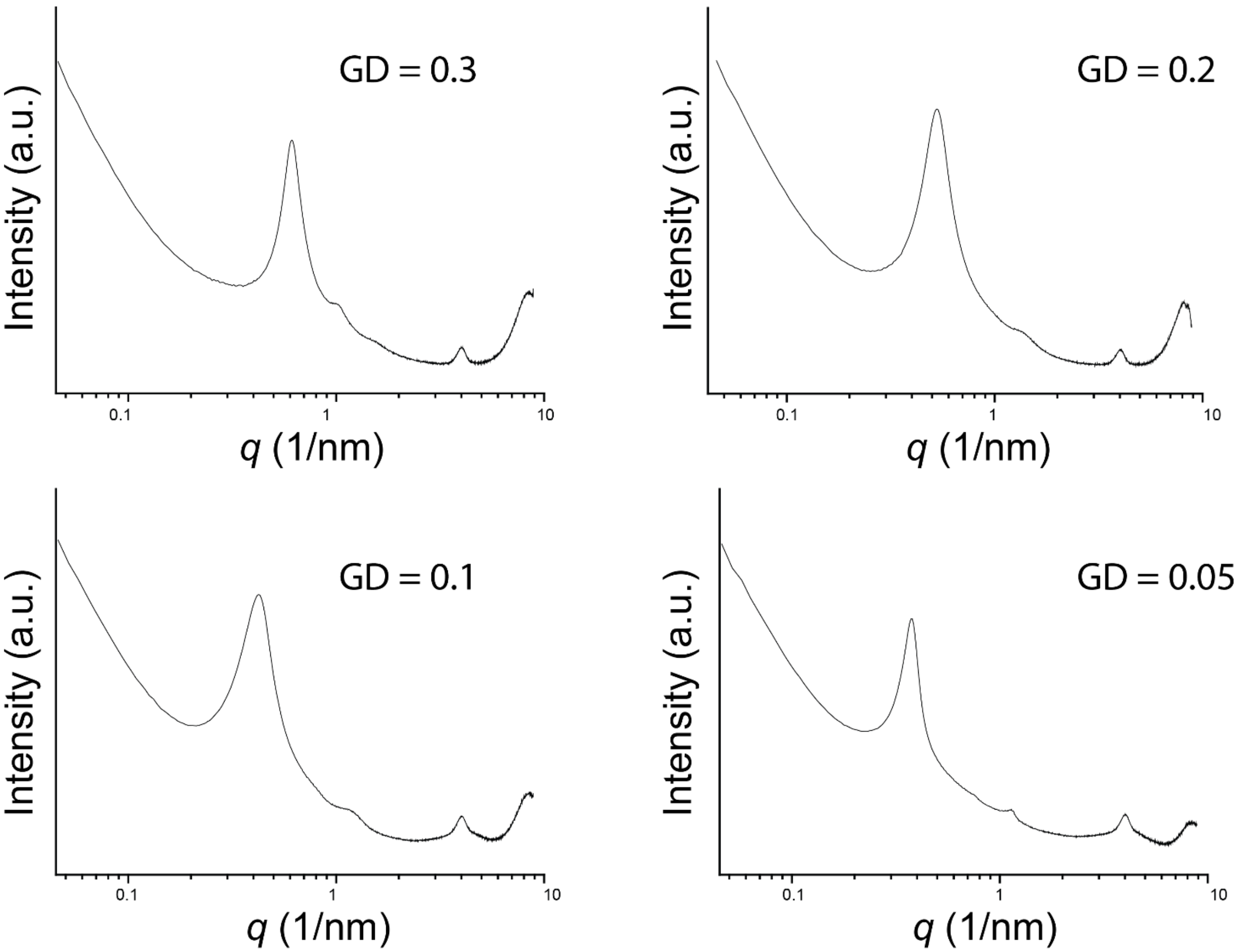

Figure S40: 1D SAXS Curves, N_{bb} = 150, *statistical,* nHex copolymers

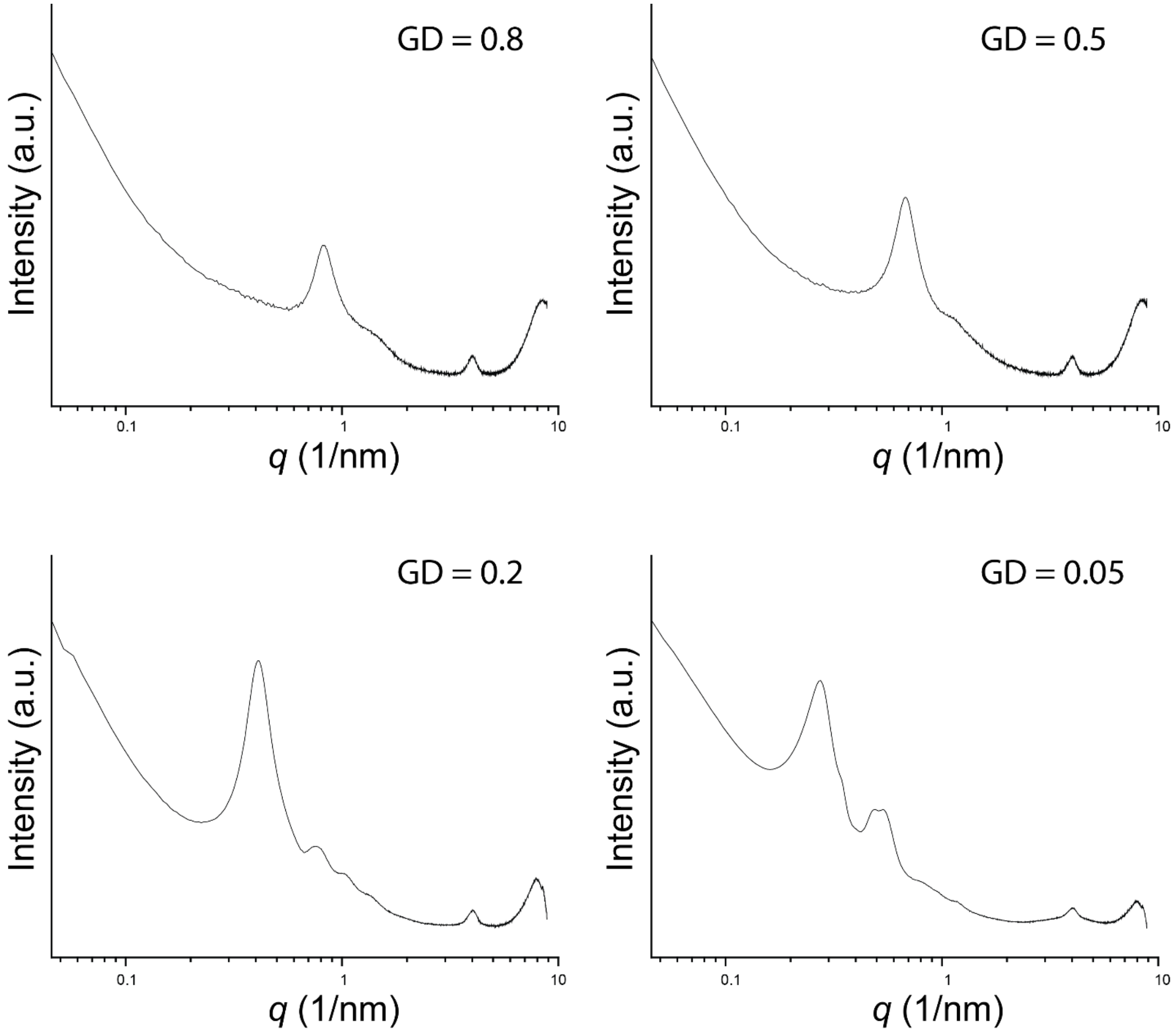

Figure S41: 1D SAXS Curves, N_{bb} = 50, *block,* nHex copolymers

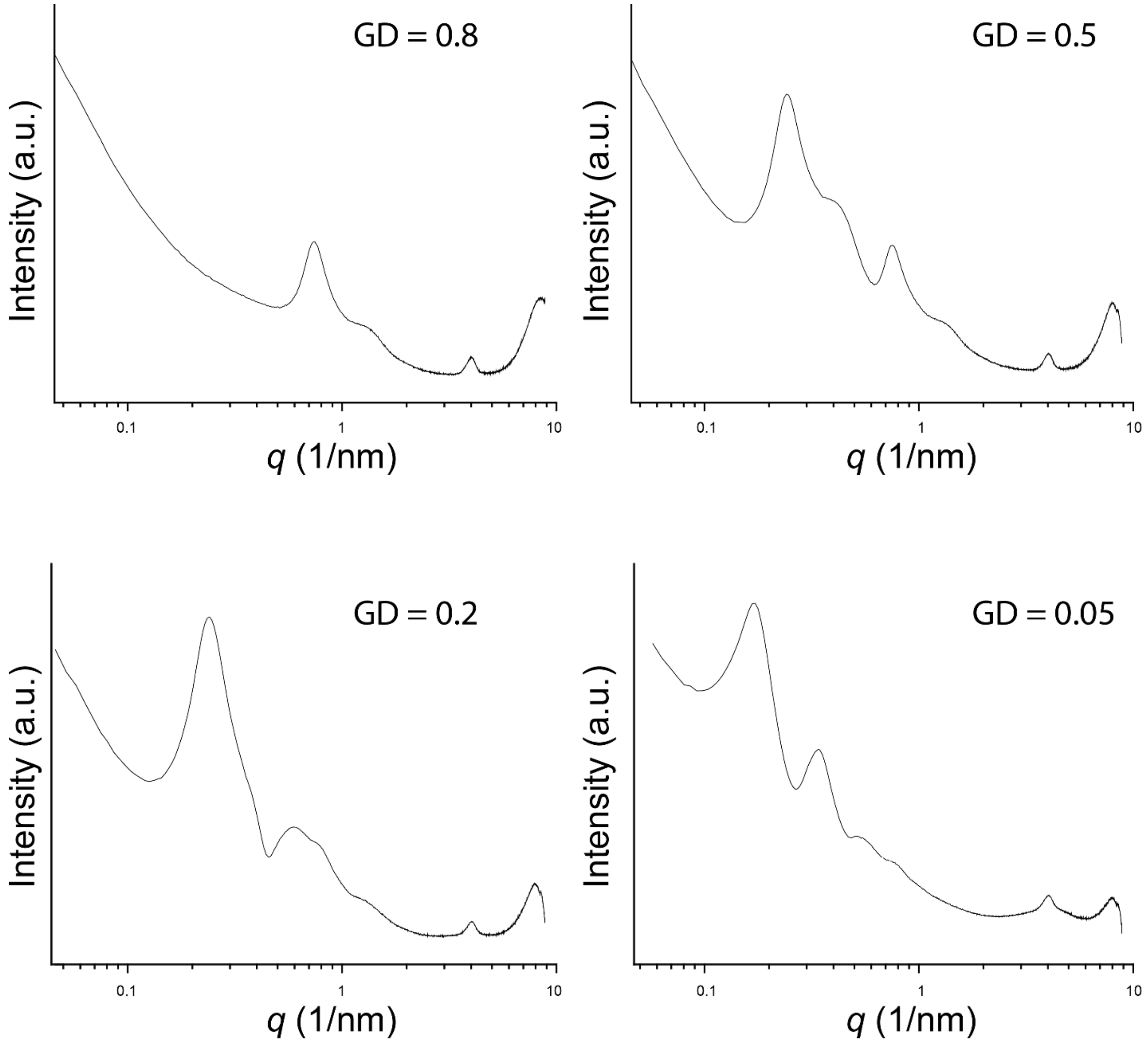

Figure S42: 1D SAXS Curves, N_{bb} = 150, *block,* nHex copolymers

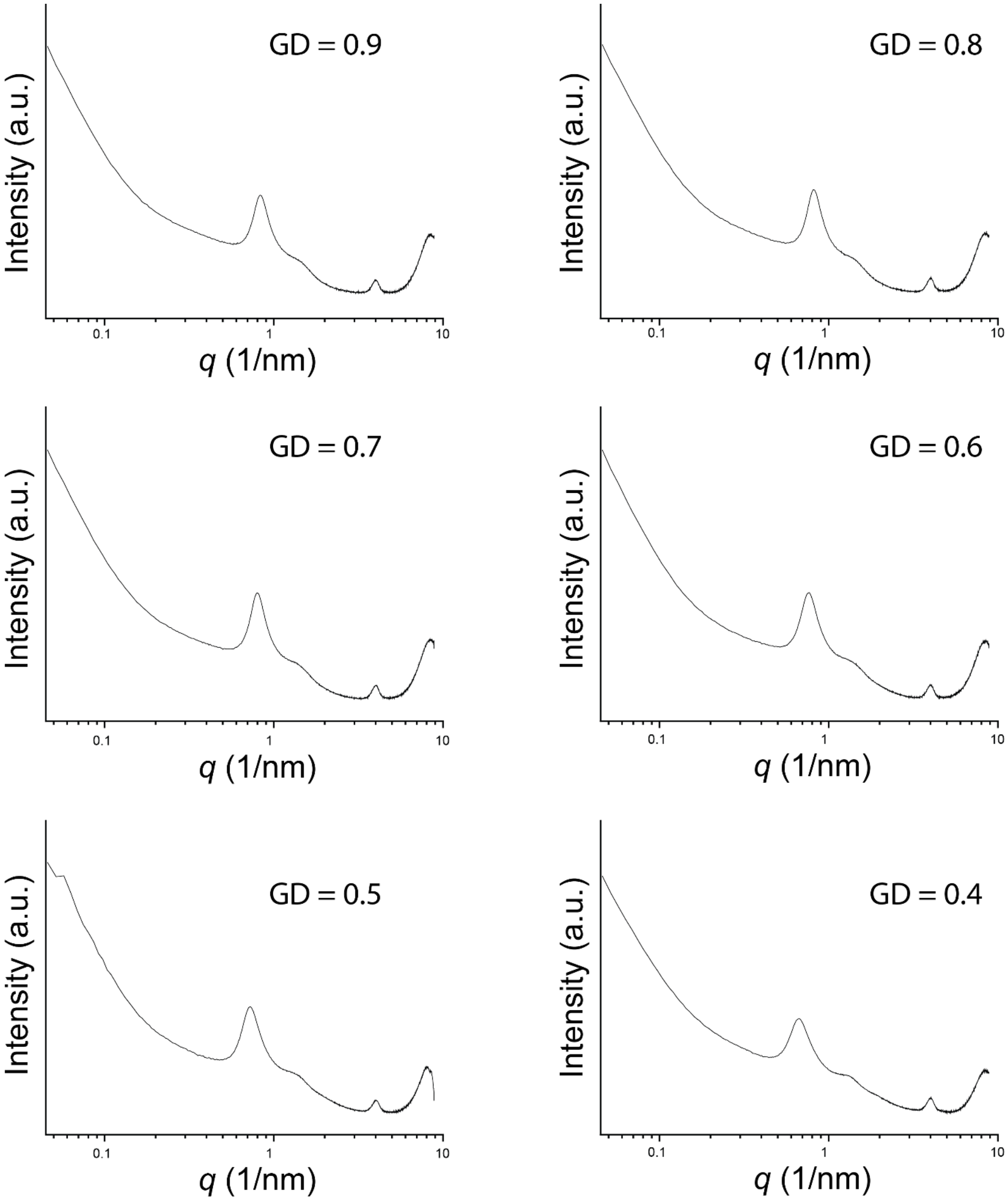

Figure S43: 1D SAXS Curves N_{bb} = 50, *statistical* nHexDec Copolymers

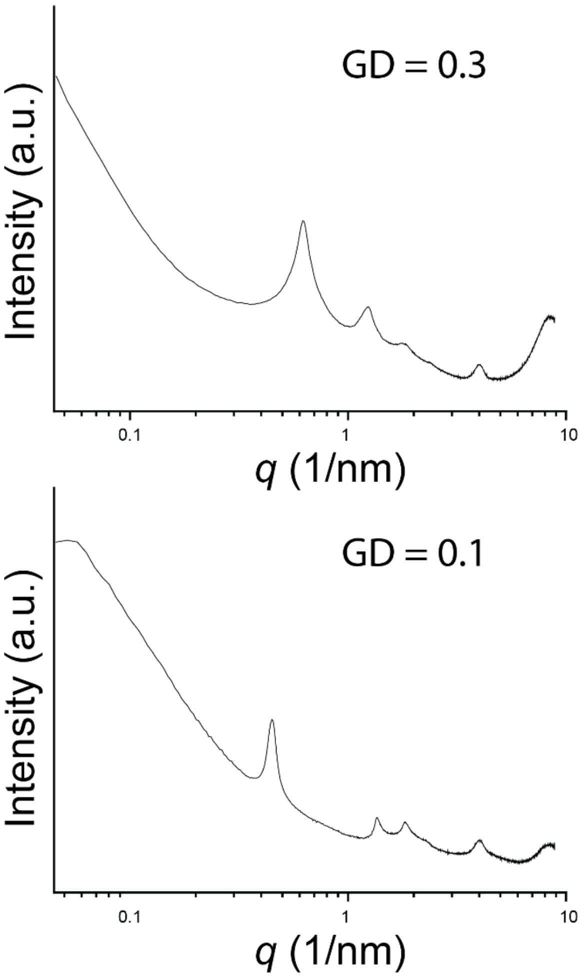

Figure S44: 1D SAXS Curves N_{bb} = 50, *statistical* nHexDec Copolymers

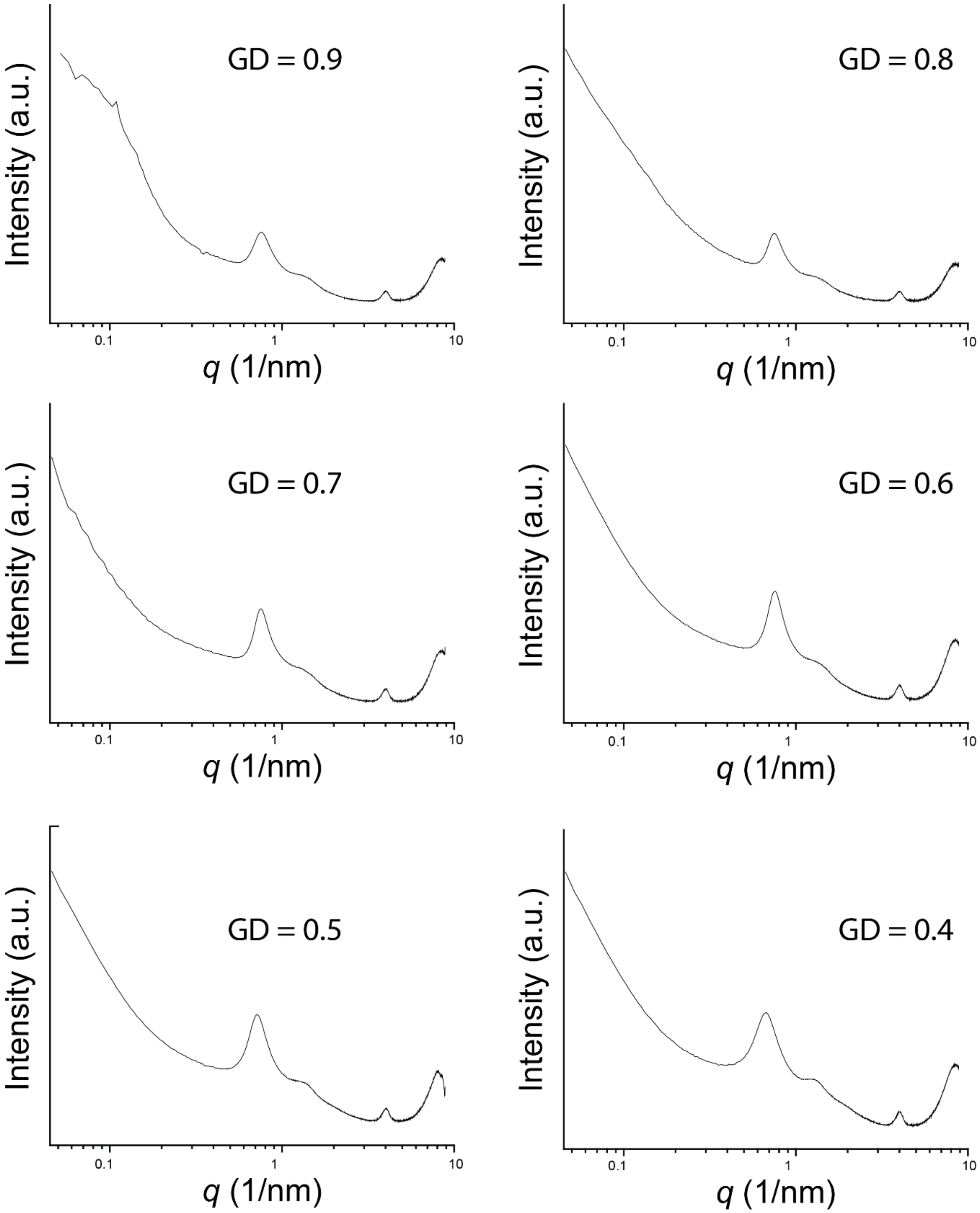

Figure S45: 1D SAXS Curves N_{bb} = 150, *statistical* nHexDec Copolymers

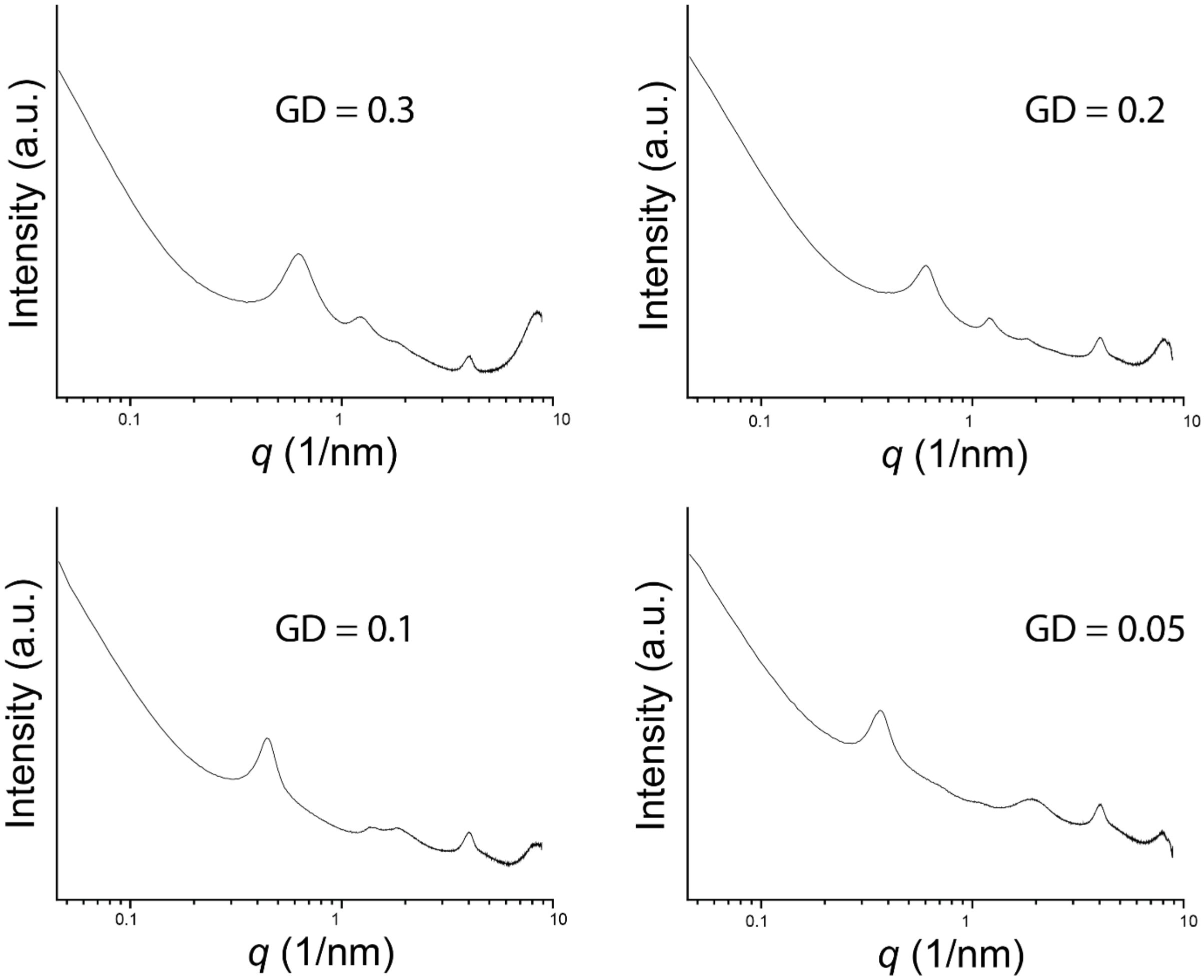

Figure S46: 1D SAXS Curves N_{bb} = 150, *statistical* nHexDec Copolymers

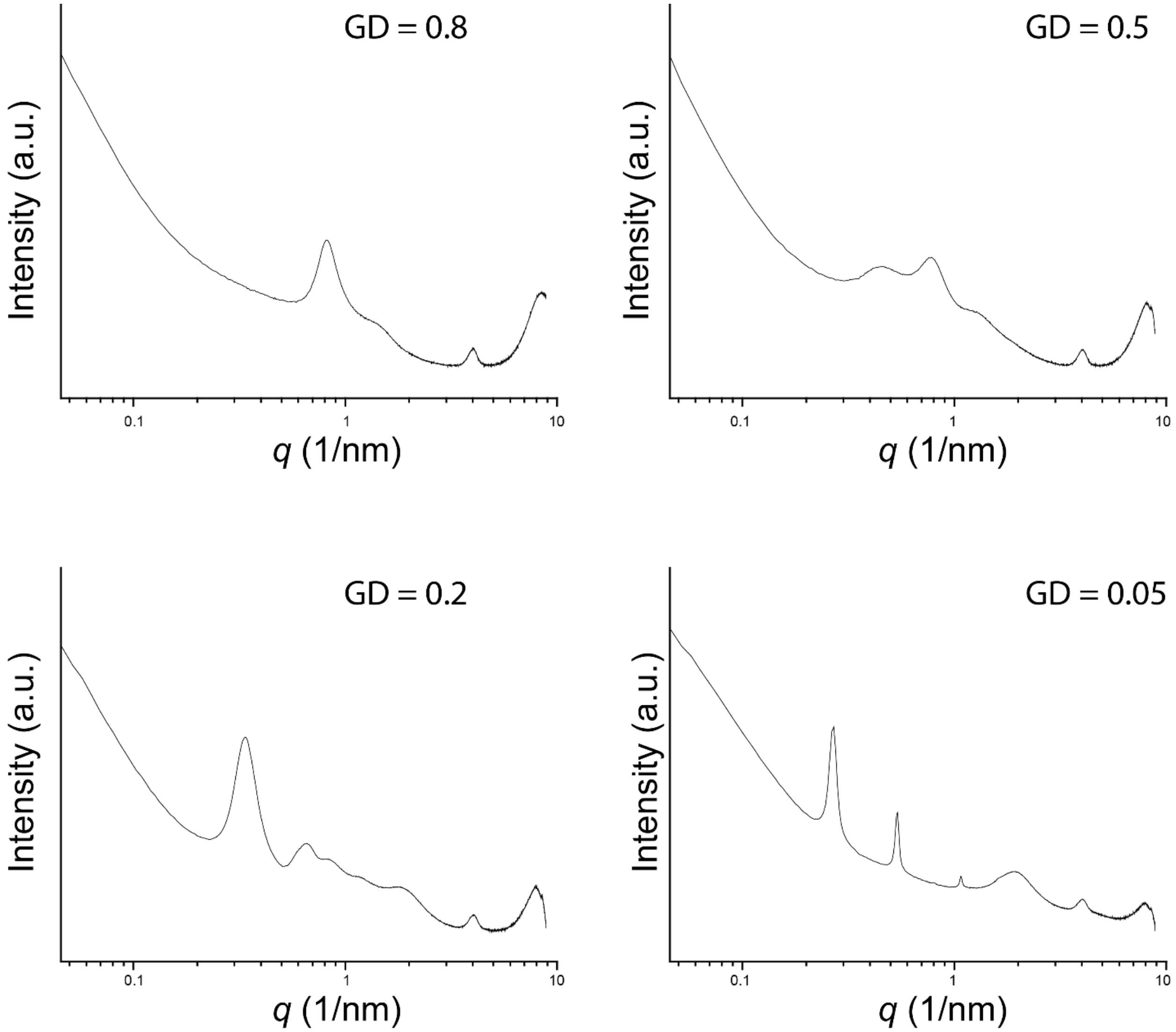

Figure S47: 1D SAXS Curves N_{bb} = 50, *block* nHexDec Copolymers

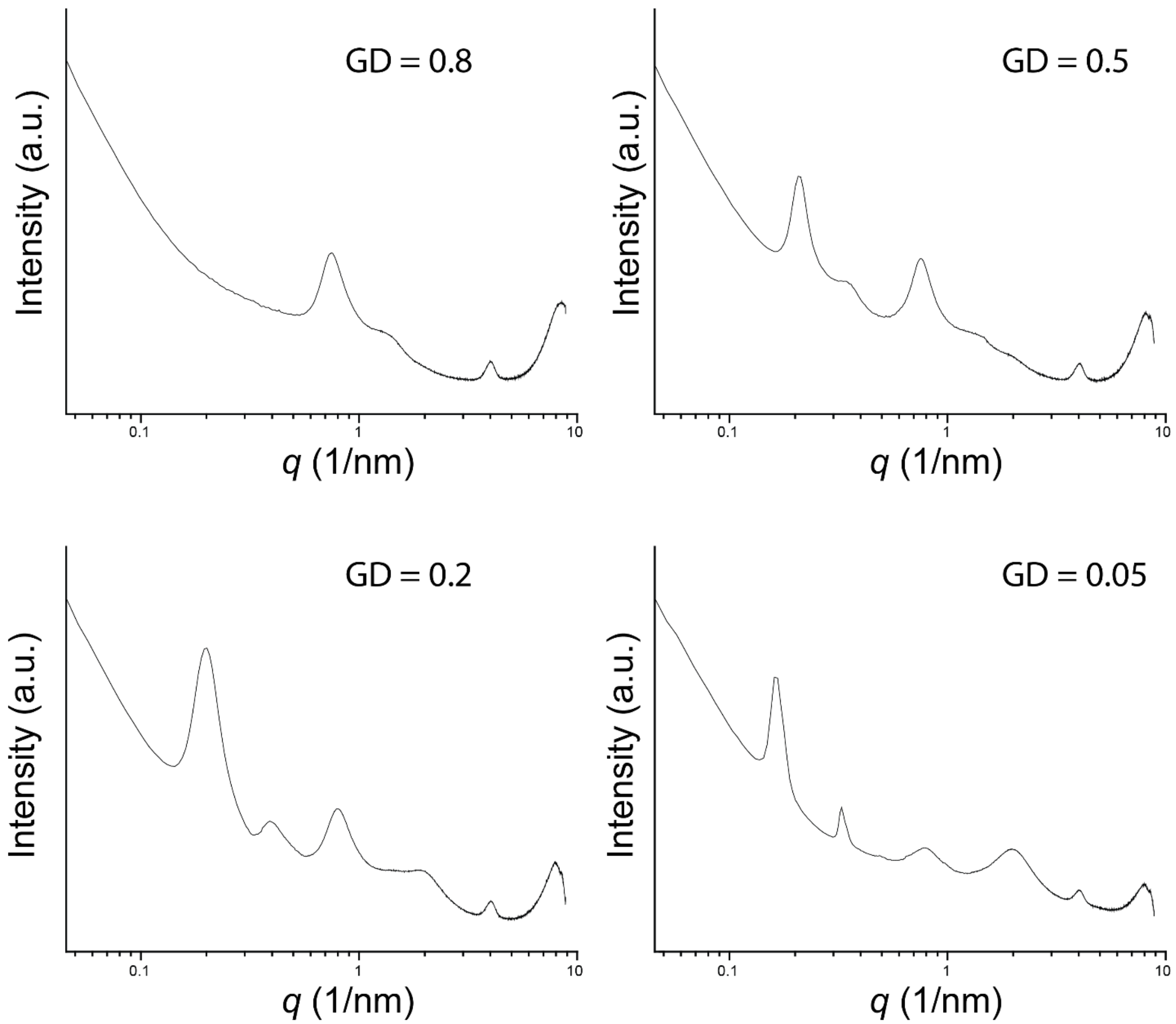

Figure S48: 1D SAXS Curves N_{bb} = 150, *block* nHexDec Copolymers

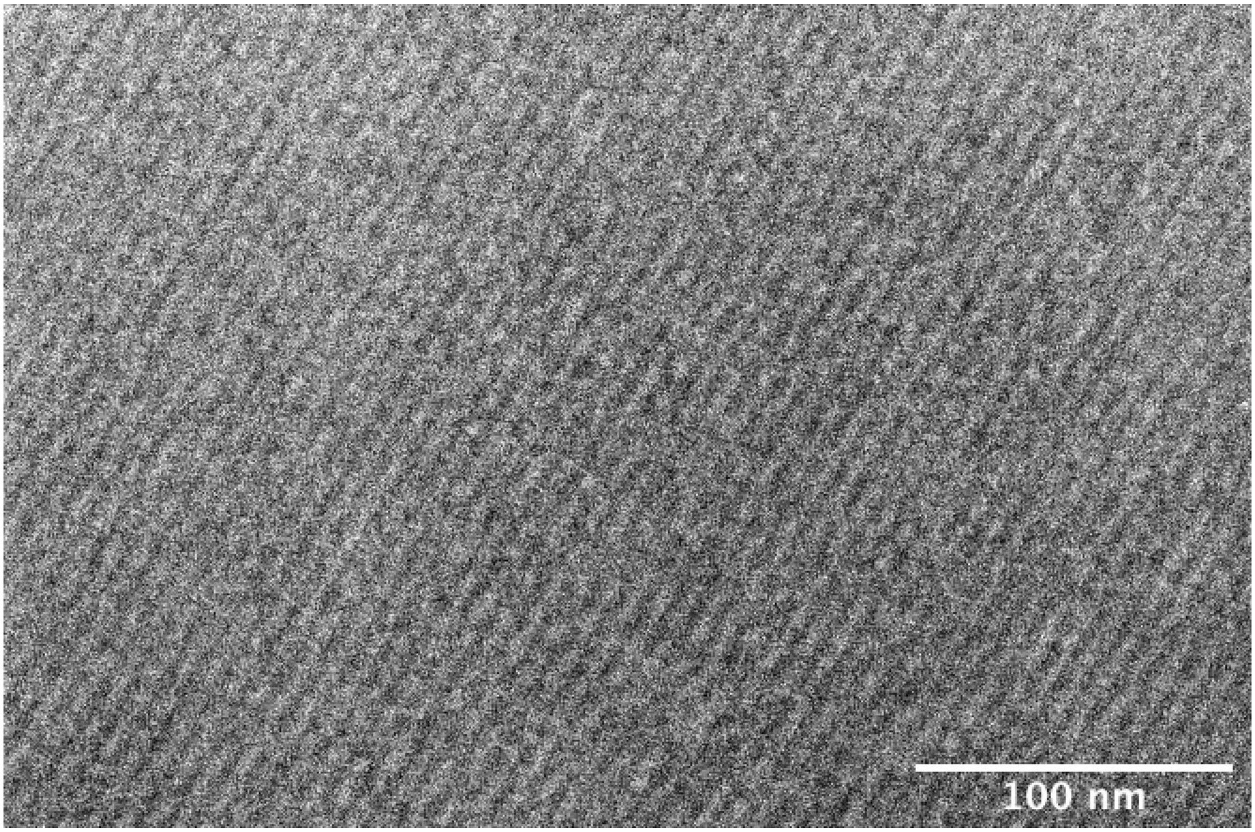

Figure S49: TEM – *stat*-cHex-50-0.6.

Excellent long-range ordering consistent with viewing from 200 plane. Line pattern emerges despite BCC spherical morphology detected *via* SAXS.

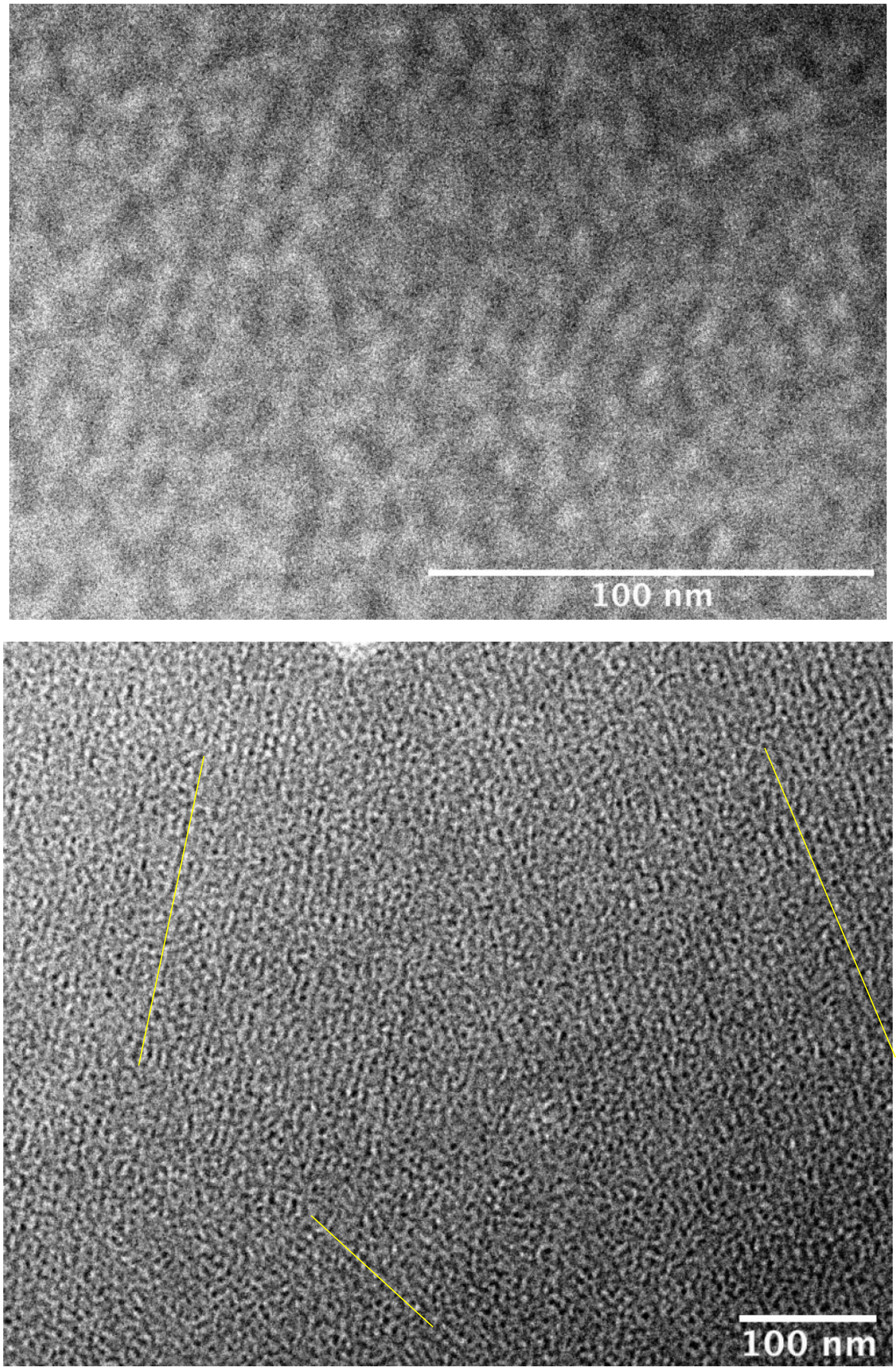

Figure S50: TEM – *stat*-nHex-50-0.6.

Top: Mixed phase BCC spheres in PDMS matrix.

Bottom: Long range order and near-micron grains visible when magnification is reduced. Yellow lines are present to guide the eye to grains of long-range order.

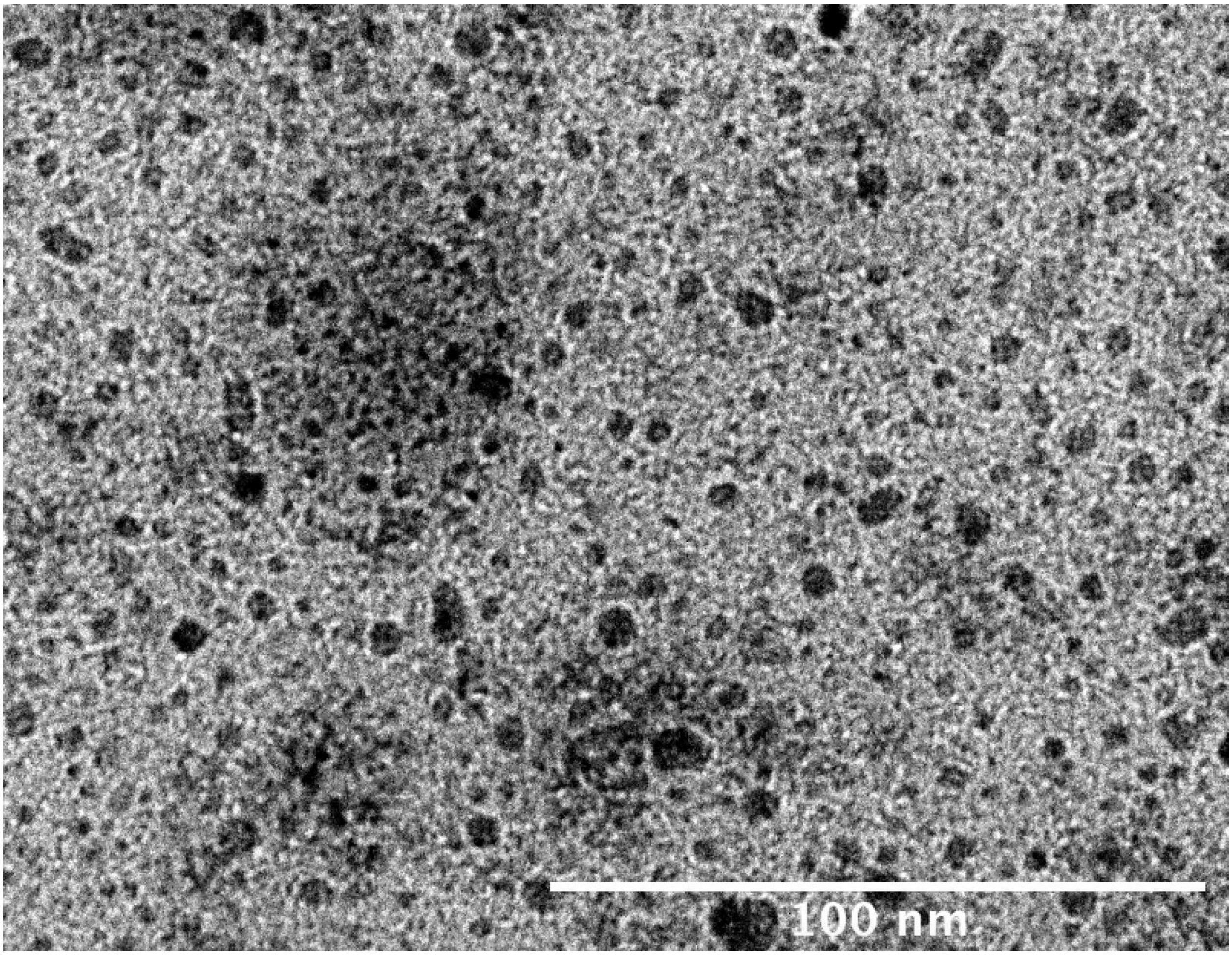

Figure S51: TEM – *stat*-cHex-50-0.2.

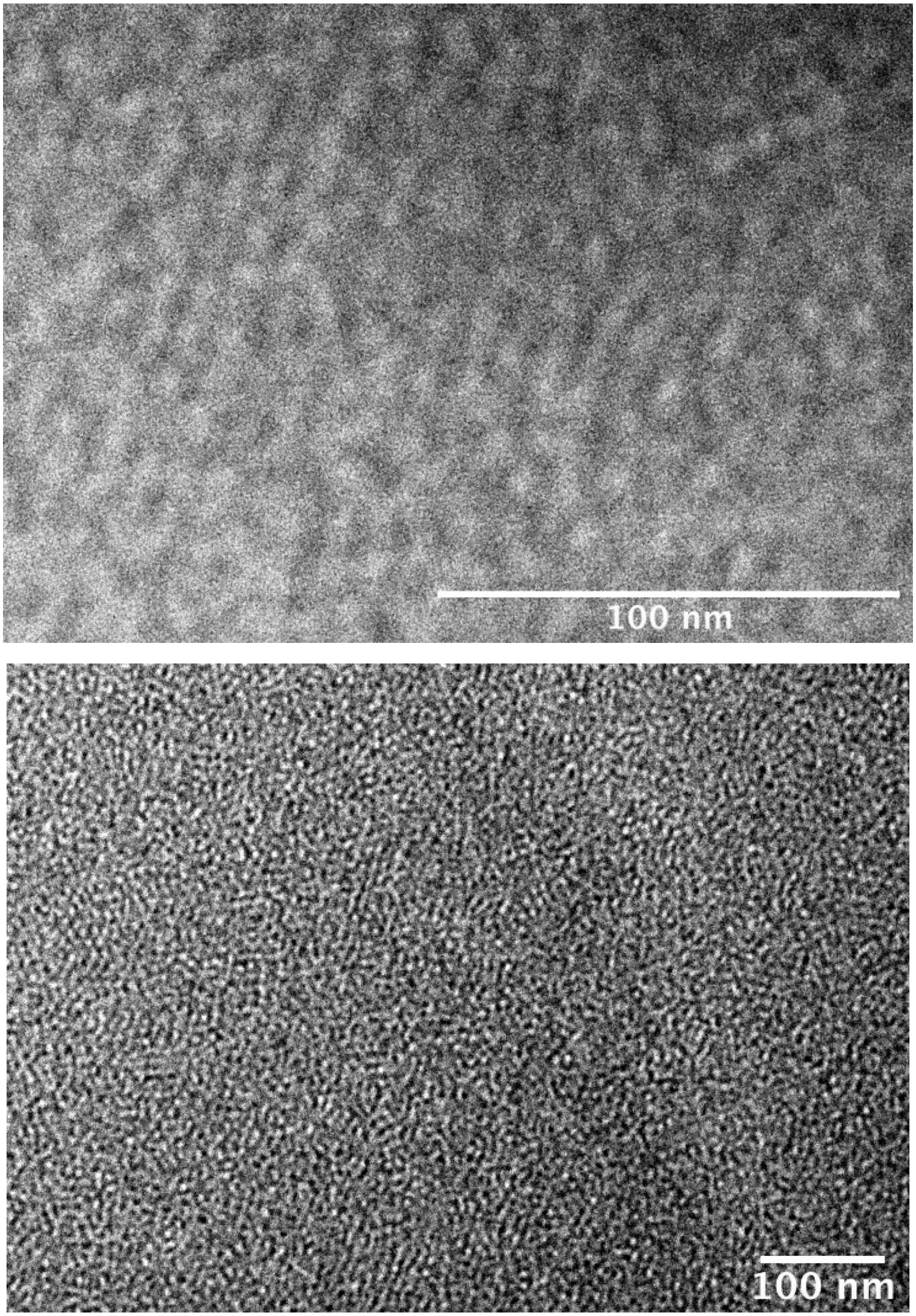

Figure S52: TEM – *stat*-nHex-150-0.6.

Top: BCC spheres in PDMS matrix.

Bottom: Some long-range order visible when magnification is reduced.

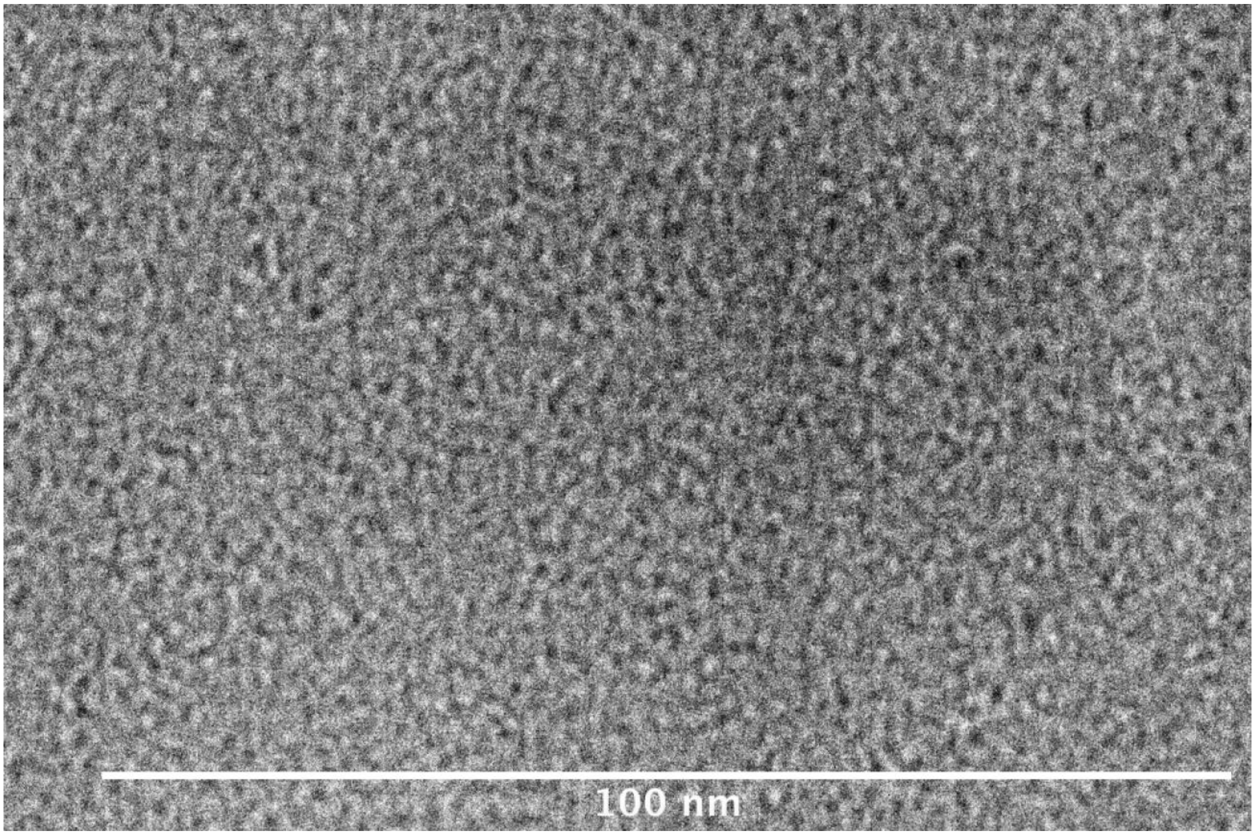

Figure S53: TEM – *stat*-nHex-50-0.2.

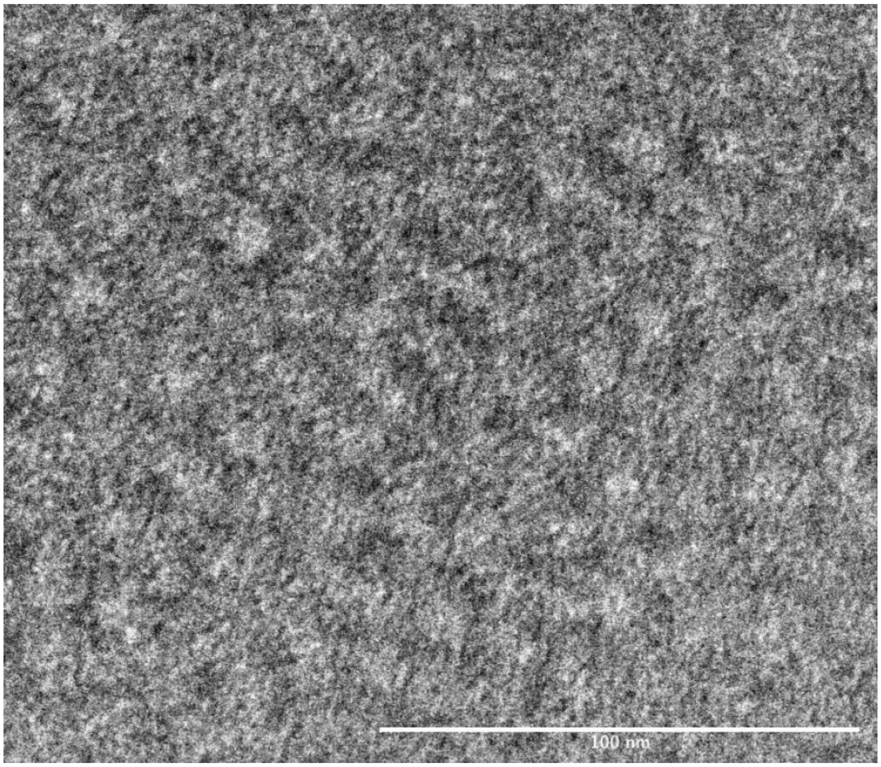

Figure S54: TEM – *stat*-nHex-150-0.1.

Cylinders with ~15 nm domain spacing, consistent with SAXS.

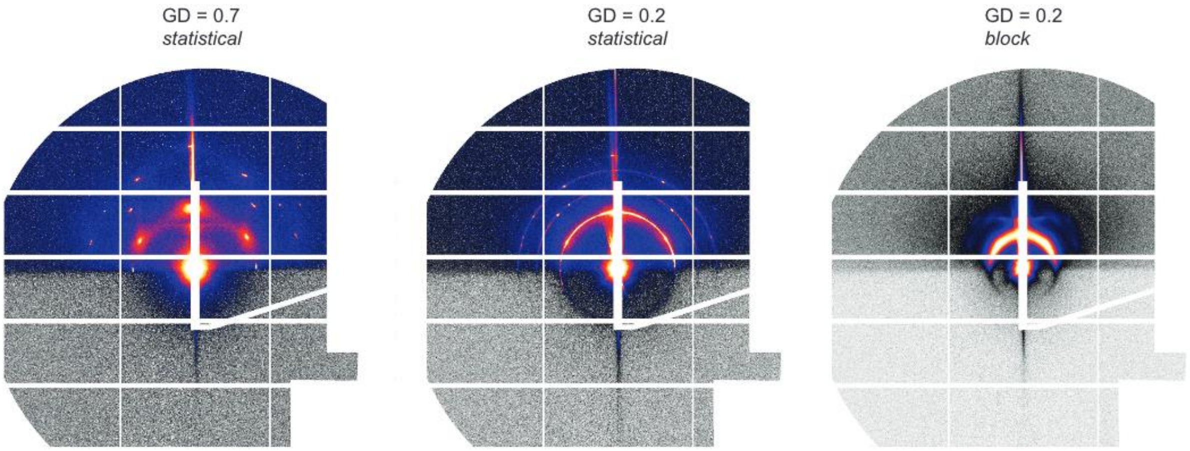

Figure S55: Raw 2D GISAXS – XX-cHex-50-XX.

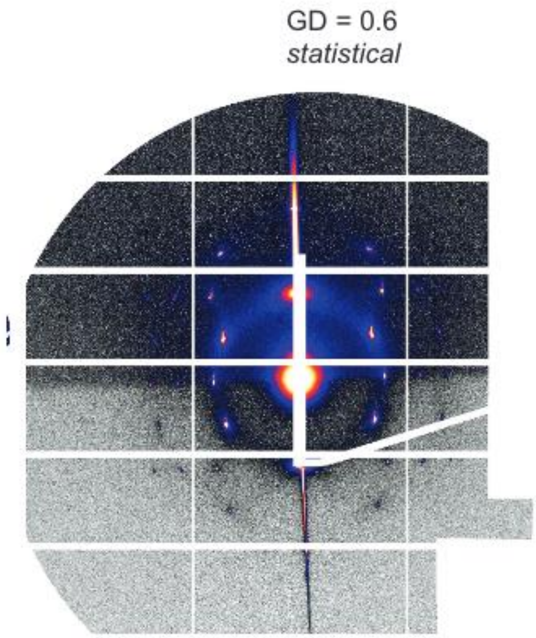

Figure S56: Raw 2D GISAXS – XX-cHex-150-XX.

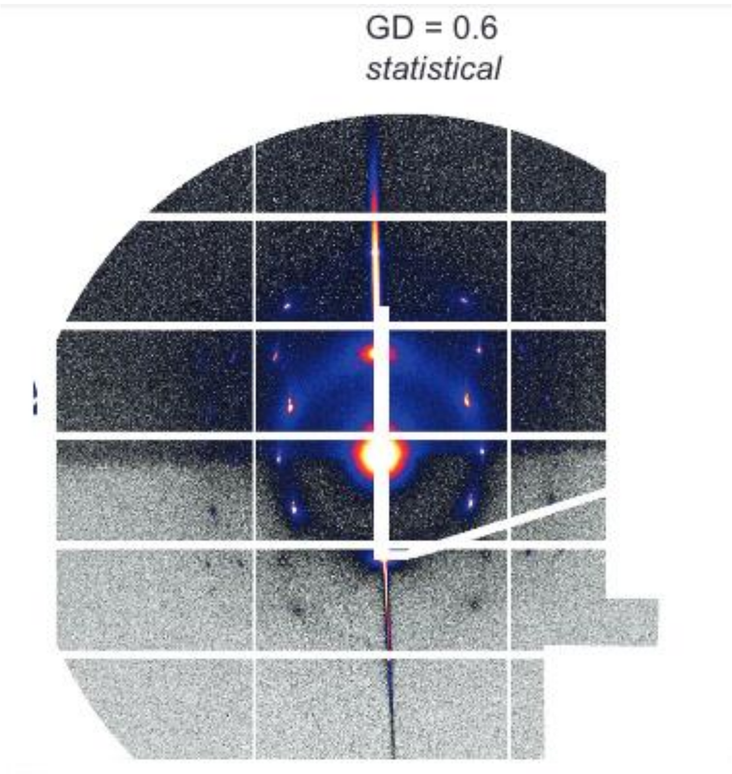

Figure S57: Raw 2D GISAXS – XX-nHex-50-XX.

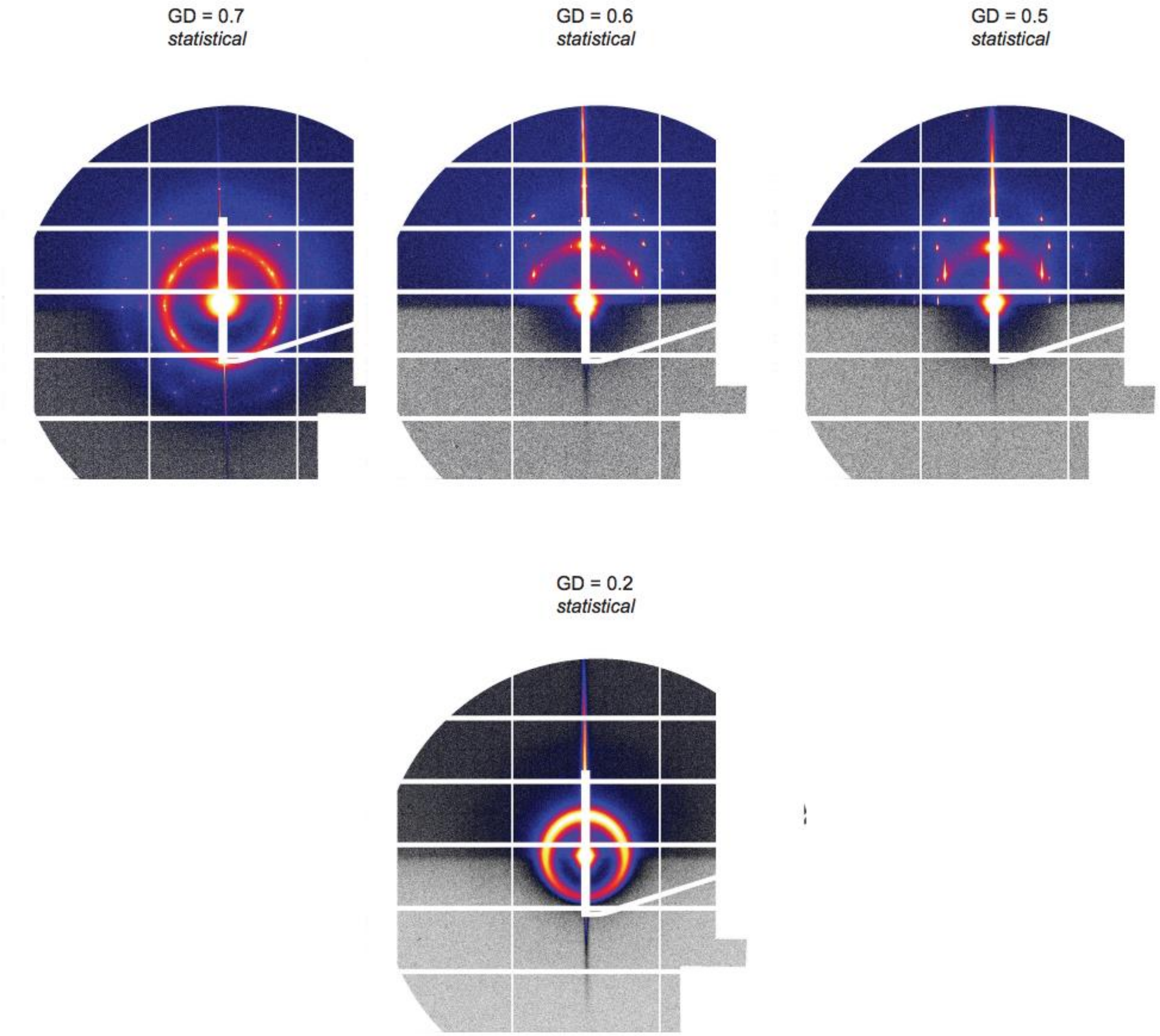

Figure S58: Raw 2D GISAXS – XX-nHex-150-XX.

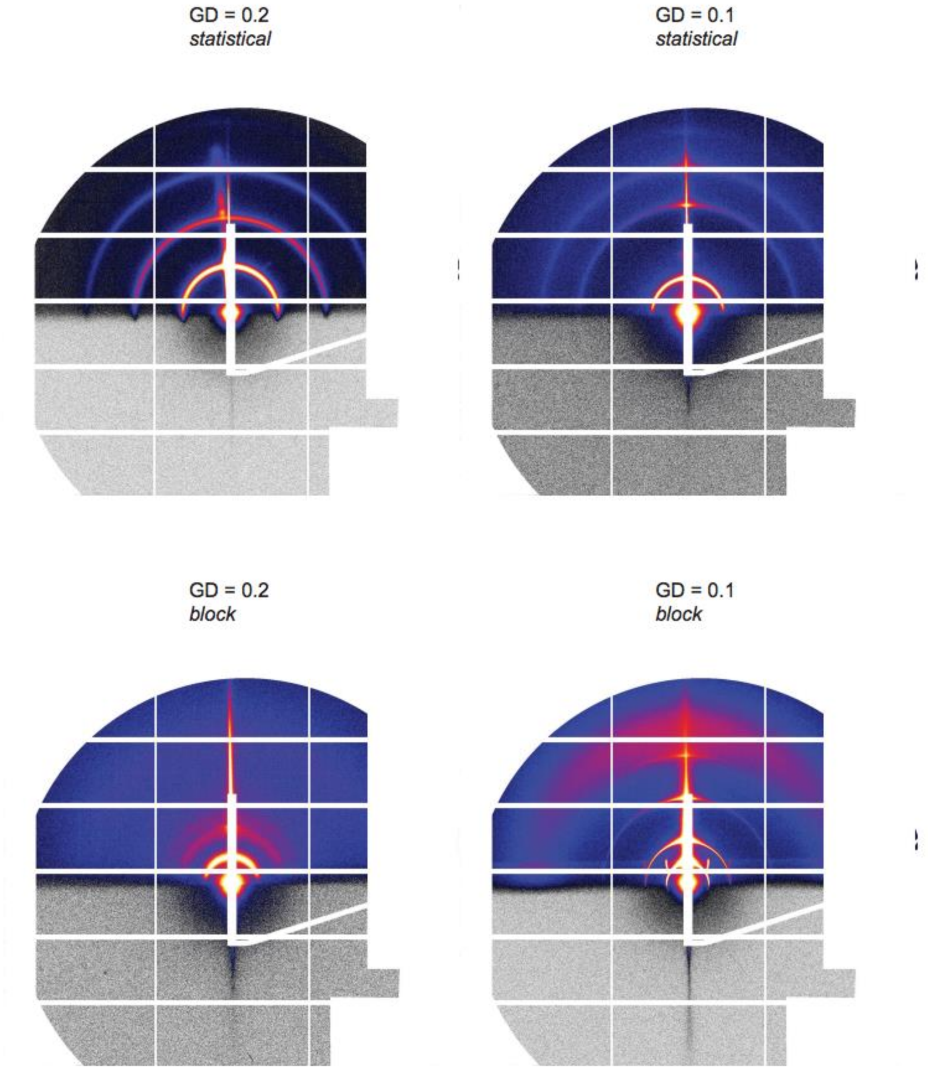

Figure S59: Raw 2D GISAXS – XX-nHexDec-50-XX.

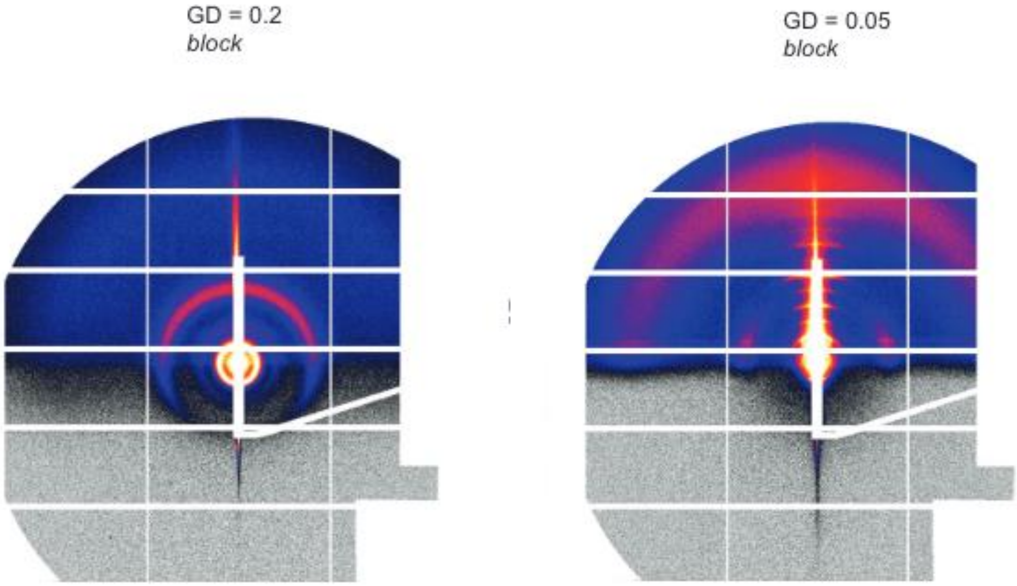

z

Figure S60: Raw 2D GISAXS – *block*-nHexDec-150-XX.

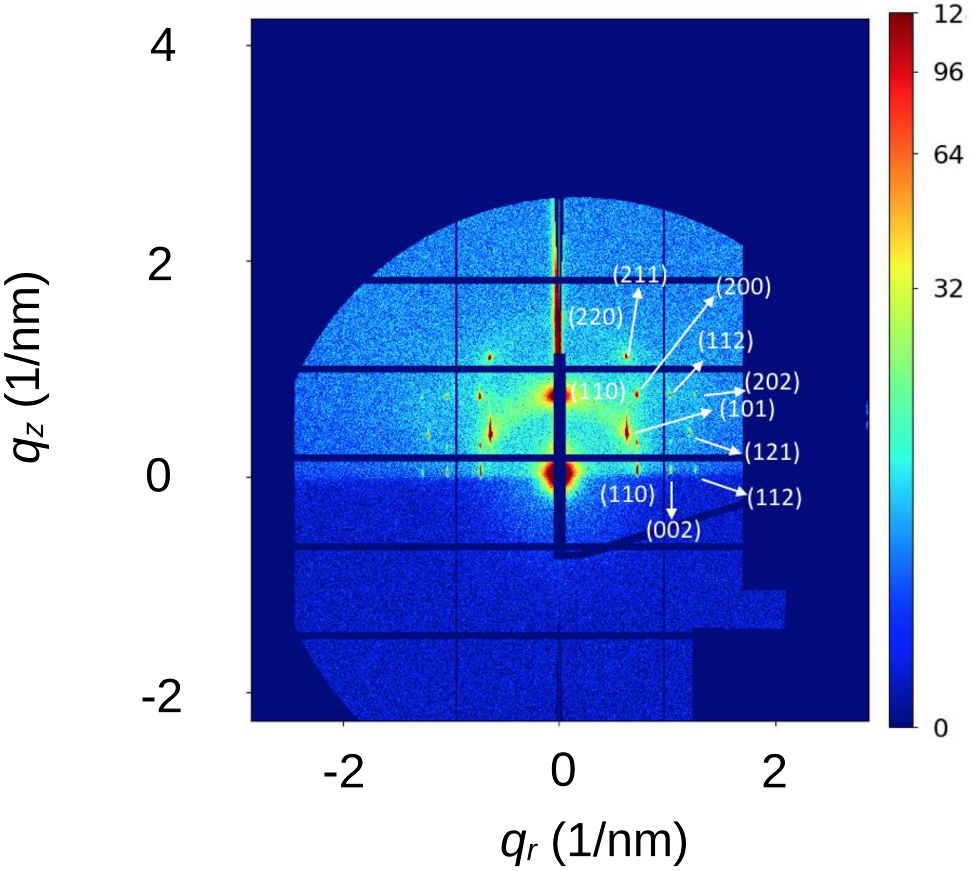

Figure S61: Indexed 2D GISAXS – *stat*-cHex-50-0.6.

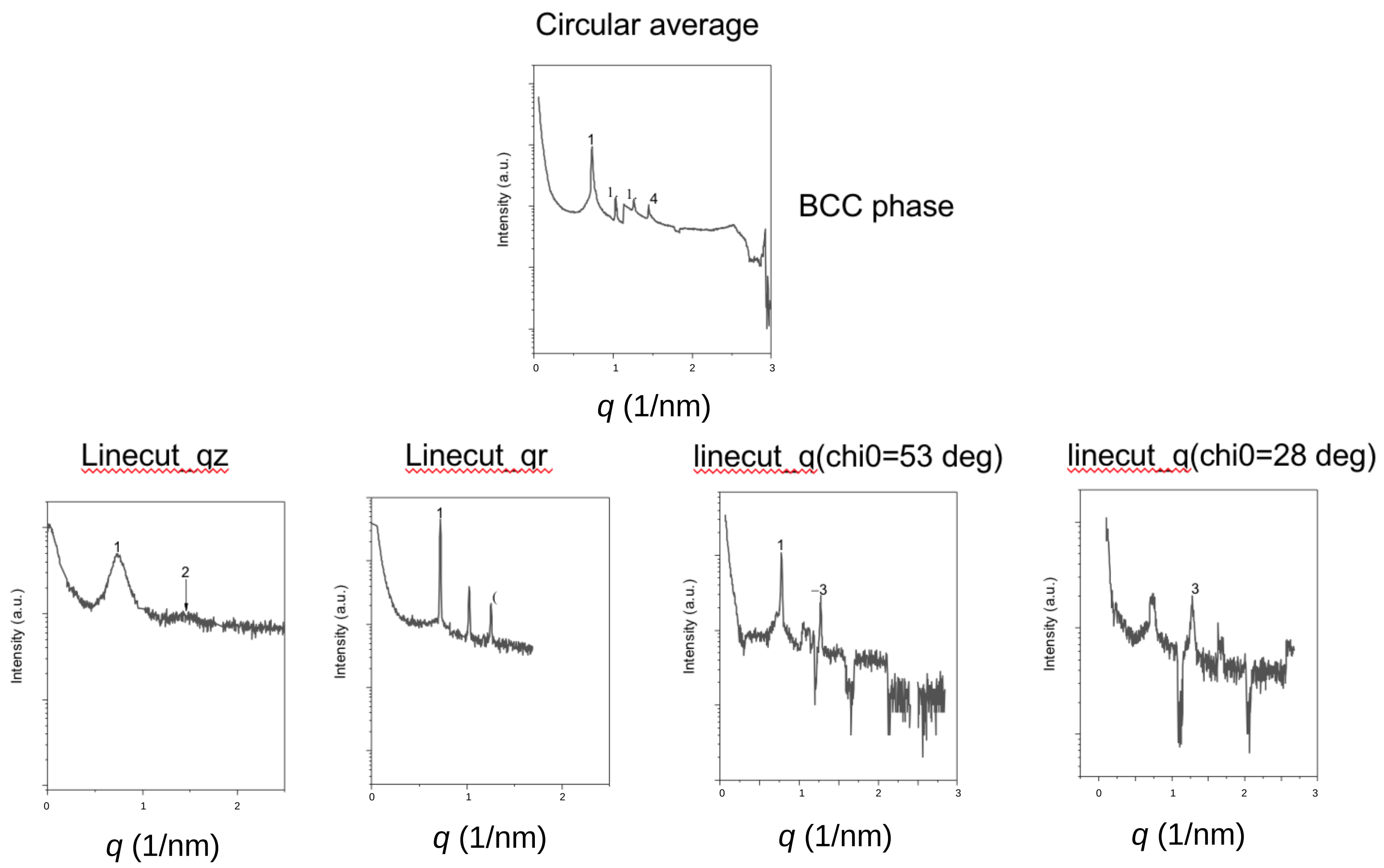

Figure S62: Indexed 1D curves from GISAXS – *stat*-cHex-50-0.6.

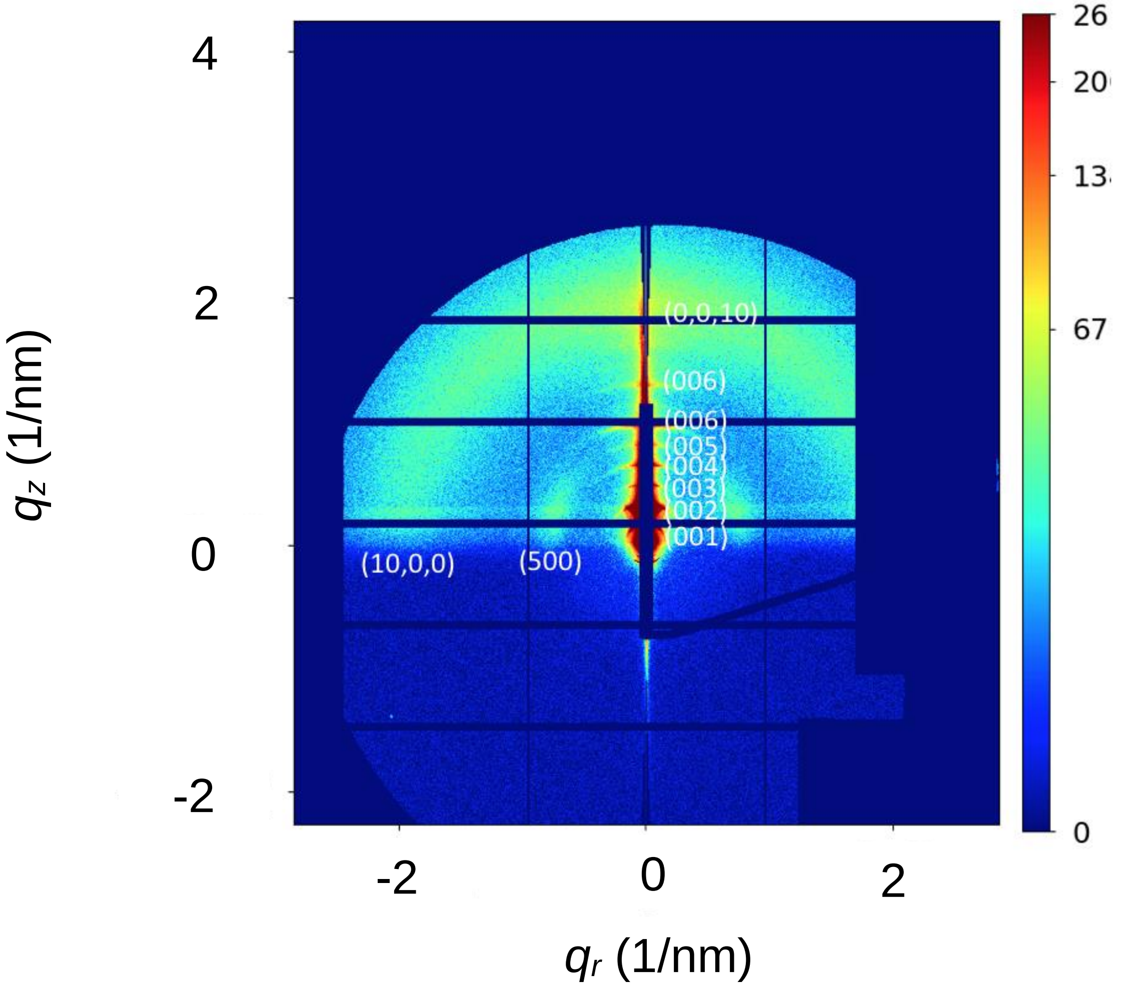

Figure S63: Indexed 2D GISAXS – *block*-nHexDec-150-0.05

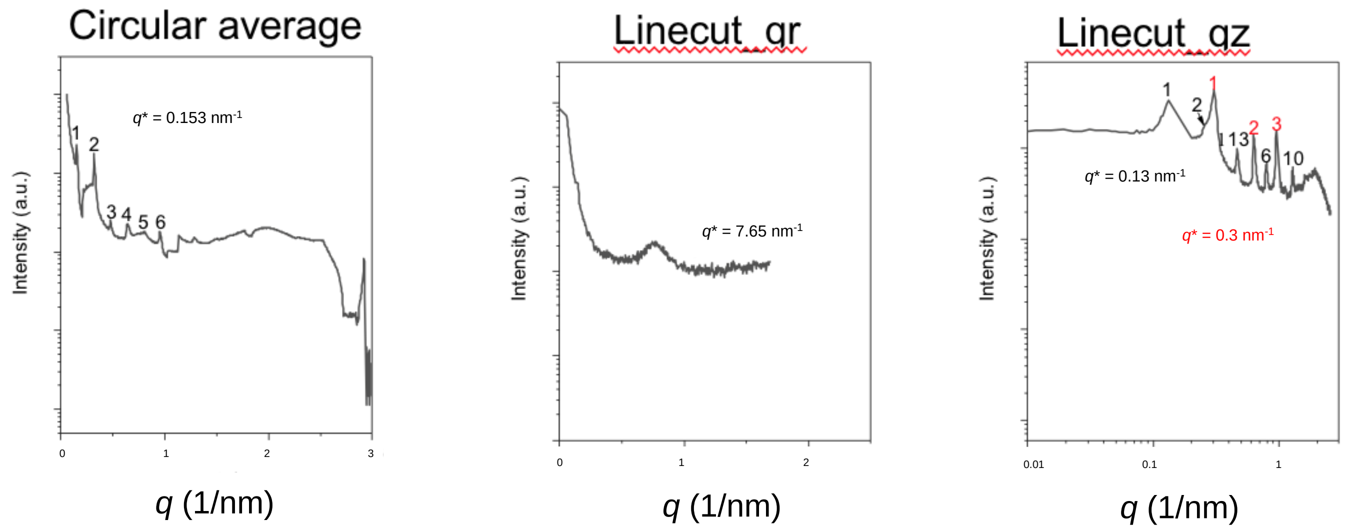

Figure S64: Indexed 1D curves from GISAXS – *block*-nHexDec-150-0.05.

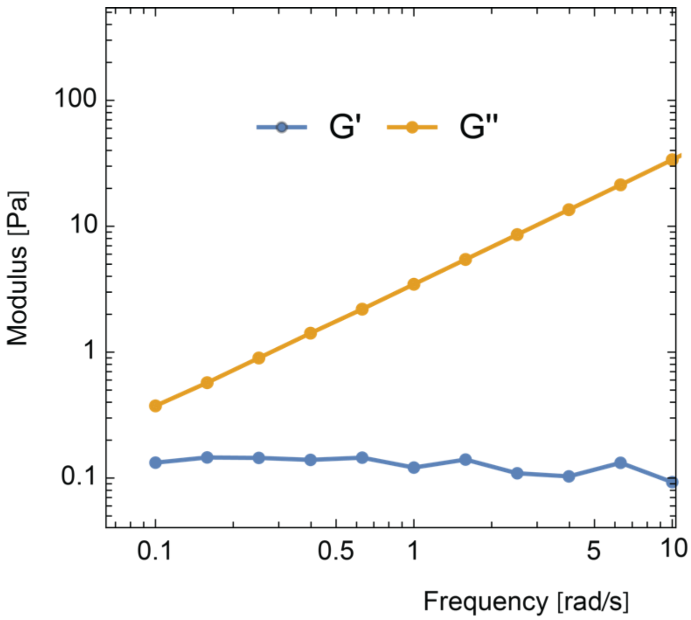

Figure S65: Rheological characterization (constant amplitude frequency sweep) of DIS sample.

Y-axis in Pa, X-axis in Hz. Brief plateau storage modulus ~ 0.1 Pa.

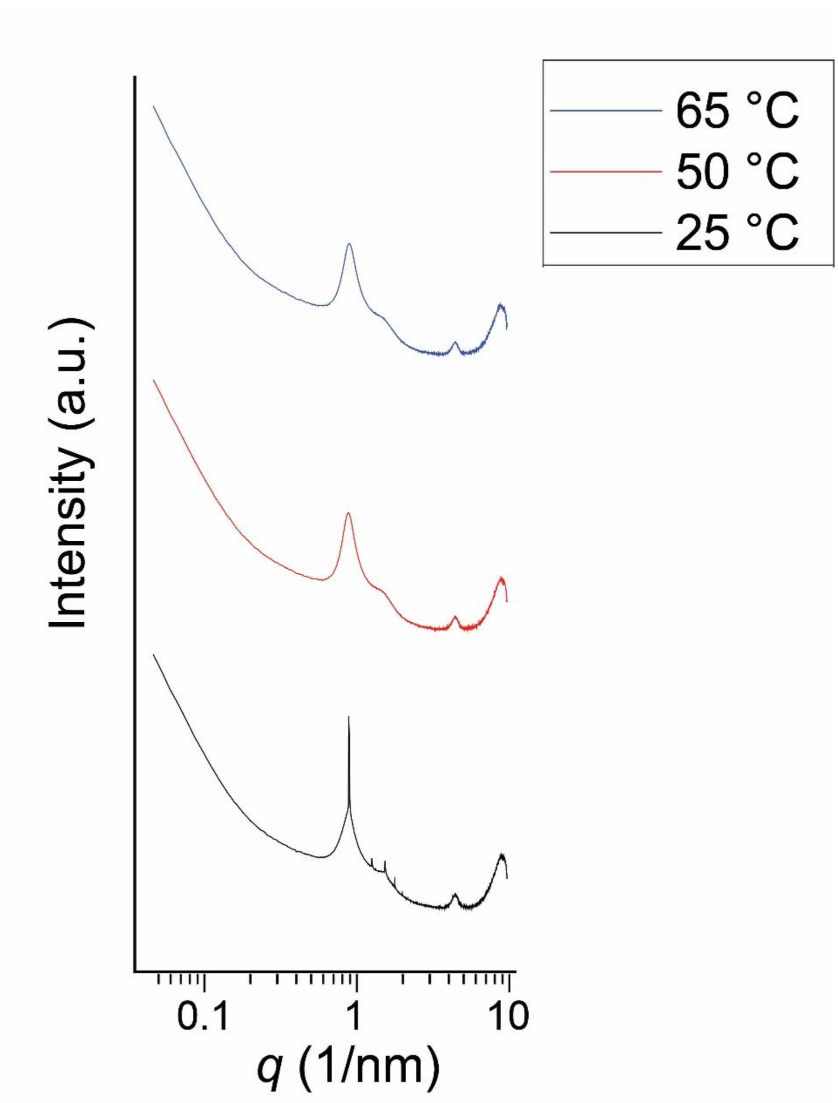

Figure S66: Variable temperature SAXS – *stat*-cHex-50-0.7.

25 °C < T_{ODT} < 50 °C.

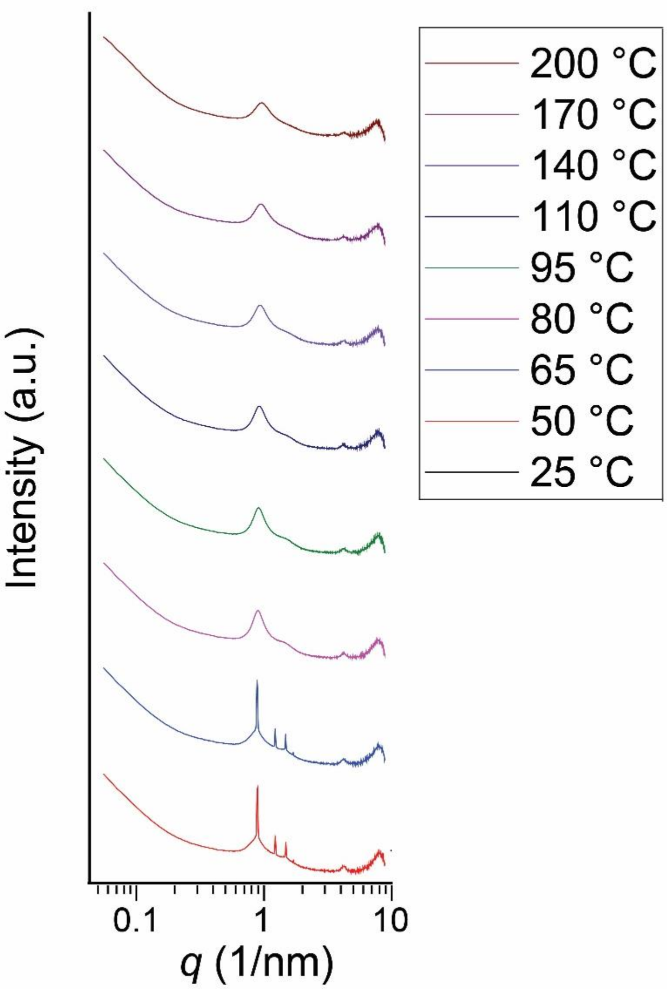

Figure S67: Variable-temperature SAXS – *stat*-cHex-50-0.6.

65 °C < T_{ODT} < 80 °C.

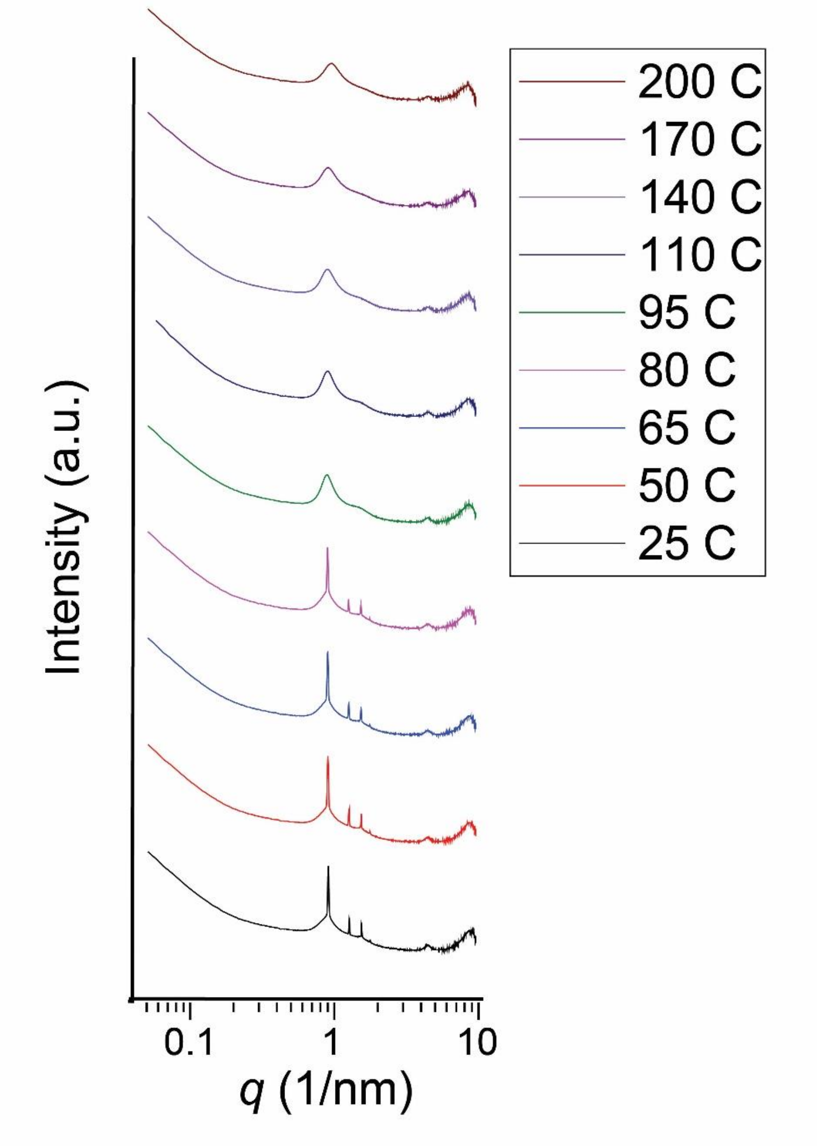

Figure S68: Variable-temperature SAXS – *stat*-cHex-150-0.6.

80 °C < T_{ODT} < 95 °C.

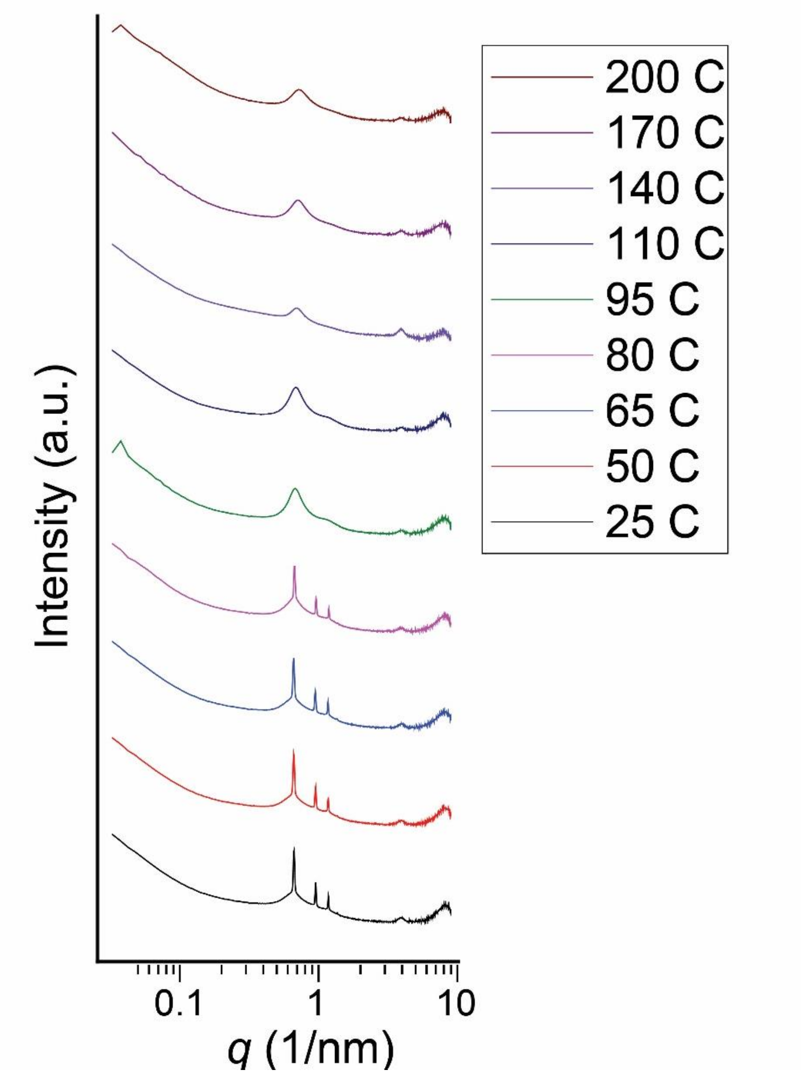

Figure S69: Variable temperature SAXS – *stat*-cHex-50-0.2.

80 °C < T_{ODT} < 95 °C.

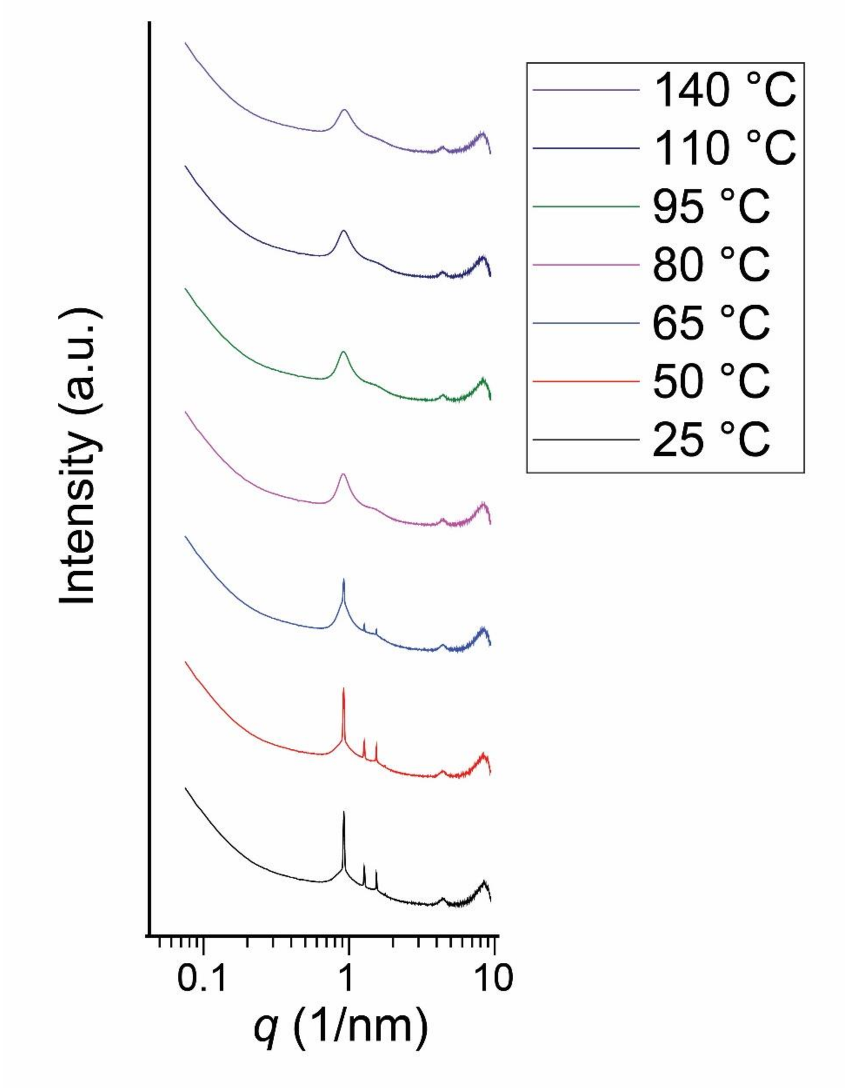

Figure S70: Variable-temperature SAXS – *stat*-nHex-150-0.6.

65 °C < T_{ODT} < 80 °C.

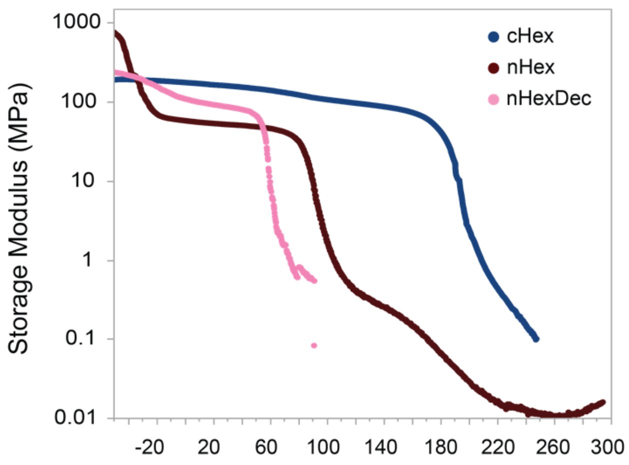

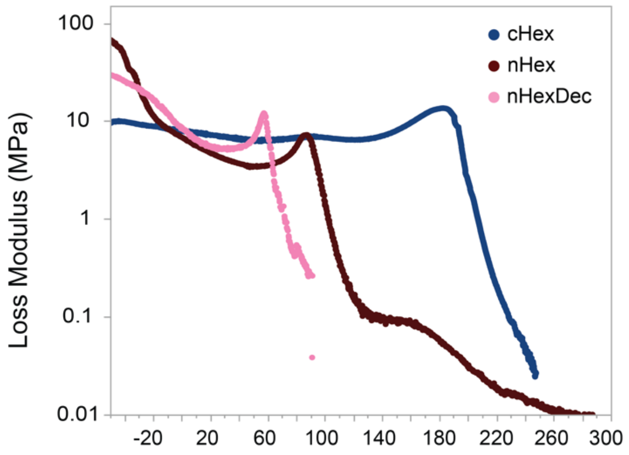

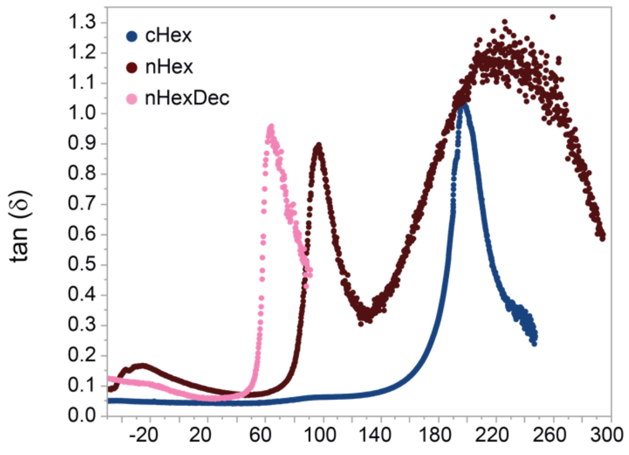

Figure S71: Representative E', E'' and tan(d) traces plotted vs temperature of statistical copolymers obtained by DMA.

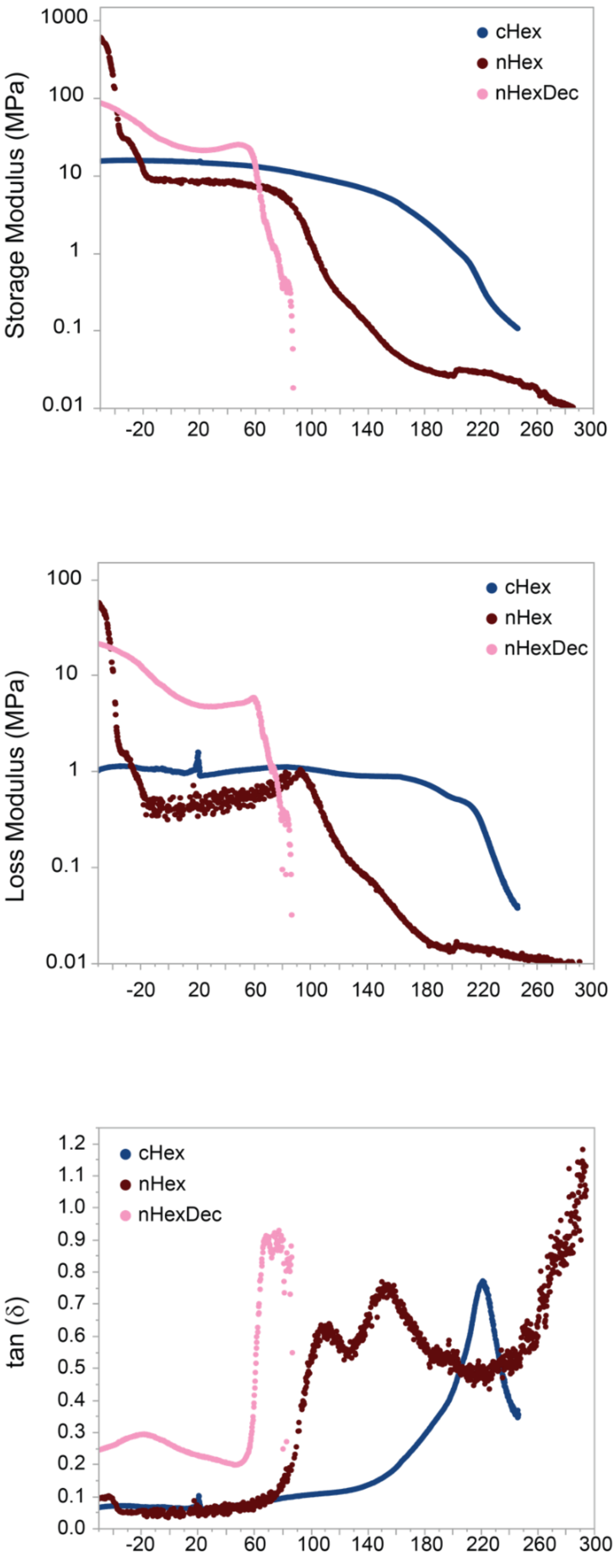

Figure S72: Representative E', E'' and tan(d) traces plotted vs temperature, of block copolymers obtained by DMA.

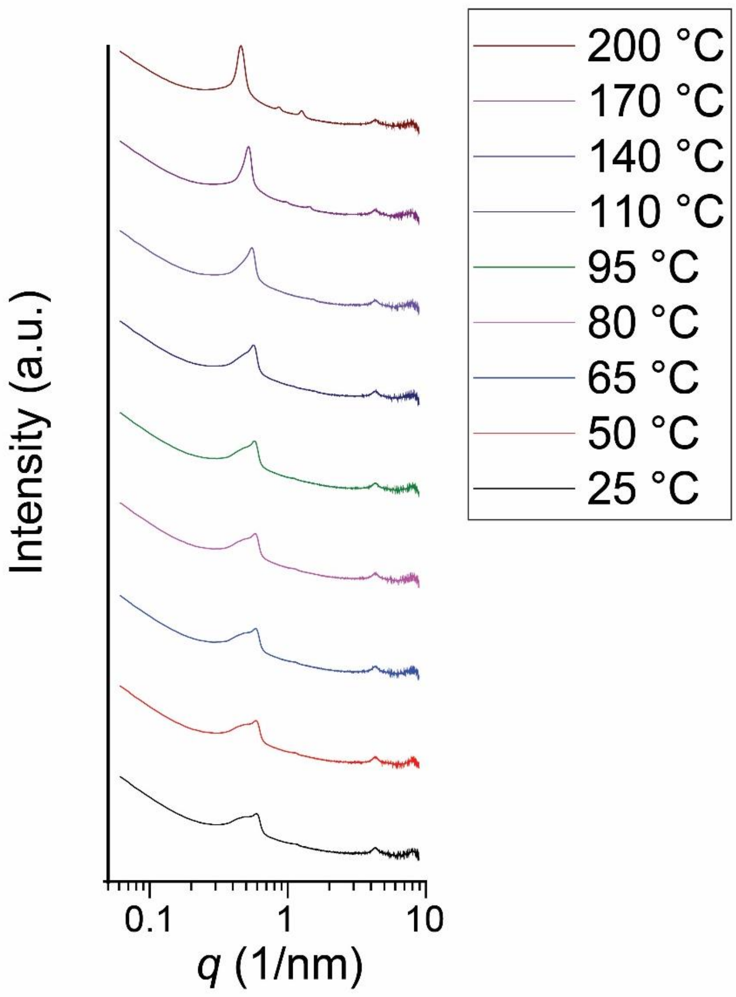

Figure S73: Variable-temperature SAXS – *stat*-cHex-150-0.05.

T_{ODT} > 200 °C. Domain spacing increases from 16.3 nm at 25 °C to 17.6 nm at 200 °C.

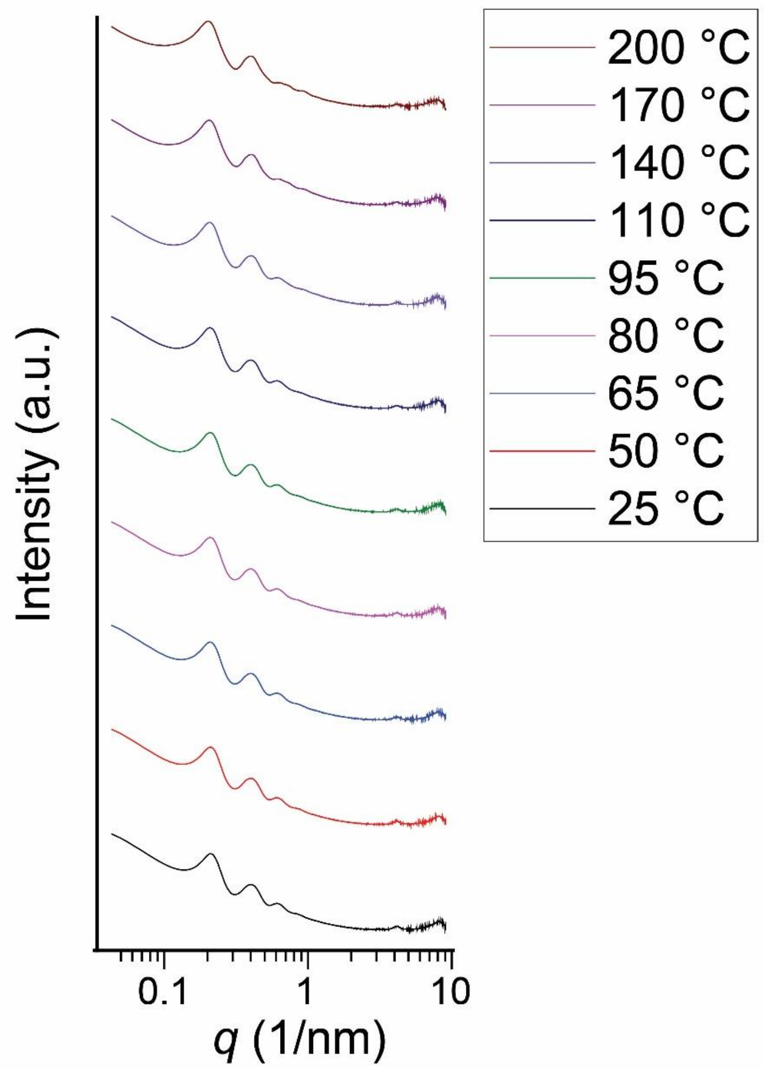

Figure S74: Variable-temperature SAXS – *block*-cHex-150-0.05.

T_{ODT} > 200 °C. Domain spacing increases from 31.5 nm at 25 °C to 33.0 nm at 200 °C.

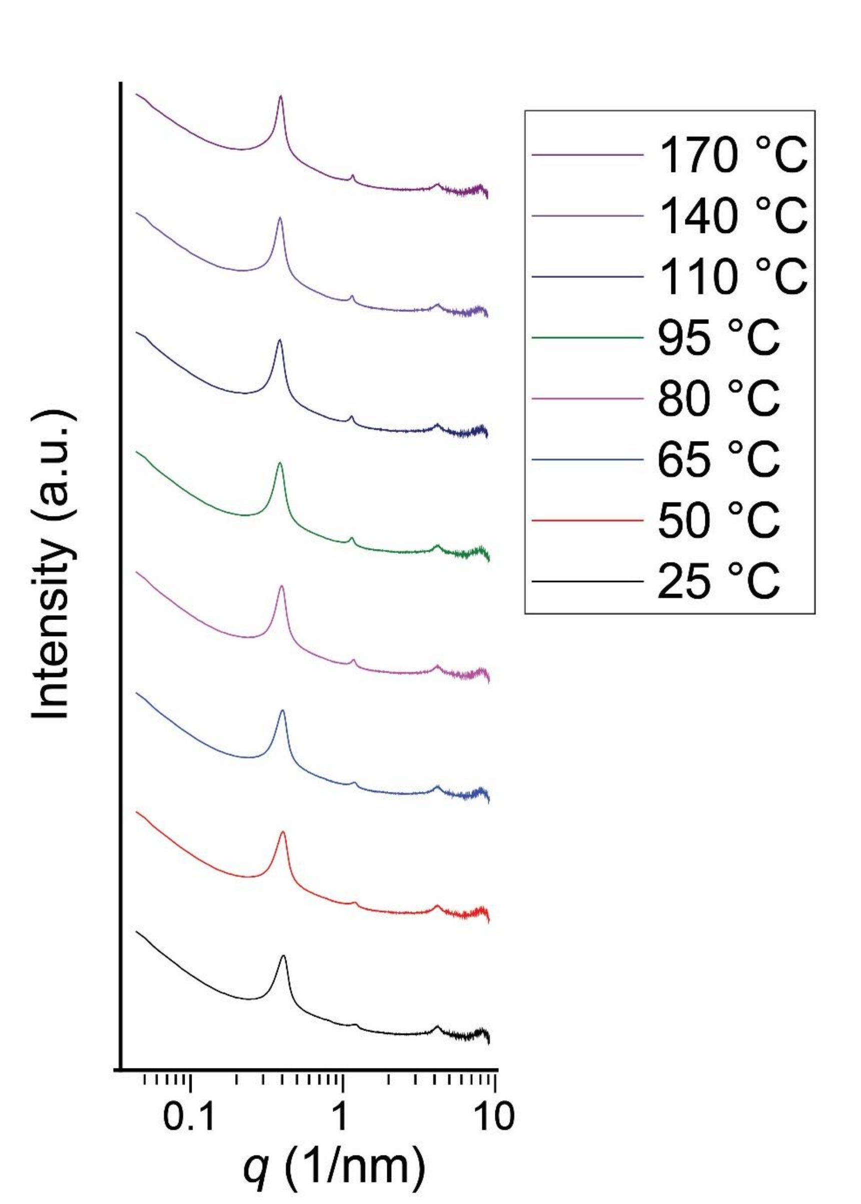

Figure S75: Variable-temperature SAXS – *stat*-nHex-150-0.05.

T_{ODT} > 170 °C. Domain spacing increases from 16.6 nm at 25 °C to 17.1 nm at 170 °C.

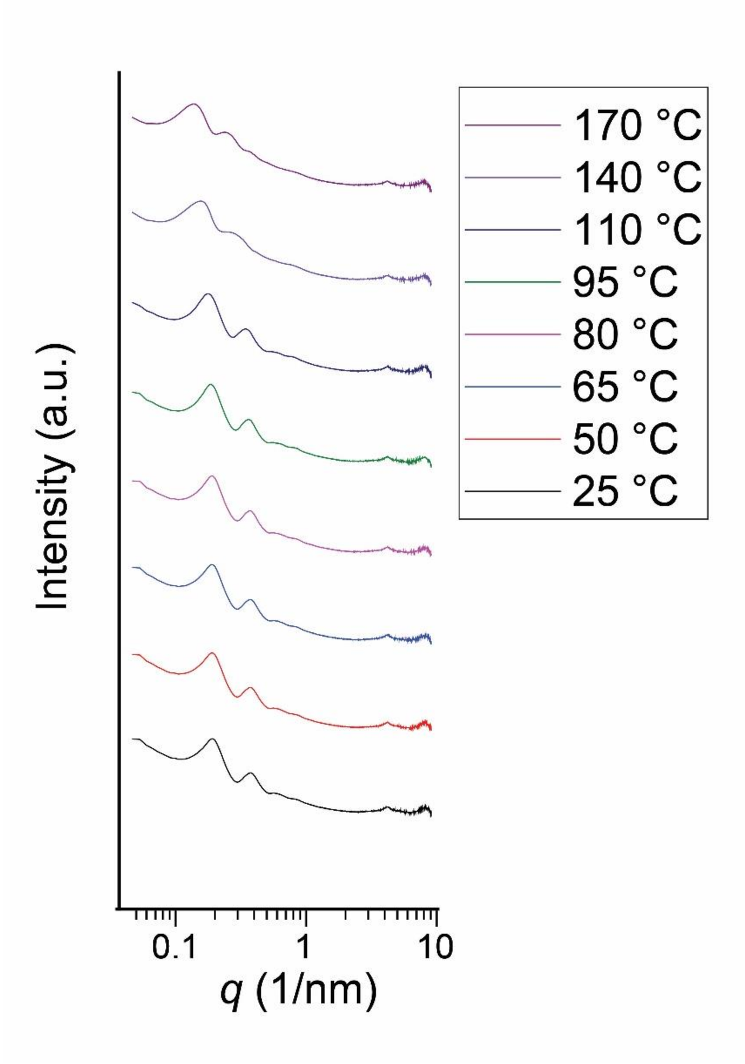

Figure S76: Variable-temperature SAXS – *block*-nHex-150-0.05.

T_{ODT} > 170 °C. Domain spacing increases from 37.1 nm at 25 °C to 52.1 nm at 170 °C.

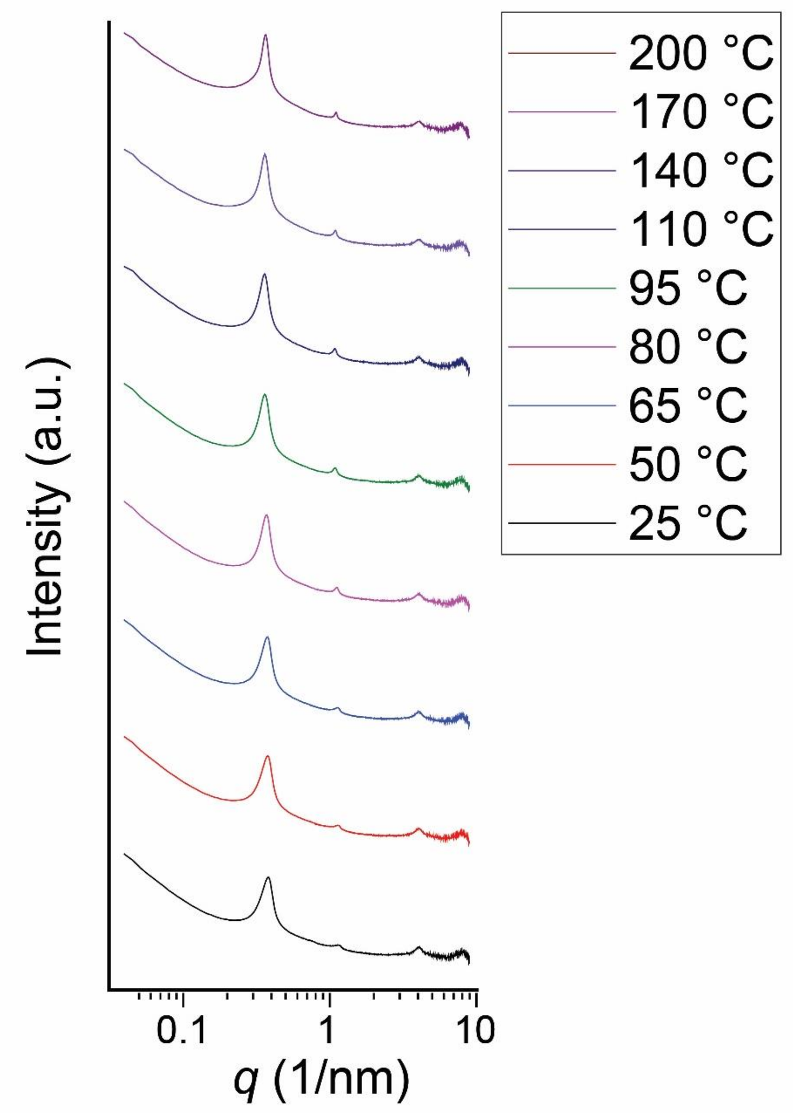

Figure S77: Variable-temperature SAXS – *stat*-nHexDec-150-0.05.

T_{ODT} > 170 °C.

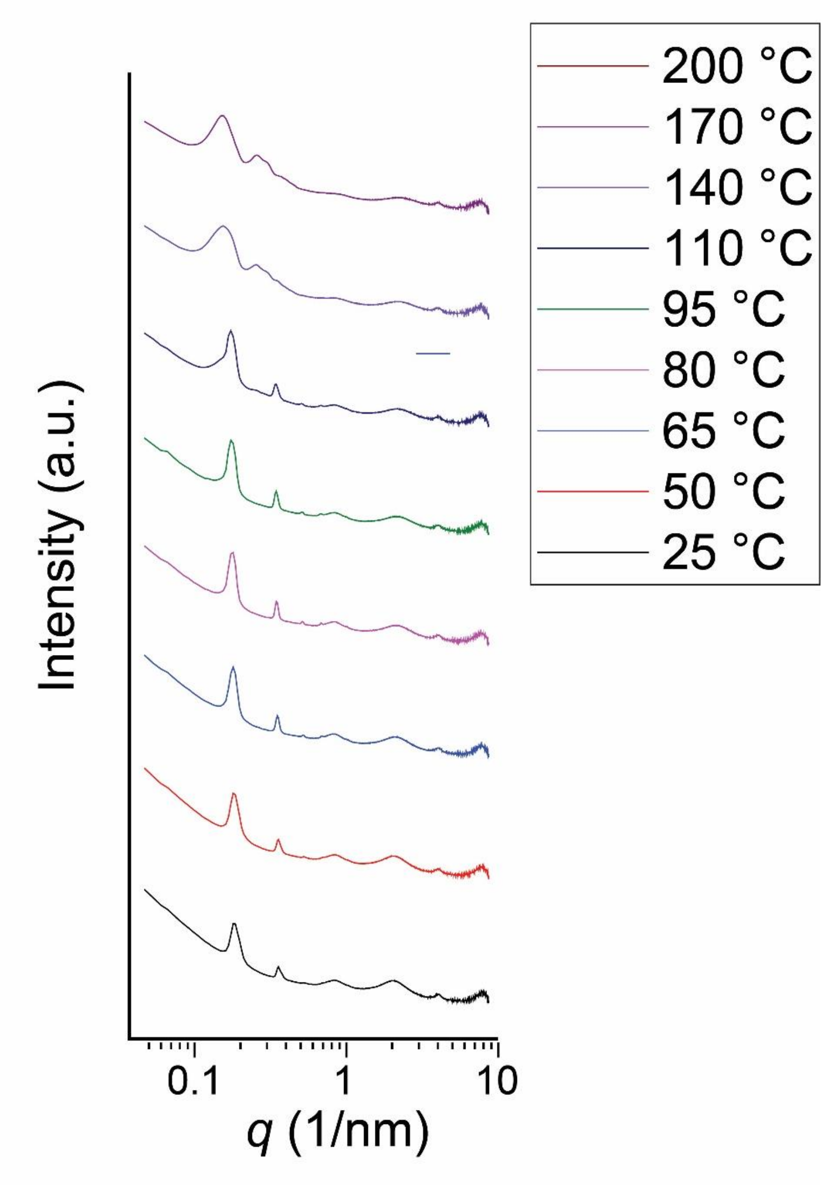

Figure S78: Variable-temperature SAXS – *block*-nHexDec-150-0.05.

T_{ODT} > 170 °C. Domain spacing increases from 38.6 nm at 25 °C to 46.3 nm at 170 °C.

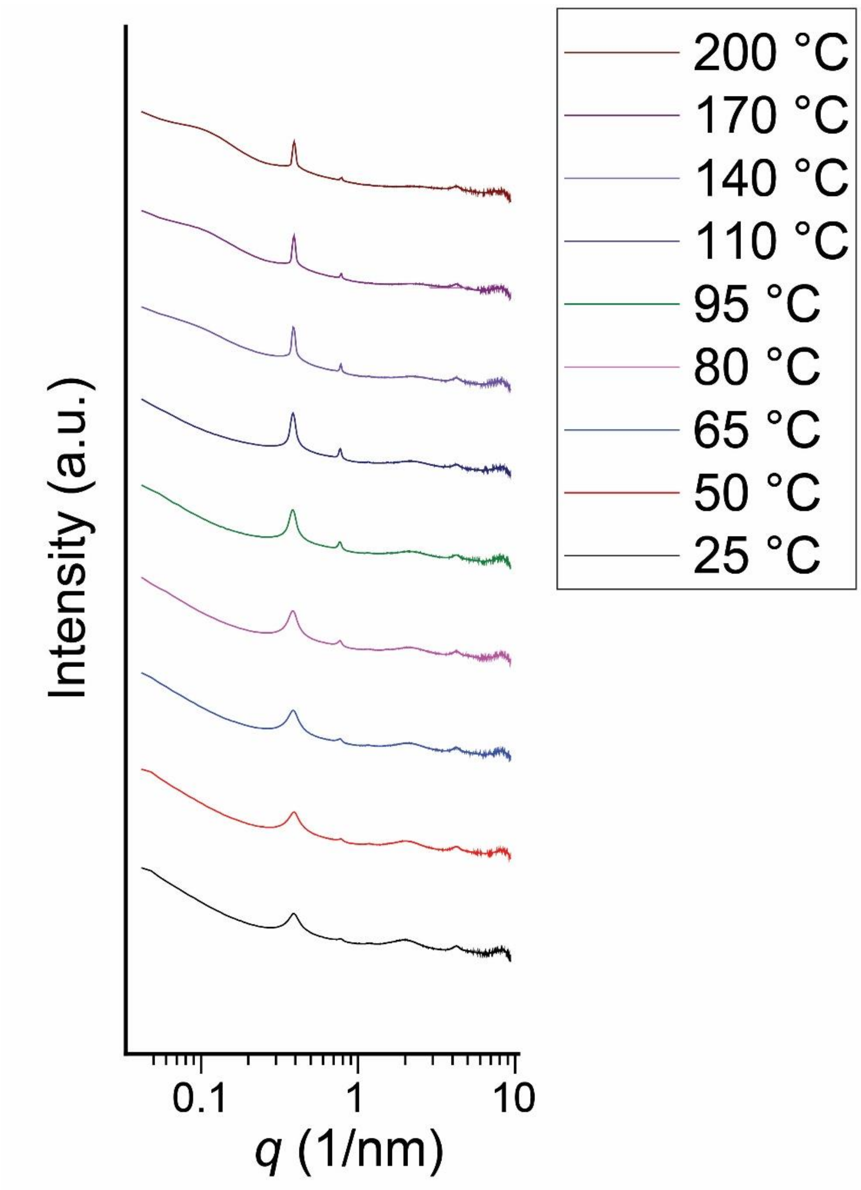

Figure S79: Variable-temperature SAXS – *stat*-nHexDec-50-0.05.

T_{ODT} > 200 °C. Broad feature corresponding to 72.2 nm appears at high T. Other features disappear or sharpen.

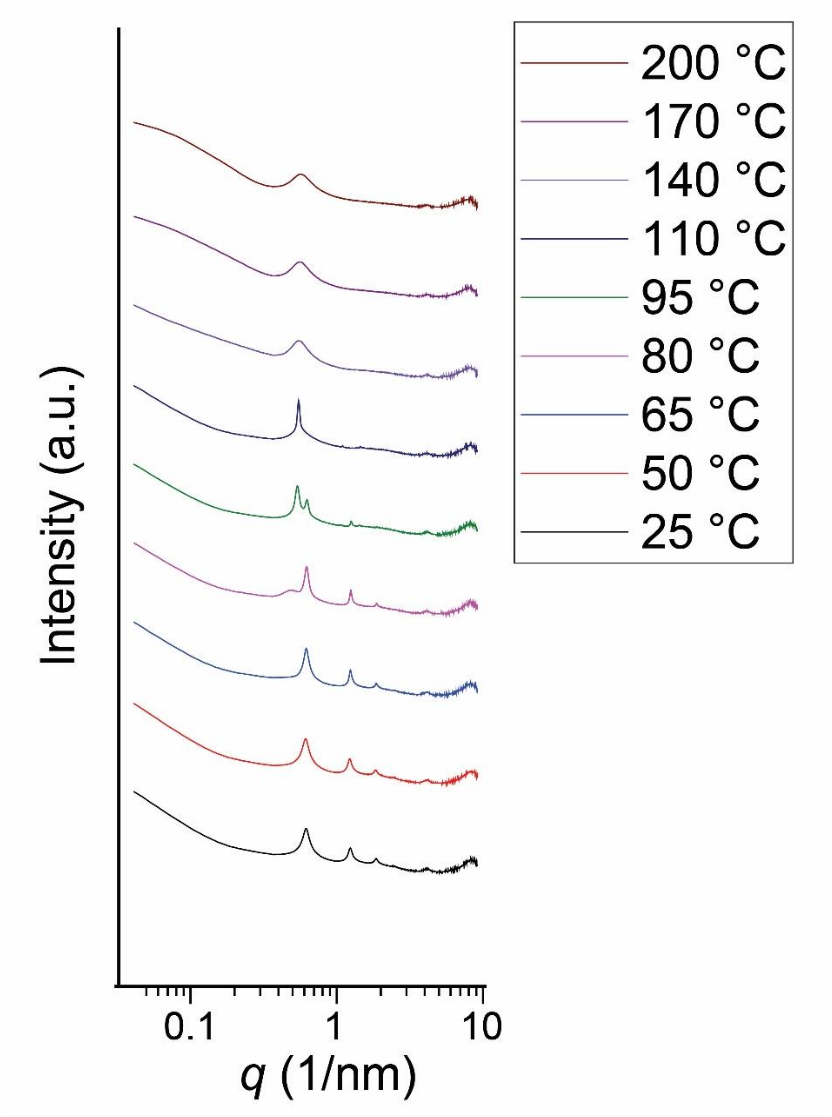

Figure S80: Variable-temperature SAXS – *stat*-nHexDec-50-0.2.

95 °C < T_{OOT} < 110 °C from lamellae with domain spacing 10.4 nm to cylinders with domain spacing 11.7 nm.

110 °C < T_{ODT} < 140 °C

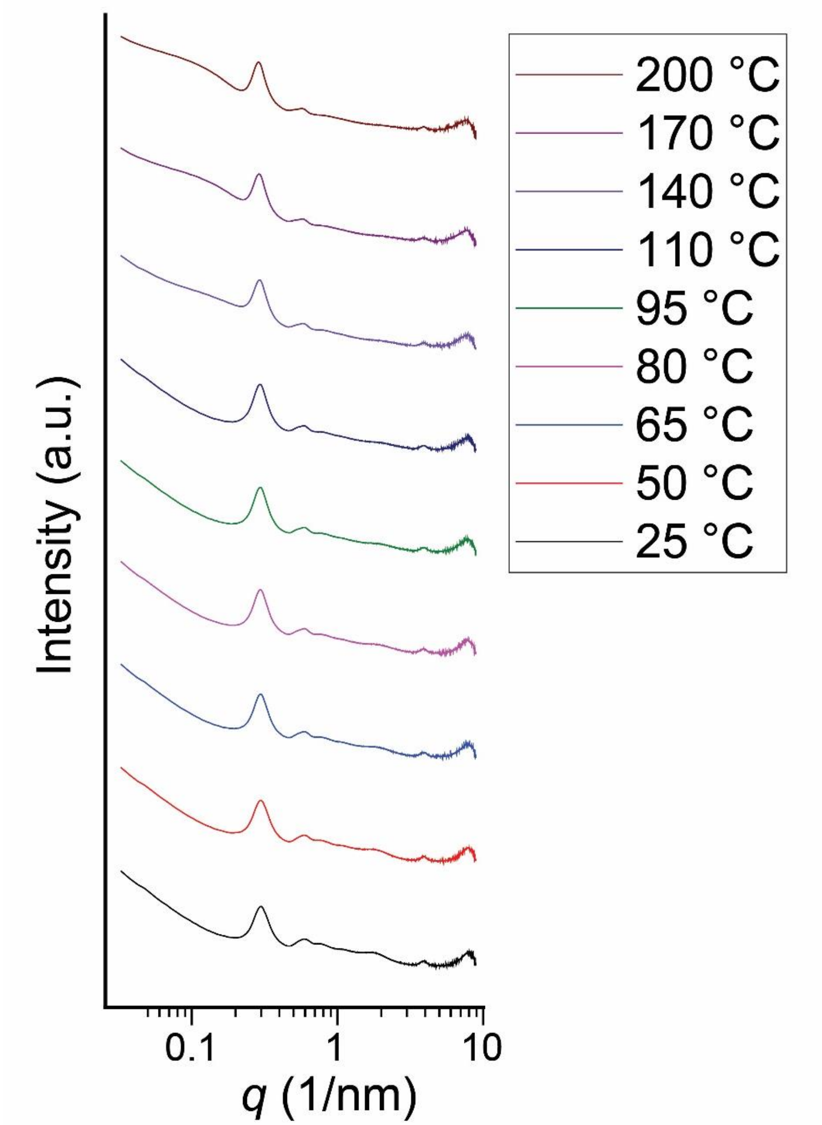

Figure S81: Variable-temperature SAXS – *block*-nHexDec-50-0.2.

T_{OOT} < 200 °C from CYL with domain spacing 18.5 nm to LAM with domain spacing 19.2 nm and a broad feature at 63 nm.

T_{ODT} > 200 °C

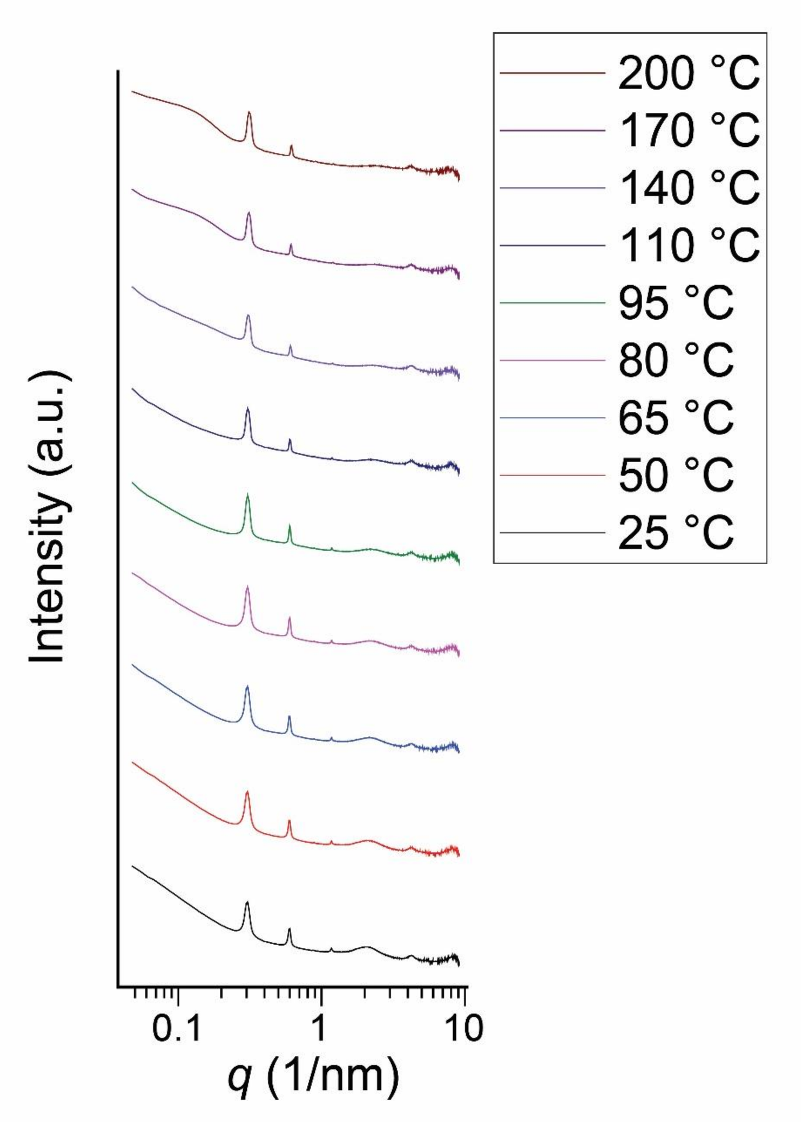

Figure S82: Variable-temperature SAXS- *block*-nHexDec-50-0.05.

T_{ODT} >170 °C. Domain spacing decreases from 23.4 nm at 25 °C to 22.7 nm at 170 °C.

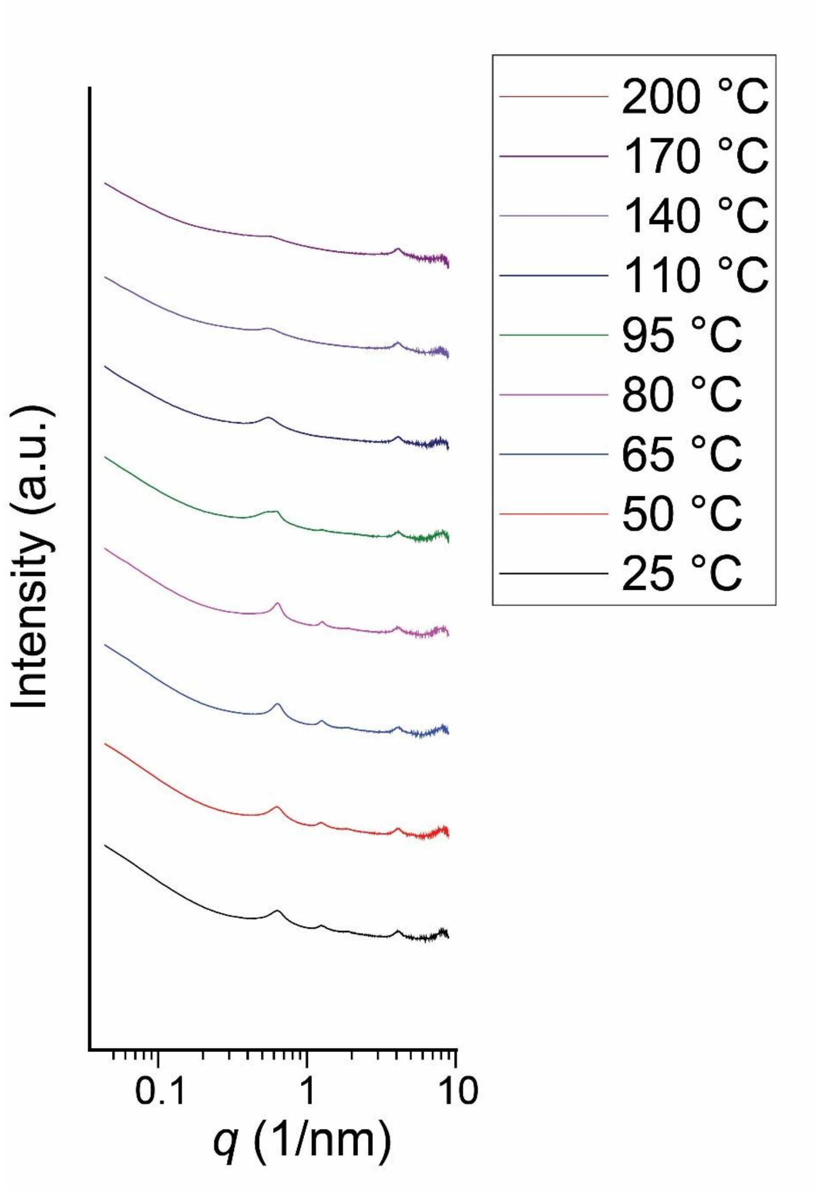

Figure S83: Variable-temperature SAXS – *stat*-nHexDec-150-0.2.

80 °C < T_{ODT} < 95 °C.

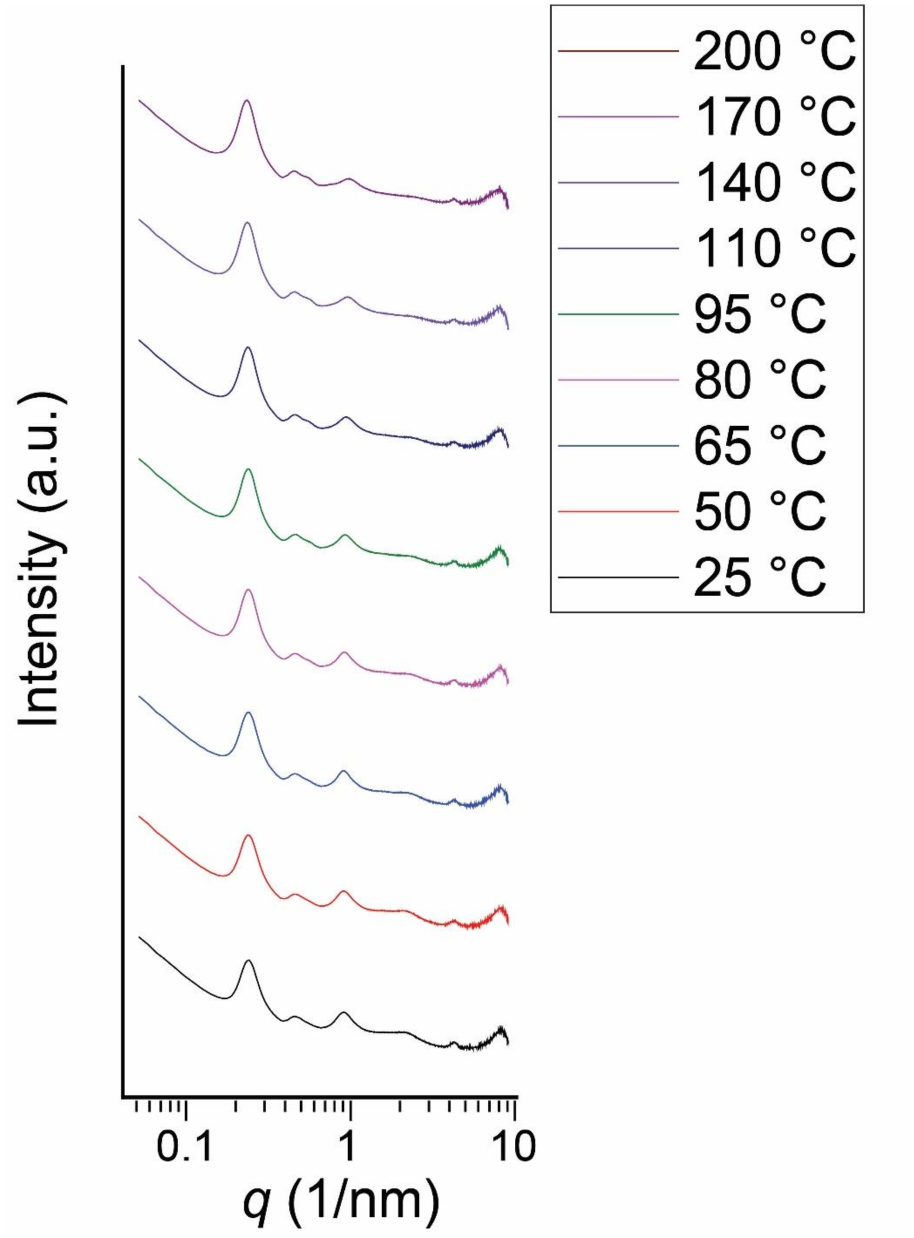

Figure S84: Variable-temperature SAXS – *block*-nHexDec-150-0.2.

T_{ODT} > 170 °C. Domain spacing increases from 31.3 nm at 25 °C to 32.1 nm at 170 °C.

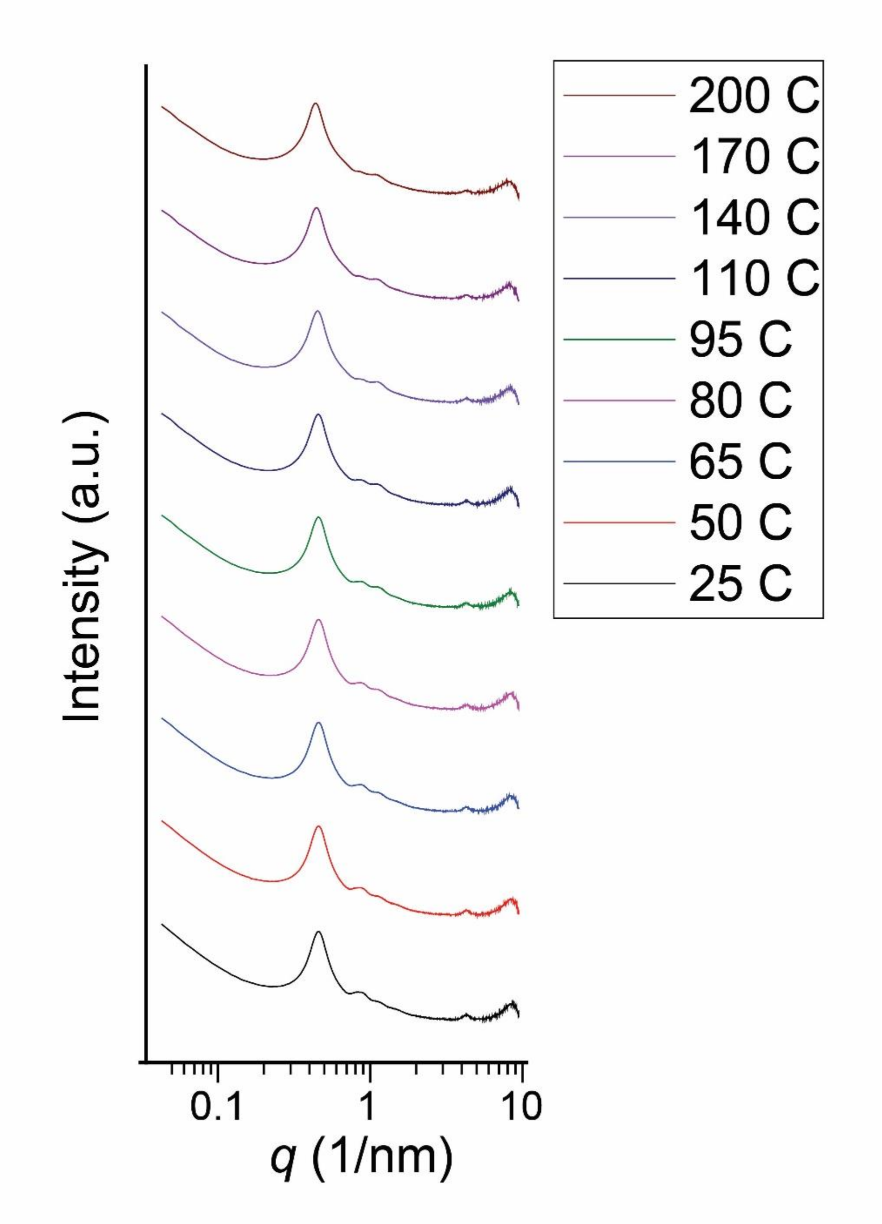

Figure S85: Variable temperature SAXS – *block*-cHex-50-0.2.

T_{ODT} > 200 °C.

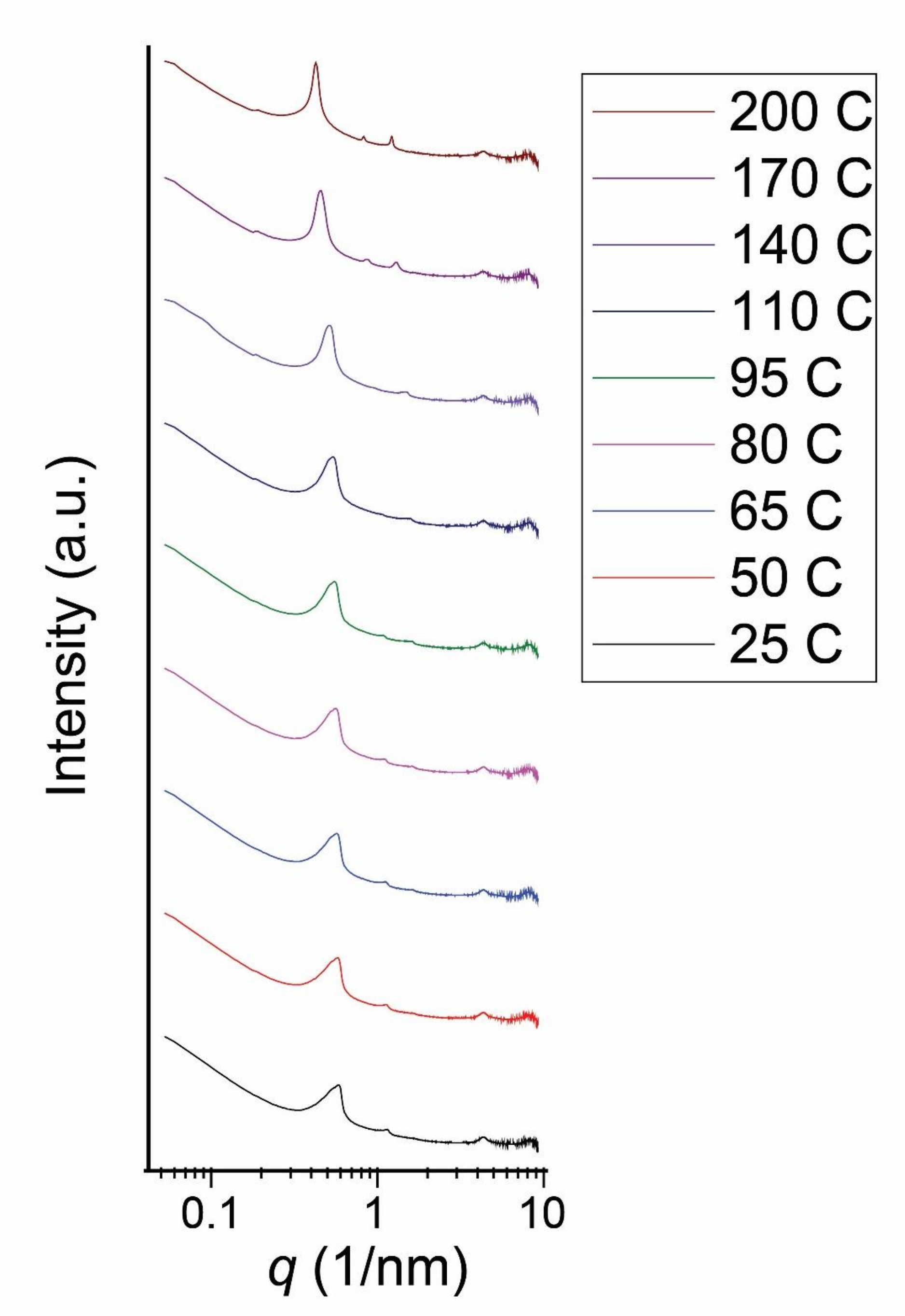

Figure S86: Variable-temperature SAXS – *stat*-cHex-50-0.05.

T_{ODT} > 200 °C. Domain spacing increases from 13.0 nm at 25 °C to 17.6 nm at 200 °C.

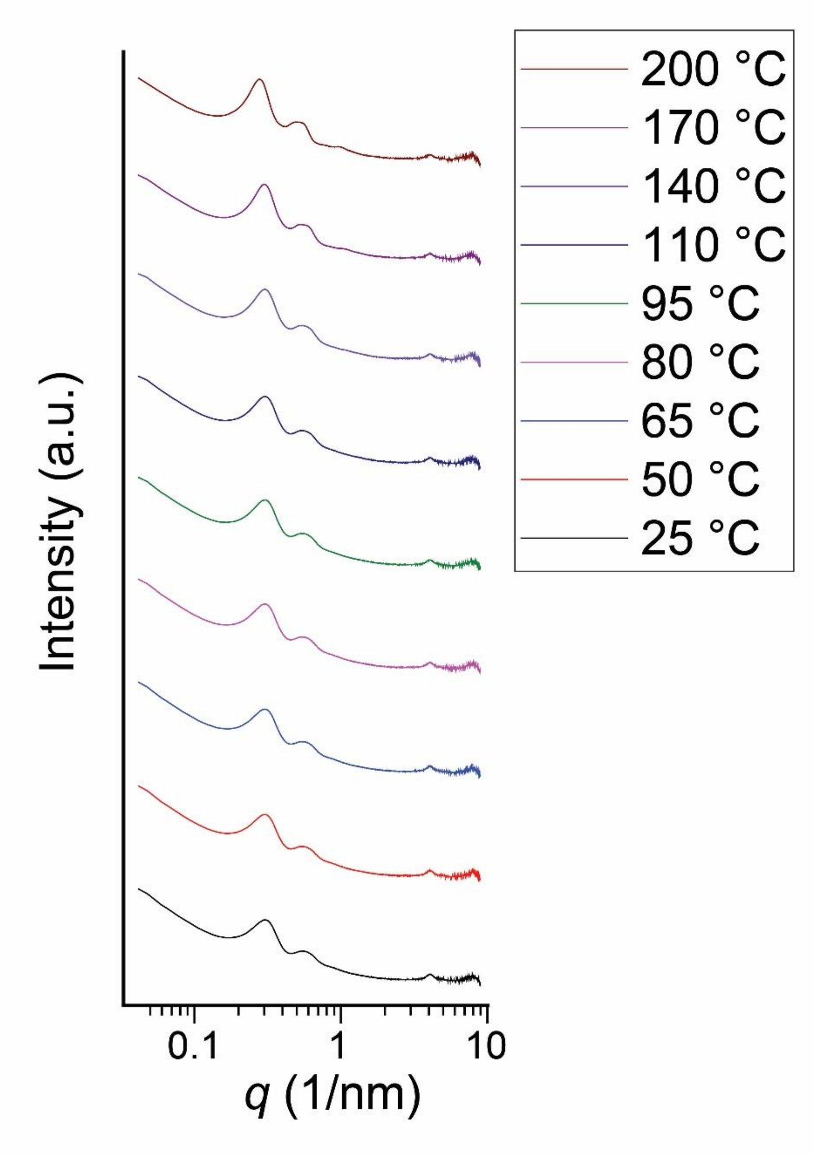

Figure S87: Variable-temperature SAXS – *block*-cHex-50-0.05.

T_{ODT} > 200 °C. Domain spacing increases from 21.0 to 23.0 nm from 25 °C to 200 °C.

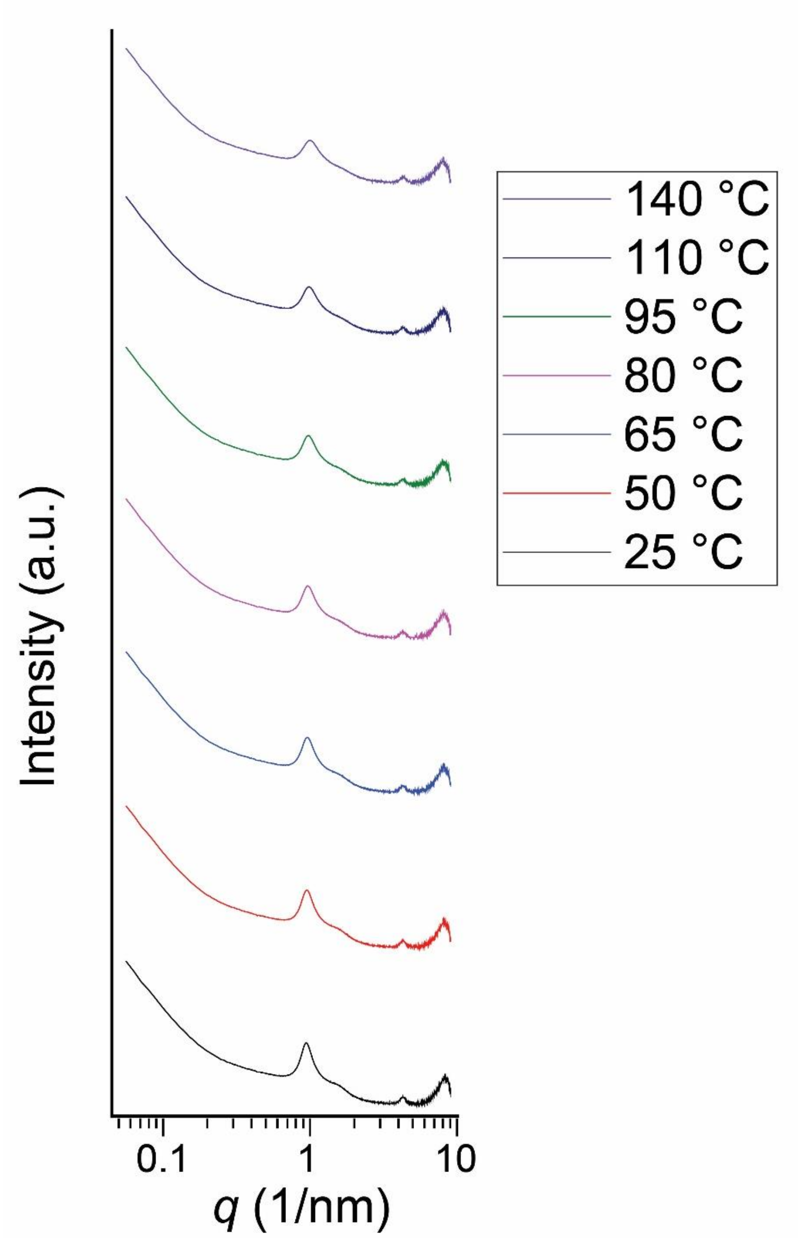

Figure S88: Variable-temperature SAXS – *stat*-nHex-50-0.7.

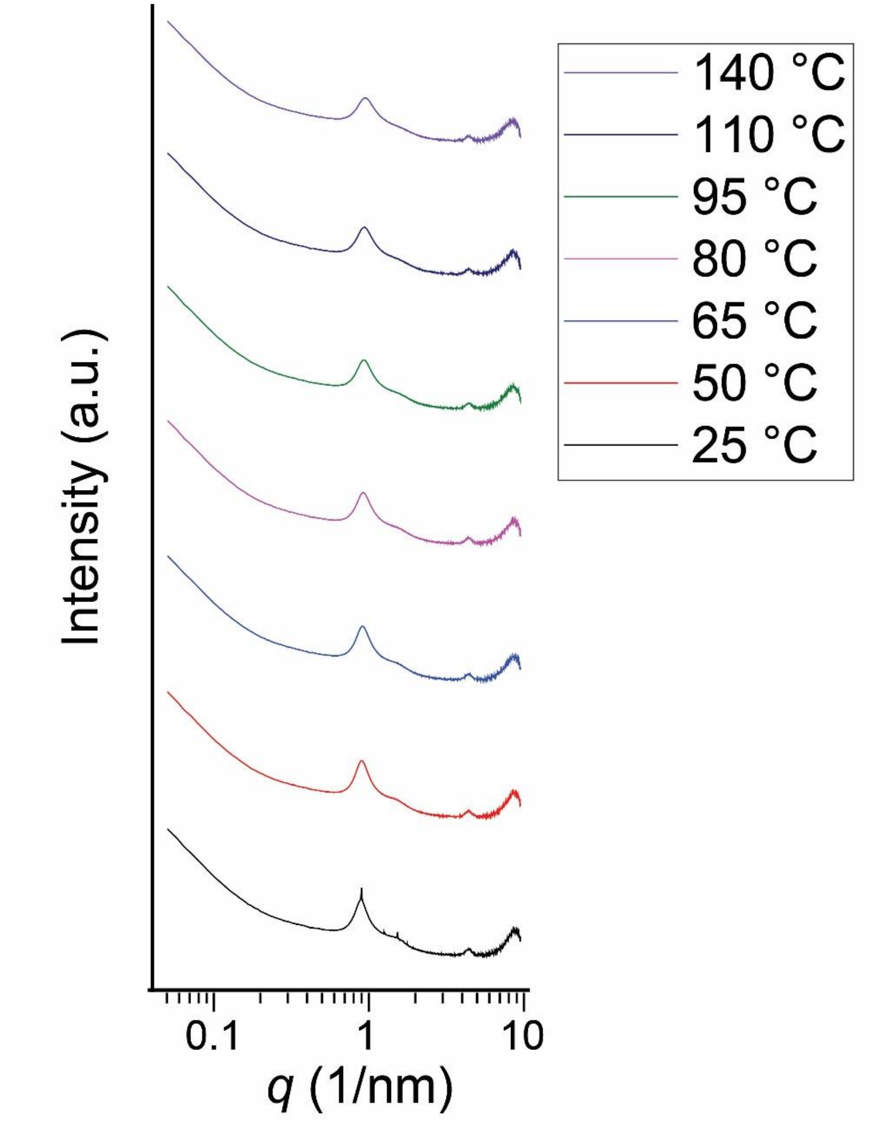

Figure S89: Variable-temperature SAXS – *stat*-nHex-50-0.6.

25 °C < T_{ODT} < 50 °C

SM	N_{bb}	GD	SEQUENCE	Morphology	T_{ODT} (°C)	Notes
cHex	50	0.7	*stat*	SPH	50-65	SPH--> DIS
cHex	50	0.6	*stat*	SPH	65-80	SPH--> DIS
cHex	150	0.6	*stat*	SPH	80-95	SPH--> DIS
cHex	50	0.2	*stat*	SPH	80-95	SPH--> DIS
cHex	50	0.2	*block*	CYL	>200	decreasing order w T
cHex	50	0.05	*stat*	LAM	>200	13 to 17.6
cHex	50	0.05	*block*	LAM	>200	21 to 23
cHex	150	0.05	*stat*	LAM	>200	16.3 to 17.6
cHex	150	0.05	*block*	LAM	>200	31.5 to 33
nHex	50	0.6	*stat*	SPH	25-50	SPH--> DIS
nHex	150	0.6	*stat*	SPH	65-80	SPH--> DIS
nHex	150	0.05	*stat*	LAM	>170	16.6 to 17.1
nHex	150	0.05	*block*	LAM	>170	37.1 to 52.1
nHexDec	50	0.2	*stat*	LAM	95-110, 110-140	LAM-->C-YL-> DIS
nHexDec	50	0.2	*block*	CYL	BROAD	CYL -> LAM
nHexDec	50	0.05	*stat*	LAM	>200	72.2 nm feature appears at high T
nHexDec	50	0.05	*block*	LAM	>200	23.4 to 22.7, 63 nm feature at high T
nHexDec	150	0.2	*stat*	LAM	80-95	LAM--> DIS
nHexDec	150	0.2	*block*	LAM	>170	31.3 to 32.1
nHexDec	150	0.05	*stat*	LAM	>170	
nHexDec	150	0.05	*block*	LAM	>170	38.6 to 46.3

Table S6: Microphase separation transition temperatures as measured by VT-SAXS

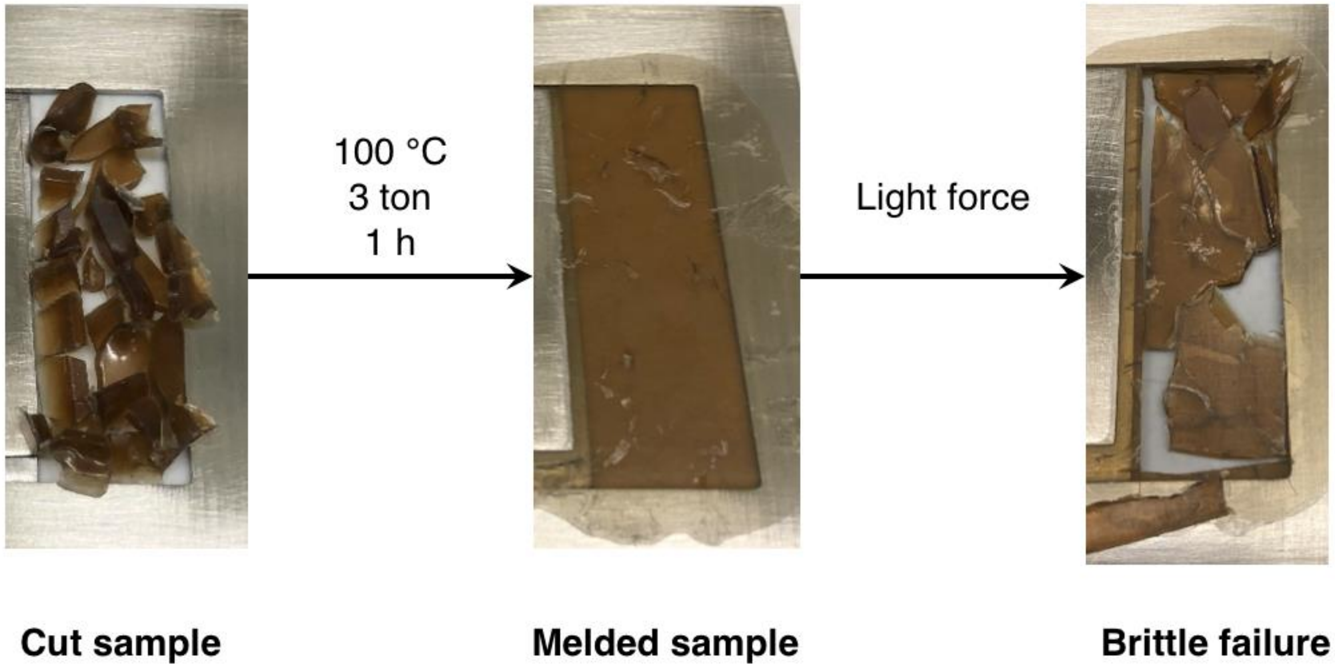

Figure S90: Attempts to thermally reprocess stat-nHexDec-150-0.05 yield macroscopically cohesive materials which are susceptible to brittle failure.

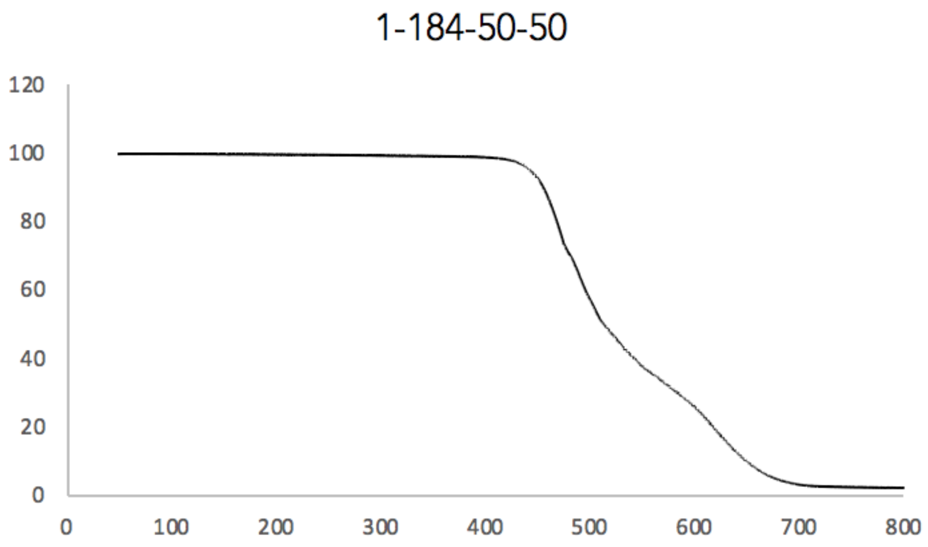

Figure S91: Thermogravimetric Analysis of Select Sample, stat-cHex-50-0.5. % residual mass versus temperature in °C.

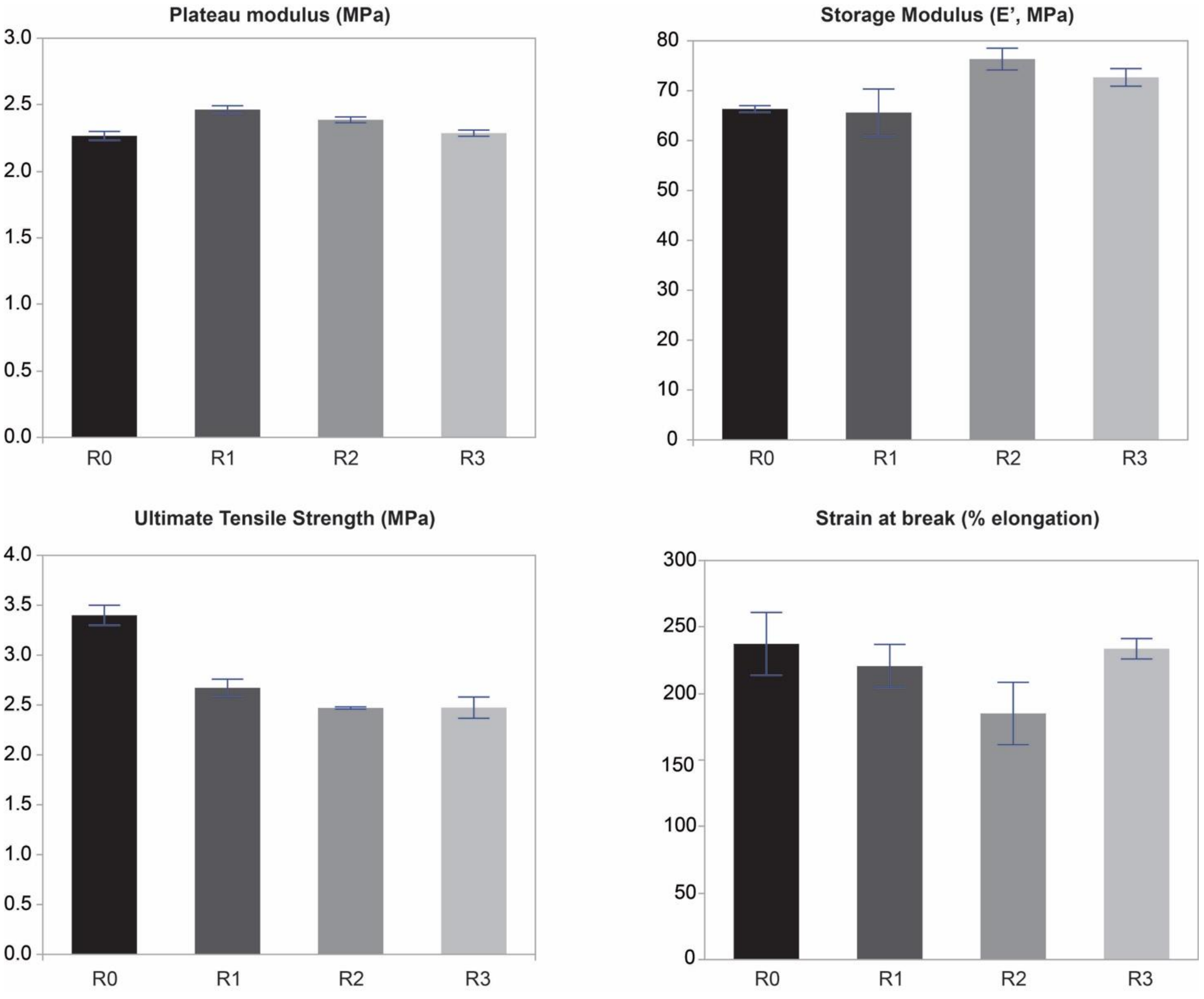

Figure S92: Summarized tensile properties of nHexDec samples after multiple rounds of reprocessing.

References:

1. Love, J. A., Morgan, J. P., Trnka, T. M. & Grubbs, R. H. A practical and highly active ruthenium-based catalyst that effects the cross metathesis of acrylonitrile. *Angew. Chemie - Int. Ed.* **41**, 4035–4037 (2002).

2. Yoon, K. H., Kim, K. O., Wang, C., Park, I. & Yoon, D. Y. Synthesis and structure-property comparisons of hydrogenated poly(oxanorbornene-imide)s and poly(norbornene-imide)s prepared by ring-opening metathesis polymerization. *J. Polym. Sci. Part A Polym. Chem.* **50**, 3914–3921 (2012).

3. Moatsou, D., Nagarkar, A., Kilbinger, A. F. M. & O'Reilly, R. K. Degradable precision polynorbornenes via ring-opening metathesis polymerization. *J. Polym. Sci. Part A Polym. Chem.* **54**, 1236–1242 (2016).

4. B. Chang, A. *et al.* Design, Synthesis, and Self-Assembly of Polymers with Tailored Graft Distributions. *J. Am. Chem. Soc.* **139**, 17683–17693 (2017).
